# The effect of air pollution on morbidity and mortality among children aged under five in sub-Saharan Africa: Systematic review and meta-analysis

**DOI:** 10.1371/journal.pone.0320048

**Published:** 2025-04-10

**Authors:** Eyasu Alem Lake, Joshua Karras, Guy B. Marks, Christine T. Cowie

**Affiliations:** 1 School of Nursing, College of Health Science and Medicine, Wolaita Sodo University, Sodo, Ethiopia; 2 South West Sydney Clinical Campus, UNSW Medicine and Health, University of New South Wales, Sydney, New South Wales, Australia; 3 Woolcock Institute of Medical Research, Macquarie University, Sydney, New South Wales, Australia; 4 School of Population Health, University of New South Wales, Sydney, New South Wales, Australia; 5 Ingham Institute for Applied Medical Research, Liverpool, New South Wales, Australia; Menzies School of Health Research: Charles Darwin University, AUSTRALIA

## Abstract

**Background:**

Air pollution from indoor and outdoor sources constitutes a substantial health risk to young children in sub-Saharan Africa (SSA). Although some systematic reviews have assessed air pollution and children’s respiratory health in SSA, none have considered both ambient and indoor exposures.

**Methods:**

This systematic review and meta-analysis assessed the effect of air pollution (ambient and indoor) on respiratory hospitalization and mortality among children under five years in SSA. We retrieved relevant articles from PubMed, Embase, Scopus, African Journals Online (AJOL), Web of Science, and medRxiv. The protocol was registered with Prospero (CRD42023470010). We used guidelines from the preferred reporting items for systematic review and meta-analysis (PRISMA-2020) to guide the systematic review process. Risk of bias was assessed using the Office of Health Assessment and Translation (OHAT) quality appraisal tool. For exposures where there were sufficient studies/data we conducted meta-analyses using random effects models and used Stata version 17 software for analysis.

**Results:**

For the systematic review we screened 5619 titles and abstracts, reviewed 315 full texts, and included 31 articles involving 2,178,487 participants. Eleven studies examined exposure to solid fuel use in households and its association with all-cause mortality, while four studies explored the impact of passive smoking on mortality among children under five. Only two studies assessed ambient air pollution's effects on all-cause and respiratory-related mortality. Additionally, 13 studies reported varying associations between respiratory hospitalization and household tobacco smoke exposure. Meta-analyses on studies of solid fuel use and mortality and passive smoking and hospitalizations showed that children exposed to indoor solid fuels combustion had higher odds of mortality compared to non-exposed children (OR = 1.31; 95% CI: 1.16–1.47). The meta-analysis of exposure to second-hand smoke found an increased risk of respiratory hospitalization due to pneumonia, although the results were not significant (OR = 1.29; 95% CI: 0.45–3.68), and our certainty of evidence assessment indicated insufficient support to conclusively establish this association.

**Conclusion and Recommendation:**

Our review reveals that solid fuel use and ambient PM_2.5_ exposure were associated with increased mortality risk in children under five years in SSA. The meta-analysis showed evidence of an increased risk of under-five years mortality associated with solid fuel use in households. Associations between secondhand smoke and pneumonia hospitalization were less clear. We conclude that significant research gaps remain in understanding the impact of discrete sources of air pollution on the causation of respiratory illness in young children living in SSA. Prioritizing interventions targeting indoor sources is essential, along with further studies which use standardized and objective exposure and outcome measures to study these associations.

## Introduction

Air pollution poses a multifaced environmental challenge that significantly impacts human health worldwide. It is defined by the World Health Organization (WHO) as the alteration of indoor or outdoor environments by any physical, chemical, or biological agent that disrupts the natural composition of the atmosphere [1]. Air pollution is detrimental to vulnerable populations, with children bearing a disproportionate burden. Their developing respiratory and immune systems, and proximity to ground-level pollutants render them particularly susceptible [2, 3]. Alarmingly, a recent WHO report showed that over 90% of children globally breathe polluted air that exceeds the WHO air quality guidelines [4].

Air pollution exposure is ranked as the fourth deadliest global risk factor for disease burden, surpassed only by dietary risks, tobacco, and high blood pressure. In 2019 alone, it was responsible for 6.67 million deaths and over 213 million disability-adjusted life years (DALYs) [5, 6]. Children’s exposure to air pollution reduces their life expectancy by an average of 20 months [6].

Hazardous exposure to air pollution disproportionally affects people residing in low and middle-income countries (LMIC), with over 95% of air pollution-related deaths occurring in LMICs in Africa and South Asia [7]. Africa has the highest excess mortality from air pollution from both indoor and outdoor origins, and respiratory infection was suggested to be a potential contributor [8–10]. Although household air pollution (HAP) has improved globally, it remains problematic in Africa, accounting for 697,000 air pollution-related deaths in 2019 [8]. The challenge of ambient air pollution, aggravated by rapid urbanization and industrialization, is remarkably pronounced in SSA, where infants and young children face adverse health outcomes due to their heightened vulnerability to environmental pollutants [8,11–13].

Epidemiological studies show substantial evidence of the relationship between air pollution and various adverse health consequences, including mortality, cancer, cardiovascular disease, and respiratory infections [14–18]. However, some studies in SSA also reported no or inconclusive associations [19, 20]. Existing systematic reviews on air pollution and adverse child health outcomes in SSA have mainly focussed on either a single country [21, 22] or specific sources of air pollution, such as HAP or its proxy [23], to the exclusion of ambient air pollution, which is also often an important source of hazardous exposure [24]. Others synthesized data either outside of our scope of respiratory hospitalization in under-five children [25] or in wider settings where data related to LMICs more broadly as part of global studies, both of which may not be fully relevant to the SSA region [14,26].

Household air pollution, primarily caused by the burning of solid fuels for cooking, lighting, and heating within homes, has far-reaching effects that extend beyond the immediate indoor environment. Pollutants generated from these activities contribute not only to household air pollution but also to ambient air pollution, which can infiltrate neighboring homes and communal spaces [27]. Hence, even dwellings that do not use biomass fuel for cooking and heating may be adversely affected by this practice within their communities. As a result, none of the existing systematic reviews provides a comprehensive understanding of the effect of indoor and ambient air pollution on respiratory morbidity and mortality among children under five years in SSA.

Hence, we aimed to (i) identify and collate relevant peer-reviewed epidemiological studies on air pollution and its effect on mortality among children aged under five years living in SSA; (ii) identify and collate relevant peer-reviewed epidemiological studies of air pollution and its effect on respiratory hospitalizations, such as acute respiratory infections (ARI), pneumonia, and asthma; (iii) quantify the association between air pollution and under-five mortality and respiratory hospitalization; and (iv) identify the existing research gaps in the evidence.

## Methods

In conducting this systematic review, we followed the preferred reporting items for systematic review and meta-analysis (PRISMA-2020) guideline [28]. Firstly, we prepared a protocol based on the PRISMA-Protocol statement [29, 30]. We registered the protocol in the Prospero (Prospective Register Of Systematic Review) database (ID: CRD42023470010) [31], an international database of systematic reviews conducted since 2011, to prevent redundancy and reduce the potential for reporting bias [32]. Since this review solely relied on aggregating and analyzing previously published studies and the data was de-identified, it did not require ethical approval.

### Source of information

We interrogated five databases and one internet site: Embase, PubMed, Scopus, Web of Science, African journals online(AJOL), and medRxiv [33]. We screened for all available literature held in each database until July 2024.

### Search strategy

We employed a three-step search to find preprints and published studies. First, we did a preliminary search of PubMed and Embase to identify relevant articles on the topic and help inform the search strategy. We used the text words in the title and abstract of relevant articles and the index terms used to describe the reports to develop a complete search strategy in each database and adapted the search accordingly. We also checked the reference list of included articles to find additional relevant articles for potential inclusion. Preprints were searched primarily on medRxiv, with PubMed and Embase also included due to their inclusion of preprints.

We used several keywords for the search strategy, including “air pollution,” “under-five,” “mortality,” and “sub-Saharan Africa.” Furthermore, several synonyms for each keyword were used for the search process, as shown below. The final search strategy, including the results, is available in a supplementary file (S2).

### Main keywords and alternative terms for the search strategy

- Air pollution (air quality, indoor air pollution, indoor air quality, outdoor air pollution, outdoor air quality, ambient air pollution, ambient air quality, household air pollution, household air quality, traffic-related air pollution * , particle * , particulate, PM_10_, PM_2.5_, carbon monoxide, “CO,” Sulfur dioxide, “SO2,” nitrogen dioxide, nitr* oxide*, “NO2,” “ozone,” “O3”, biomass, biofuel * , emission * , combustion, smoke)
- Under-five (baby, infant, child * , neonat * , fetal, foetal)
- Morbidity, Mortality (death, Respir * , pneumonia * , asthma, health, hospital * , “URTI”, “LRTI”)
- Sub-Saharan Africa (“SSA,” Angola, Benin, Botswana, (Burkina Faso), Burundi, (Cabo Verde) OR Cameroon, “Central African Republic,” Chad, Comoros, Congo, “Cote d'Ivoire,” “Equatorial Guinea,” Eritrea, Ethiopia, Gabon, Gambia, Ghana, Guinea, “Guinea- Bissau,” Kenya, Lesotho, Liberia, Madagascar, Malawi, Mali, Mauritania, Mauritius, Mozambique, Namibia, Niger, Nigeria, Rwanda, Senegal, Sierra Leone, Somalia, “South Africa,” “South Sudan,” Sudan, Tanzania, Togo, Uganda, Zambia, Zimbabwe)
*Acting as a wildcard character representing any sequenced search terms that could be available in the search engines

### Eligibility criteria

The eligibility criteria for studies were based on participants, exposures, comparators, outcomes, and study designs to identify studies assessing the effect of air pollution on mortality and respiratory hospitalization in children under five years within SSA. Only studies published in English were included in this systematic review and meta-analysis.

### Inclusion criteria

#### Participants.

All children under five years of age living in SSA. Studies were included only if the data for this age group were analyzed and reported separately.

#### Exposures.

Exposure was defined as ambient and indoor air pollution, including sources such as biomass combustion for cooking and heating, other household pollution sources, and traffic-related pollutants (e.g., proximity to major roads or traffic density). We included studies that quantified exposure through objective measurements of pollutants (e.g., particulate matter (PM2.5), nitrogen dioxide (NO2), and carbon monoxide (CO)), spatial proximity to pollution sources, and proxies such as household fuel type, stove usage, and tobacco smoke in the household.

### Comparators

The comparator group consisted of children under five years of age with lower or no exposure to ambient and indoor air pollution. This group included children residing in environments with lower levels of pollution, as indicated by reduced proximity to pollution sources (e.g., major roads, industrial areas), the use of cleaner cooking technologies (e.g., electric or liquefied petroleum gas (LPG) stoves instead of biomass stoves), and no exposure to household tobacco smoke or lower levels of specific pollutants, serving as the reference group.

#### Outcomes.

The primary outcome of interest was all-cause mortality. However, we included studies that defined outcome as cause-specific mortality and hospitalization for respiratory diseases including pneumonia, asthma, and other respiratory infections.

#### Study Designs.

Observational studies (cohort, cross-sectional, case-control, and ecological) and interventional studies (experimental and quasi-experimental) that quantitatively assessed the relationship between air pollution exposure and outcome of interest were considered.

#### Exclusion Criteria.

Studies were excluded if they did not focus on SSA, lacked specific data on children under five years of age, or did not provide objective data on air pollution exposure or outcomes related to mortality or hospitalization due to respiratory diseases, including pneumonia, asthma, and other respiratory infections. Non-original research articles, such as reviews, commentaries, conference abstracts, and studies lacking full-text availability, were also excluded.

#### Study selection criteria.

Following the search, we imported all identified records into Covidence (Veritas Health Innovation, Melbourne, Australia), an automation tool for managing and streamlining systematic reviews [34]. We removed duplications, and two reviewers ((EAL and JK) piloted the screening of articles on the first 50 articles within Covidence. This included title and abstract screening, risk of bias assessment, and data extraction. Where there were discrepancies in opinion, both reviewers discussed this, and an independent decision was sought from the two senior authors (GBM, CTC), where required. Two of the initial 50 articles met the final inclusion criteria.

Following the piloting, the same two reviewers (EAL and JK) screened the titles and abstracts for all articles according to the inclusion criteria. Any conflicts that arose during the title and abstract screening were resolved by discussion and with the involvement of a third reviewer (CTC and/or GBM). We retrieved potentially relevant studies in full and read them independently. Studies meeting the final selection criteria were retained for data extraction. For those excluded, reasons for exclusion were reported.

The appraisal process consisted of three steps: assessing the Risk of Bias (RoB) in each study, assessing the quality of evidence across studies, and evaluating the strength of evidence across studies. We adapted a quality appraisal tool from the OHAT using methodology developed by the National Institute of Environmental Health Science-National Toxicology Program (NIEHS-NTP) and the navigation guide from the University of California [35, 36]. The tool is recommended for evaluating the internal validity of environmental epidemiological studies. It comprises eight domains: recruitment strategy, exposure assessment, confounding control, outcome assessment, incomplete outcome, selective reporting, conflict of interest, and other biases. With each prescribed criterion, we rated each bias domain as “low,” “probably low,” “probably high,” “high,” and “non-applicable.” The same two reviewers performed the assessment independently. In the case of disagreement, the resolution began by seeking the opinion of a third reviewer (CTC and/or GBM). If we did not reach a consensus, further discussion ensued, and the final decision rested on the judgment of the most conservative voter. For example, if one reviewer judged “low” and the other judged “probably low,” the “probably low” rating was selected.

The critical appraisal process of each study concluded with assigning tiers to articles based on key appraisal domains (exposure assessment, confounding bias, and outcome assessment), selected based on OHAT recommendations, along with other domains such as recruitment strategy, incomplete outcome data, and selective reporting ( S5 File). A study judged either ‘low’ or ‘probably low’ for all key domains and most of the other domains ranked as tier one (good quality). A study judged either ‘high’ or ‘probably high’ for all key domains, and most of the other domains were classified as tier three (poor quality), and a study that was neither tier one nor tier three was ranked as tier two (medium quality). We conducted our meta-analysis with tier one and two studies and excluded tier three studies. EAL, with CTC and/or GBM, assessed evidence certainty using the OHAT approach, which tailors GRADE methodology for environmental health [37] (detail supplementary file, S7).

#### Data extraction.

We developed and piloted a data extraction template to ensure consistency in the information extracted (Table 2 and S4 File). The template included the following: (1) author, (2) study and publication year, (3) study area and country, (4) study design, (5) sample size, (6) response rate, (7) participant characteristics, (8) age of study participants, (9) pollutants or proxies of air pollution, (10) exposure assessment method, (11) outcome, (12) outcome assessment method, (13) potential confounders, (14) effect estimate, and (15) main findings.

**Table 2:** Characteristics of studies included in this systematic review and meta-analysis.

Author(s), publication year	Country, study year	Design Study size	Participant characteristics (age of children)	Pollutant exposure or proxy exposure	Comparator	Outcome: case-definition	Main results
All-cause mortality							
Akinyemi et al., 2016	15 SSA countries, 2010–2014 DHS	Cross-sectional N = 143602	Women with under five year old children (U5C) categorized into infants (birth–11 months) and children (12–59 months)	1. Maternal tobacco smoking 2. Solid fuels: coal, lignite, charcoal, wood, straw/shrubs/grass, dung, and crop residues	1. No smoking + non-solid fuel use in the household 2. No Smoking + solid fuel use in the household	1. Infant mortality: death between birth and 11 months of age 2. Under-five mortality: death between 12–59 months of age	Exposure to both solid cooking fuels and maternal tobacco smoking significantly increased the risk of infant mortality (HR = 1.71, 95% CI: 1.29–2.28). The risk of under-five mortality was higher in children exposed to both solid cooking fuels and maternal tobacco smoking (HR = 1.99, 95% CI: 1.28–3.8).
Bickton et al., 2020	14 SSA countries, 2015–18 DHS	Cross-sectional N = 164376	Women with U5C (0–59 months)	1. Only charcoal 2. Other biomass fuels: wood, dung, kerosene, coal, crop residues, shrubs 3. Kitchen Location: in the house, outdoor	1. Clean fuels: natural gas, biogas, LPG, and electricity 2. Kitchen located in the separate building	Under-five mortality (U5M): death between birth to 59 months of age	U5M risk was higher for those using biomass fuels (OR = 1.32, 95% CI: 1.00–1.74) or charcoal (OR = 1.35, 95% CI: 1.07–1.71) compared to clean fuel users. Cooking outside (OR = 0.75, 95% CI: 0.64–0.87) or in separate buildings (OR = 0.85, 95% CI: 0.73–0.98) reduced U5M risk compared to cooking indoors.
Egondi et al., 2018	Kenya, 2003–13	Semi-ecological N = 21641	Mother with children (below five years)	Outdoor M_2.5_ (PM_2.5 ≥ _25 µg/m3) measured by DustTrak handheld sampler	PM_2.5_ < 25 µg/m3	All-cause U5M: maternal reporting death between birth to 59 months of age	U5C exposed to PM_2.5_ > 25 µg/m³ had a 22% higher risk of all-cause U5M (IRR = 1.22, 95% CI: 1.08–1.39) compared to those exposed to PM_2.5_ < 25 µg/m³.
Ezeh et al., 2014	Nigeria, 2013 NDHS	Cross-sectional N = 30726	Women with U5C categorized as neonates (0–28 days), post-neonates (1–11 months), and children (12–59 months)	Solid fuels: coal, charcoal, wood, agricultural crop, animal dung, straw/shrubs/grass	Non-solid fuels: electricity, natural gas, LPG, biogas, and kerosene	1. Neonatal mortality: death between birth and 28 days of age 2. Post-neonatal mortality: death between 1–11 month of age 3. Child mortality: death between 12–59 months of age	82% of neonatal, 90% of post-neonatal, and 94% of child deaths occurred in solid fuel-using households. Population Attributable Risk (PAR); Neonates: 0.8% (95% CI: -7.8–2.8); post-neonates: 42.9% (95% CI: 31.9–61.4); Children (12–59 months): 36.3% (95% CI: 33.1–52.1). Higher mortality in rural and economically disadvantaged households.
Flanagan et al., 2022	Ethiopia, 2015–18 EDHS	Prospective cohort N = 2085	Pregnant women (0–30 days)	1. Ambient NO_2_ and NOx exposure based on LUR models (every 10 µg/m³ increase) 2. Solid fuel: wood, charcoal, cow dung 3. Mixed fuel: Solid and clean (electricity, gas/kerosene, LPG) fuels NO_2_	1. Lower Ambient NO_2_ and NOx exposure: baseline or reference concentration (29.9 µg/m³ (NOX), 13.7 µg/m³ (NO2)) 2. Clean fuels: electricity, gas/kerosine, and LPG only	Neonatal mortality: death within the first 30 days of age	Neonatal mortality was not significantly associated with solid or mixed fuel use (OR = 0.61, 95% CI: 0.20–1.85) compared to clean fuel use. Each 10 µg/m³ rise in ambient NO_2_ and NOx during pregnancy was associated with a lower risk of neonatal mortality: NO2: OR = 0.91 (95% CI: 0.32–2.58); NOx: OR = 0.72 (95% CI: 0.39–1.76).
Heathfield et al., 2020	South Africa, 2013–17	Retrospective Cross-sectional N = 1608	Medical records with infant death (below one year)	Passive tobacco smoking in the house	No passive tobacco smoking in the house	Sudden unexpected death of infants (SUDI): infant deaths admitted for medico-legal investigation and classified as SUDI	From total mortality (n = 1,608)74.56% of infant admissions for mortuary diagnosis (n = 1,608) were classified as SUDI. Over 53% of SUDI cases were exposed to tobacco smoke, primarily from maternal (68.88%) or other household sources (32.12%).
Imo et al., 2023	Nigeria, 2018 NDHS	Cross-sectional N = 124442	Women with U5C (0–5 years)	Solid fuel: coal/lignite, wood, charcoal, straw/shrubs/grass, agricultural crops, animal dung	Non-solid fuel: Electricity, gas, and kerosene	U5M: dying between birth and 59 months of age	86% of mothers (n = 107,692) used solid fuel for cooking with both neighborhood poverty (HR: 1.44, 95% CI: 1.34–1.54) and the use of solid cooking fuel in the household (HR: 2.26, 95% CI: 2.06–2.49) were independent predictors of U5M.
Kleimola et al., 2015	27 SSA countries, 2005–12 DHS	Cross-sectional N = 418622 (neonatal) N = 404254 (U5C)	Women with U5C (categorized as birth–28 days and29 days to 59 months)	1. Solid fuels: coal, charcoal, biomass like wood, crop waste, and dung 2. Kerosene	Clean fuel: electricity, natural gas, and biogas	1. Neonatal mortality: death within the first 28 days of age 2. U5M: death from 29 days to 59 months of age	No significant increased risk for neonatal or U5M in homes using solid fuels (Neonates: RR = 1.02, 95% CI: 0.92–1.13; U5M: RR = 1.06, 95% CI: 0.92–1.21). Kerosene use was associated with a 22% higher risk of neonatal mortality (RR = 1.22, 95% CI: 1.01–1.48) but not U5M (RR = 0.91, 95% CI: 0.78–1.07).
Latona et al., 2017	Nigeria, 2008 NDHS	Cross-sectional N = 28647	Women with U5C (below 5 years)	Biomass cooking fuels	Non-biomass fuels	U5M: death of a child at the end of five years of age	Non-biomass fuel use reduced U5M risk by 44% compared to biomass fuels (HR = 0.448, 95% CI: 0.35–0.57).
Owili et al., 2017	23 SSA countries, 2010–14 DHS	Cross-sectional N = 783691	Women with U5C (0–5 years)	1. Charcoal 2. Other biomass: wood, agricultural crops, dung, straw/shrubs/grass 3. Other cooking fuel: coal, lignite, paraffin/kerosene 4. Kitchen location 5. Tobacco smoking in the house	Clean fuel: electricity, LGP, natural gas or biogas	U5M: death of under-five child aged between birth to 59 months	Increased U5M risk with charcoal (HR = 1.21, 95% CI: 1.10–1.34), other biomass (HR = 1.20, 95% CI: 1.08–1.32), and in-house kitchens (HR = 1.06, 95% CI: 1.03–1.10). Tobacco smoke exposure raised U5M risk by 9% (HR = 1.09, 95% CI: 1.06–1.12). No significant interaction was detected between biomass cooking fuel and smoking (RER_HR_ = 0.12; 95% CI: -0.21–0.45).
Samuel et al., 2018	Nigeria, 2013 NDHS	Cross-sectional N = 10983	Women with U5C (0–59 months)	1. Solid fuel: wood, dung, charcoal	Clean fuel: electricity, LPG, or kerosene	U5M: death of under-five child aged between birth to 59 months	Solid fuel use was reported in 71.3% of households, three times higher in rural areas than urban. Wealth and education strongly influenced solid fuel use and U5M. Solid fuel use was linked to increased but non-significant odds of U5M (OR = 1.23, 95% CI: 0.98–1.54).).
Shifa et al., 2018	Ethiopia, 2011–14	Case-control N = 381 (cases) N = 762 (controls)	U5C (below five years)	1. Lack of a separate kitchen for cooking in the household 2. Non-electric light sources in the household	1. Have a separate kitchen for cooking 2. Use of electricity for lighting	1. Infant mortality: the probability of dying before the first birthday 2. U5M: the probability of dying before the fifth birthday	Lack of a separate kitchen increased the risk for U5M (OR = 1.77, 95% CI: 1.16–2.70) and infant mortality (OR = 1.94, 95% CI: 1.13–3.33). Electricity as a light source reduced infant mortality risk (OR = 0.47, 95% CI: 0.43–0.99) and had a non-significant effect on U5M (OR = 0.87, 95% CI: 0.48–1.27).
Shiferaw et al., 2023	Ethiopia, 2016 EDHS	Cross-sectional N = 10452	Women with U5C (below five years)	A 10-unit increase in ambient PM_2.5_ as measured by the Washington Atmospheric Composition Analysis Group (ACAG) using satellite remote sensing	Baseline PM_2.5_ value (reference group)	U5M: death of child before the age of 60 months	U5M was 5.4% (95% CI: 5.0–6.8%). Each 10-unit increase in ambient PM_2.5_ (20.1 µg/m³) increased U5M risk (OR = 2.29, 95% CI: 1.44–3.65).
Starnes et al., 2023	Kenya,2021	Cross-sectional N = 15999	Household living U5C (below five years)	Indoor cooking stove without ventilation	Indoor cooking stoves with proper ventilation or outdoor cooking	U5M: maternal reported death before reaching the fifth birthday	The U5M rate was 32.2 per 1000 live births. Indoor smoke exposure was significantly associated with increased U5M risk (HR = 1.91, 95% CI: 1.08–3.42).
Wichmann et al., 2006	South Africa, 1998 SADHS	Cross-sectional N = 3556	Women with U5C (1–59 months)	1. Solids fuels: wood, dung, coal, paraffin, and in combination with clean fuels (LPG, natural gas, electricity)	Clean fuel: LPG, natural gas, or electricity only	U5M: women reported they had given birth but died before reaching the fifth birthday	U5M rate was higher in households using polluting fuels (1.695 per 1000 person-months) compared to clean fuels (0.659 per 1000 person-months). Polluting fuel use increased U5M risk (RR = 1.99, 95% CI: 1.04–3.68).
Winterbach et al., 2021	South Africa, 2012–16	Retrospective Cross-sectional N = 440	Archives with SUDI admission (below one year)	Have a history of tobacco smoke	No history of maternal/paternal tobacco smoking	SUDI: mortuary-registered cases manually searched in the hospital archive for all infant deaths (under 1 year)	SUDI accounted for 38.56% of infant mortalities, with a rate of 7.95 per 1000 births. Respiratory tract infections were the leading cause of SUDI (74.1%). 62.9% of SUDI cases were exposed to cigarette smoking, with antenatal smoking significantly associated with SUDI (p = 0.004).
Cause-specific mortality							
Egondi et al., 2018	Kenya, 2003–13	Semi-ecological N = 21641	Mother with U5C (below five years)	Outdoor PM_2.5_ > 25µg/m3 measured by/using DustTrak II 8532 hand-held samplers	Outdoor PM_2.5_ < 25 µg/m3	Respiratory caused U5M: maternal-reported cough at each survey and death before 5 years of age during follow-up	Exposure to PM_2.5_ > 25 µg/m³ was associated with a 22% increased risk of respiratory cause mortality (IRR = 1.22, 95% CI: 0.88–1.42) compared to PM_2.5_ < 25 µg/m³.
Francisco et al., 1993	Gambia, 1990	Cases-control N = 129 (cases) N = 144 (dead controls) N = 270 (live controls)	Mother/guardian of child (less than two years)	1. Parental tobacco smoking 2. Mother carried child while cooking 3. Index of indoor air pollution > 6 (the index score for indoor air pollution was based on specific factors: kitchen location, type of fire used, whether the child was carried during cooking, and parental smoking habits. An index score of six or higher indicates significant indoor air pollution)	1. No parental smoking in the household 2. Never carried a child while cooking 3. Index of indoor air pollution < 3	ALRI death: ALRI confirmed death by consensus of at least two out of three independent physicians for children under five years of age.	Parental smoking was significantly associated with higher odds of ALRI death (both parents: OR = 3.67, 95% CI: 1.09–12.42; one parent: OR = 1.57, 95% CI: 0.96–2.56). Exposure to indoor air pollution index > 6 showed negligible risk for ALRI death (OR = 1.0, 95% CI: 0.49–2.05). Mothers who carried their child while cooking had higher odds of ALRI death (always: OR = 5.23, 95% CI: 1.72–15.92; sometimes: OR = 1.47, 95% CI: 0.54–4.02).
Johnson et al., 1992	Nigeria, 1985–86	Case-control N = 103 (cases) N = 103 (control)	Mother/guardian of child (2 weeks - 59 months)	1. Habit of cigarette smoke in the house 2. Cooking using firewood	1. Uknown status of cigarette smoke exposure 2. Other cooking fuels	ALRI deaths: death in children aged 2 weeks to 59 months with hospital-diagnosed ALRI (croup, bronchiolitis, pneumonia, empyema) and illness ≤ 28 days.	ALRI deaths were linked to exposure to wood smoke, with 63% of ALRI deaths (5/8) occurring in children exposed to wood smoke. Other exposures included kerosene stoves (n = 79; 77%), wood smoke (n = 16; 16%), gas combustion (n = 5; 5%), and household cigarette smoke (n = 17; 17%).
Johnson et al., 2008	Nigeria, a 30-month follow-up study (study year not reported)	Prospective Cross-sectional N = 419	Mother/guardian of child (2 weeks - 59 months)	1. Cooking fuels: Kerosene ^+^ gas, wood ^+^ kerosene 2. Kitchen location: inside, corridor, separate 3. Tobacco smoker in the house	1. Unknown cooking fuel 2. Unknown kitchen location 3. No tobacco smoke exposure in the house	Community-acquired pneumonia (CAP)-associated mortality: death of children aged 2 weeks to 5 years with ALRI symptoms, as per Denny and Clyde, and radiographically confirmed pneumonia	Among ALRI cases (n = 419), pneumonia (77%), bronchopneumonia (72.4%), and lobar pneumonia (20.4%) were the most common diagnoses. Wood smoke exposure (16.7% of cases) was significantly associated with increased CAP-associated mortality (χ² = 7.45, p = 0.006).
Respiratory hospitalization							
Dano et al., 2019	Niger, 2015–16	Cross-sectional N = 637	Parents with children (1–59 months)	Passive tobacco smoke in the house	No passive tobacco smoke in the house	Carriage of *S.pneumonia*: a laboratory diagnosis by nasopharyngeal swabs and RT-PCR	Of the 637 study subjects, 76% carried respiratory viruses, 47% carried bacteria, and 42% carried both. Carriage was not linked to socio-demographic factors or passive smoking (OR = 0.73; 95% CI:0.47–1.12).
Fakunle et al., 2014	Nigeria, 2012	Case-control N = 220 (cases) N = 220 (controls)	Mother with U5C (below 5 years)	1. Firewood for cooking 2. Lantern smoke 3. Tobacco smoking in the house 4. Carrying the child while cooking	The comparator is not explicitly stated for each exposure	ARI: WHO ARI definition for U5C	Use of lanterns (OR = 2.1, 95% CI: 1.7–42) and firewood for cooking (OR = 2.21, 95% CI: 1.7–7.0) were independent predictors of ARIs. Parental smoking was associated with increased ARI risk, though not statistically significant (OR = 1.61, 95% CI: 0.07–36.8).
Johnson et al., 1992	Nigeria, 1985–86	Case-control N = 103 (cases) N = 103 (control)	Mother/guardian of child (2 weeks - 59 months)	1. Cigarette smokers 2. Firewood cooking	1. Uknown status of cigarette smoke exposure 2. Other cooking fuels	The outcome of ARI hospitalization: Clinician diagnosis of ARI	Household cooking fuel type was strongly associated with ALRI hospitalization (*X*^2^ = 12.21; p < 0.005), but cigarette smoking was not (chi-square “χ²” = 0.46; p = 0.5).
Johnson et al., 2008	Nigeria, a 30-month follow-up study (no study year found)	Prospective Cross-sectional N = 419	Mother/guardian of child (2 weeks - 59 months)	1. Cooking fuels: Kerosene ^+^ gas, wood ^+^ kerosene 2. Kitchen location: inside, corridor, separate 3. Tobacco smoker in the house	1. Unknown cooking fuel 2. Unknown kitchen location 3. No tobacco smoke exposure in the house	Lobar pneumonia (LPA) and Bronchopneumonia (BPA): diagnosed by radiographic consolidation and/or other intrathoracic complications. .	The distribution of ALRI (n = 419) included pneumonia (n = 323;77%), BPA (n = 234;72.4%), LPA (n = 66,20.4%) and both BPA and LPA (n = 23,7.1%). Among all ALRI cases, 16.7% were exposed to smoke, which was associated with higher mortality. Parental smoking was significantly higher amongst the LPA group category (p = 0.04; RR = 1.19).
Kiconco et al., 2021	Uganda, 2019	Cross-sectional N = 336	Parents/caretakers with children (2–59 months)	1. Indoor cooking 2. Parental/caregiver smoking	1. Outdoor cooking 2. No parental/caretaker smoking	Pneumonia: WHO/IMNCI case definition for pneumonia and confirmed by chest X-ray (infiltrates, consolidation, pleural effusion)	Children exposed to cigarette smoking had three times higher odds of pneumonia (OR = 3.00, 95% CI: 1.35–6.80). Outdoor cooking was not associated with pneumonia (OR = 0.9, 95% CI: 0.54–1.53).
Nantanda et al., 2013	Uganda, 2011–12	Cross-sectional N = 614	Caretakers with children (2–59 months)	1. Exposure to tobacco smoking 2. Use of gas for cooking	1. No exposure to tobacco smoking 2. No use of gas for cooking	1. Asthma: diagnosed by modified global initiatives for asthma (GINA) guidelines 2. Bronchiolitis: defined based on South African bronchiolitis case management guideline; both diagnoses confirmed by an expert panel.	Asthma was significantly associated with gas cooking (OR = 3.8, 95% CI: 1.2–13.3) and, to a lesser extent, tobacco smoke (OR = 1.5, 95% CI: 0.8–2.7). Bronchiolitis was not significantly associated with tobacco smoke (OR = 1.1, 95% CI: 0.6–2.0) or gas cooking (OR = 1.4, 95% CI: 0.5–4.5).
Ngocho et al., 2019	Tanzania, 2017	Case-control N = 113 (cases) N = 350 (control)	Parents with U5C (2–59 months)	1. Unclean cooking fuels: kerosene, biomass, firewood, charcoal 2. Tobacco smoking in the house	1. Clean cooking fuels 2. No tobacco smoke exposure in the house	Pneumonia: hospitalized children aged 2–59 months with cough, fast breathing (WHO case definition criteria), and X-ray-confirmed	Children exposed to unclean cooking fuels had 80% higher odds of CAP (OR = 1.8, 95% CI: 1.0–3.3). Exposure to second-hand smoke was not significantly associated with pneumonia (OR = 0.4, 95% CI: 0.2–1.0).
PrayGod et al., 2016	Tanzania, 2013–14	Case-control N = 42 (cases) N = 72 (controls)	Parents/guardians with children (2–59 months)	1. Cooking fuel: firewood, wood charcoal 2. Tobacco smoking exposure 3. Outdoor cooking	1. Cooking fuel: gas/electricity 2. No tobacco smoke exposure 3. Cooking indoor	Severe pneumonia: WHO case definition for severe/very severe pneumonia in children aged 2–59 months	Severe pneumonia was five times more likely in children cooking indoors (OR = 5.5, 95% CI: 1.4–22.1). Exposure to firewood (OR = 2.1, 95% CI: 0.2–27) and wood charcoal (OR = 1.9, 95% CI: 0.2–28) showed non-significant associations. Household smoking showed no significant association with severe pneumonia (OR = 0.6, 95% CI: 0.1–3.2).
Roux et al., 2015	South Africa 2012-14	Cohort N = 1000	Mother-infant pairs < 1 year of age	Maternal smoking in the household	No maternal smoking in the household	Pneumonia: diagnosed per WHO guidelines based on cough, breathing difficulty, lower chest wall indrawing, or age-specific tachypnea; severe cases included danger signs	The study reported 141 pneumonia episodes (0.27 per child-year), with maternal smoking associated with higher pneumonia risk (OR = 1.94, 95% CI: 1.32–2.28).
Roux et al., 2021	South Africa 2012-15	Cohort N = 1143	Mother-child pairs	Indoor PM_10_ > 40 μg/m3 measured using an air sampling pump Maternal smoking > 500 ng/ml as measured by urine cotinine	1. Indoor PM_10_ < 40 μg/m3 2 No maternal smoker/Passive smoker: < 500 ng/ml	Pneumonia: WHO based on cough, breathing difficulty, age-specific tachypnea (≥50 breaths/min for 2–12 months, ≥ 40 for > 12 months) or chest indrawing.	Among 174 hospitalized children, maternal smoking (43%) and indoor air pollution (61%) were prevalent. Elevated antenatal PM_10_ levels were associated with a threefold increased risk of serious outcomes (OR = 3.17, 95% CI: 0.38–26.46), though the estimate had limited precision due to small sample size.
Tazinya et al., 2018	Cameroon, 2014–15	Cross-sectional N = 512	Parents/guardians with children (2–59 months)	1. Wood smoke 2. Tobacco smoking in the house	1. No exposure to woodsmoke 2. No tobacco smoke exposure in the house	ARI: defined based on IMNCI ARI classification for children	Exposure to wood smoke (OR = 1.85, 95% CI: 1.22–2.78) and passive smoking (OR = 3.58, 95% CI: 1.45–8.84) were identified as significant risk factors for ARIs.
Ujunwa et al., 2014	Nigeria, 2007–08	Cross-sectional N = 436	Parents/caregivers with children (<5 years)	1. Wood biofuel 2. Tobacco smoking exposure in the house	1. No exposure to wood biofuel 2. No exposure to tobacco smoking in the house	ARI, Pneumonia, Bronchitis: defined based on WHO case management for ARI, pneumonia, and bronchiolitis	Risk factors for pneumonia included passive smoking (RR = 1.39, 95% CI: 1.05–1.83) and biofuel use (RR = 2.09, 95% CI: 1.39–3.14). For bronchitis, passive smoking (RR = 0.35, 95% CI: 0.13–0.99) and biofuel use (RR = 1.09, 95% CI: 0.50–2.39) were identified as risk factors. For ARI, biofuel use was associated with a reduced risk (RR = 0.74, 95% CI: 0.64–0.85).
Vanker et al., 2017	South Africa, 2013–15	Cohort N = 1137 mothers with 1143 live births	Mother-infant pairs followed at 6–10 weeks, 14 weeks, 6, 9 and 12 months.	1.PM_10_ > 40 μg/m³/year measured using a personal air sampling pump (AirChek) 2. CO > 30 mg/m³/year measured using Altair (Troy) 3. Nitrogen dioxide > 40 μg/m³/year and volatile organic compounds (benzene and toluene > 240 μg/m³/year) measured in diffusion tubes in homes 4. Maternal smoking	1. PM_10_ < 40 μg/m³/year 2. CO < 30 mg/m³/year 3.Nitrogen dioxide < 40 μg/m³/year 4. No maternal smoking	LRTI or wheezing illness: defined using WHO case definition criteria	LRTI risk was significantly associated with antenatal maternal smoking (IRR = 1.62, p = 0.004) and PM exposure (IRR = 1.43, p = 0.008). Toluene exposure increased the odds of LRTI hospitalization (OR = 5.13, p = 0.012). Wheezing was linked to both antenatal (IRR = 2.09, p < 0.0001) and postnatal maternal smoking (IRR = 1.27, p = 0.024), as well as passive smoke exposure.
Verani et al., 2016	South Africa, 2010–12	Case-control N = 889 (cases) N = 2628 (control)	Parents/guardians with children (<5years)	Tobacco smoking in the house	No tobacco smoke exposure in the house	Presumed bacterial pneumonia (PBP): hospitalized children diagnosed through radiographics, and CRP ≥ 40 mg/L	Caregiver smoking was more common in cases, with 47 (5.3%) cases and 29 (1.1%) controls having a smoker in the household. U5C with a caregiver who smoked were five times more likely to experience PBP (OR = 5.15; 95% CI: 2.94–9.03).% CI: 2.94–9.03).

Footnote: **SSA:** Sub-Saharan Africa; **DHS:** Demographic and Health Surveys; **U5C:** Under-5 Child; **CI:** Confidence Interval; **HR**: Hazard atio; **U5M**: Under-5 Mortality; **HAP:** Household Air Pollution; **OR**: Odds Ratio; LPG: Liquefied Petroleum Gas; **PM**_**2.5**_: Particulate Matter with diameter ≤  2.5 micrometres; **WHO:** World Health Organization; **IMNCI:** Integrated Management of Newborn and Childhood Illness**; IRR**: Incidence Rate Ratio; **NDHS**: Nigeria Demographic and Health Survey; **PAR**: Population Attributable Risk; **EDHS**: Ethiopia Demographic and Health Survey; **LPG**: Liquefied Petroleum Gas; **SUDI:** Sudden Unexpected Death in Infancy; **RR**: Relative Risk; **RERHR**: Relative Excess Risk due to Interaction; **ACAG**: Atmospheric Composition Analysis Group; **ALRI**: Acute Lower Respiratory Infections; **CAP**: Community-Acquired Pneumonia; **BPA**: Bronchopneumonia; **LPA**: Lobar Pneumonia; **ARI**: Acute Respiratory Infections; **LRTI:** Lower Respiratory Tract Infection; **PBP**: Presumed Bacterial Pneumonia

#### Measurements of exposure and outcomes.

In this review, air pollution exposure was defined as exposure to either ambient or indoor air pollution, or both, assessed through direct measurement of pollutants or inferred using proxy indicators. Proxies for indoor air pollution included specific markers such as the presence of biomass stoves, tobacco smoking in the household, and other household emission sources, whereas proxies for ambient air pollution were characterized using metrics such as traffic counts and proximity to major roadways.

The primary outcome of this review was all-cause mortality, while the secondary outcomes were cause-specific mortality and respiratory hospitalizations, including pneumonia, asthma, and other respiratory illnesses, all in children under five years of age. This systematic review included the following outcomes: neonatal mortality (death within the first 28 days of life), post-neonatal mortality (death between 28 days and one year), infant mortality (death before one year, encompassing both neonatal and post-neonatal mortality), early childhood mortality (death of a child aged 12–59 months), and hospitalization (defined as the admission of children under-five years to a hospital or a health facility for medical care due to respiratory infections including ARI, pneumonia, bronchitis and asthma) [38].

#### Data analysis and synthesis.

We employed a narrative synthesis to summarise texts, tables, and figures as necessary and used descriptive statistics to summarise and describe the key characteristics and findings of the eligible studies.

We conducted a meta-analysis of available quantitative data relevant to the objectives. The extracted data was exported to Stata version 17 (StataCorp LP, 4905, Lakeway Drive, College Station TX77845, USA) for analysis. We extracted the effect estimates reported in each study (that is, hazard ratio (HR), relative risk (RR), or odds ratio (OR)) and their 95% confidence intervals (95% CI). To estimate the pooled effect size, we applied the following formula to convert different effect measures to a common measure, the OR, which was the most commonly reported in the studies [39].


$$\text{RR}=\frac{\left(1-\text{eHRln}\left(1-\text{r}\right)\right)}{\text{r}};\text{RR}=\frac{\text{OR}}{\left(1-\text{P}0+\left(\text{P}0\text{*OR}\right)\right)};\text{OR}=\frac{\left(\left(1-\text{P}0\right)\text{*RR}\right)}{\left(1-\text{RR*P}0\right)}\text{Where}$$

RR was a relative risk, r was the event rate for the reference or control group, HR was the hazard ratio, OR was the odds ratio, and P_0_ was the event rate in the unexposed population.

We assessed the variation across the studies using I^2^ statistics [40, 41]. Since we found considerable heterogeneity (I^2^ =  78.6%), we used the random effects model to estimate the weighted inverse-variance pooled effect estimate [40]. Odds ratios (ORs) were log-transformed to stabilize variances and create symmetrical confidence intervals. The log-transformed ORs were combined, and the final combined estimate was exponentiated for interpretation [42]. To check publications bias, we produced funnel plots and contour-enhanced funnel plots for graphical diagnosis of small study effects and, more objectively using the Egger regression test with a P-value < 0.05 as the test statistic to indicate the presence of publication bias [43, 44]. We also conducted a trim-and-fill analysis to further detect and adjust for publication bias by imputing missing studies [45]. We conducted subgroup analysis by study setting and year and also performed sensitivity analysis based on study quality to determine the source of heterogeneity. We also ran a leave-one-out sensitivity analysis to see the effect of a single study on the overall meta-analysis estimates [46].

## Results

### Systematic review

**Fig 1:**
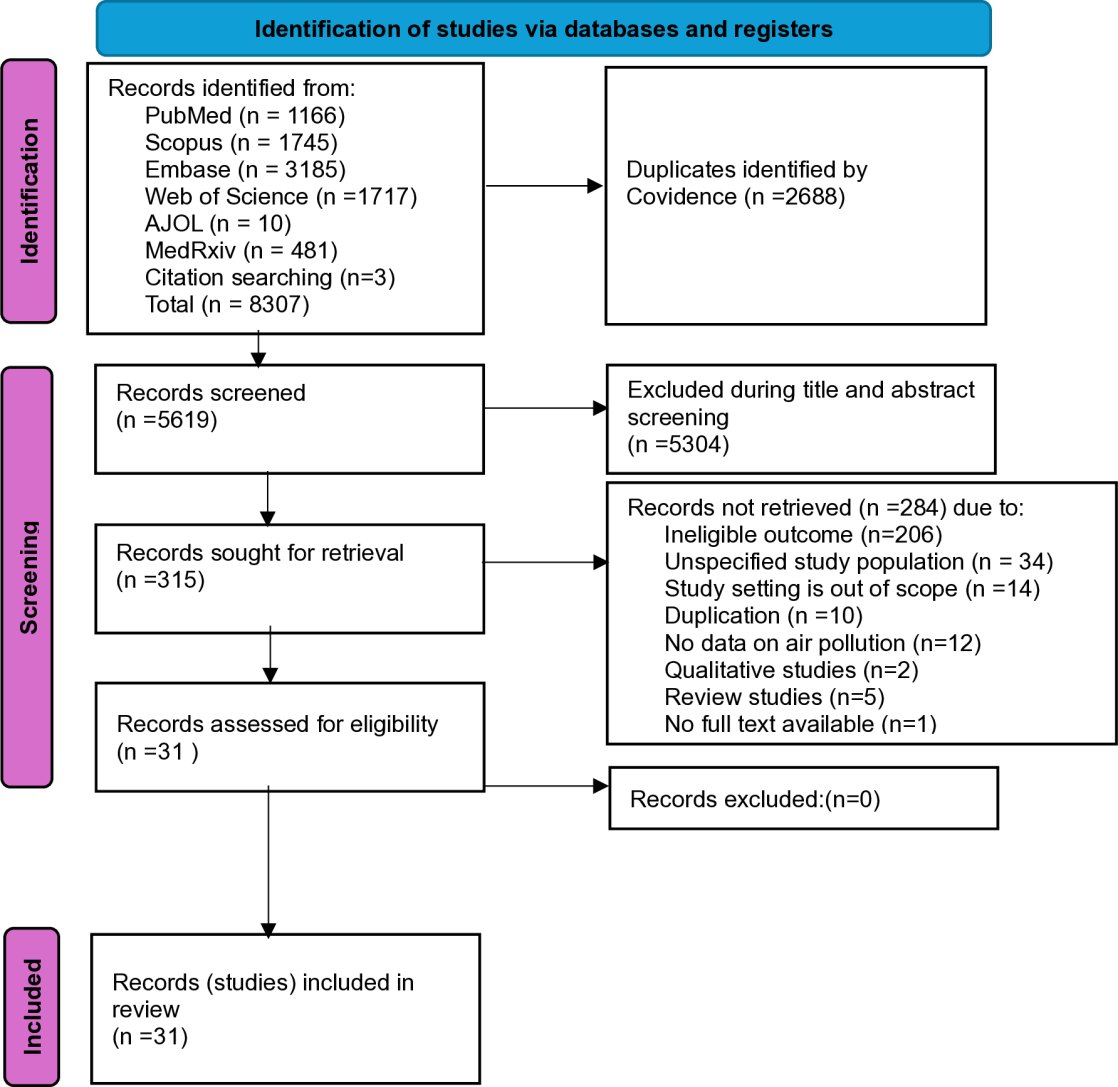
PRISMA 2020 flowchart for the study selection process.

We identified a total of 8307 records through the electronic search of online databases: Embase (n =  3185); PubMed (n =  1166); Scopus (n =  1745); Web of Science (n = 1717); AJOL (n =  10); medRxiv (n =  481), and citation search (n = 3). We removed duplicates (n = 2688) and screened 5619 articles by title, abstract, and keywords against the inclusion criteria. Of these, 315 articles were retained for full-text screening. During full-text screening, we excluded 284 articles for the following reasons: 1) the outcome was unrelated to our inclusion criteria (n = 206); 2) the study setting was beyond our geographic scope (n = 14); 3) both young and older children were included in the study population, and data was not provided separately for children under-five years (n = 34); 4) the study did not report air pollution data or household fuel use or any measure of air pollution exposure (n = 12); 5) the study was qualitative or a review (n = 7); 6) the study was a duplicate (n = 10); and 7) the full text was unavailable (n = 1). Finally, we included the 31 articles in this systematic review (Fig 1, supplementary file S3 and S4).

### Risk of bias assessment

We assessed the risk of bias for each of the 31 studies using eight predefined criteria (Table 1 and S5 File). The majority of study components (43.55%, 108 of 248) were rated as having ‘probably low’ risk of bias (ROB), followed by ‘low’ (30.65%, 76 of 248), and ‘probably high’ (24.19%, 60 of 248) ROB. Four (1.6%) study components (all related to confounding) scored a ‘high’ ROB judgment. However, the overall quality for those four studies was defined as tier 2 (moderate quality). Therefore, we did not exclude any studies from our meta-analysis based on the ROB assessment. All 31 studies except one were rated as Tier Two (moderate quality) in the overall ROB assessment.

**Table 1:** Risk of bias assessment results for 31 eligible studies.

Author(s)y/year	Recruitment strategy	Exposure assessment	Outcome assessment	Confounding	Incomplete outcome data	Selective reporting	Conflict of interest	Other biases	Overall assessment
Akinyemi et al./ 2016									Tier two
Bickton et al. /2020									Tier two
Dano et al. /2019									Tier two
Egondi et al./2018									Tier two
Ezeh et al. /2014									Tier two
Fakunle et al. /2014									Tier two
Flanagan et al. /2022									Tier two
Francisco et al. /1993									Tier two
Heathfield et al. /2020									Tier two
Imo et al. /2023									Tier two
Johnson et al. /1992									Tier two
Johnson et al. / 2008									Tier two
Kiconco et al. /2021									Tier two
Kleimola et al. /2015									Tier two
Latona et al. /2017									Tier two
Nantanda et al. /2013									Tier two
Ngocho et al. /2019									Tier two
Owili et al. /2017									Tier two
PrayGod et al. /2016									Tier two
Roux et al./2016									Tier two
Roux et al./2021									Tier two
Samuel et al. /2018									Tier two
Shifa et al. /2018									Tier two
Shiferaw et al. /2023									Tier two
Starnes et al. /2023									Tier two
Tazinya et al. /2018									Tier two
Ujunwa et al. /2014									Tier two
Vanker et al., 2017									Tier one
Verani et al. /2016									Tier two
Wichmann et al. /2006									Tier two
Winterbach et al. /2021									Tier two
**Risk of bias rating**	*Low*		*Probably Low*		*Probably high*		*High*	*NA*	

### General characteristics of eligible studies

Studies which met the inclusion criteria were from Nigeria (n = 8; 25.81%)[47–54], South Africa (n = 7; 22.58%)[55–61], SSA as a whole (n = 4; 12.91%) [18,19,62,63], Ethiopia (n = 3; 9.68%) [20,64,65], Kenya (n = 2; 6.45%) [66, 67], Tanzania (n = 2; 6.45%) [68^,^ 69], Uganda (n = 2, 6.45%) [70^,^ 71], Cameroon (n = 1; 3.23%)[72], Gambia (n = 1; 3.23%) [73], and Niger (n = 1; 3.23%) [74]. Most studies (n = 19) were cross-sectional in design, followed by case-control (n = 7), cohort (n = 4), and semi-ecological (n = 1) study designs. The studies were conducted between 1985 and 2020, but only two were published before 2010. The sample size ranged from 114 to 404,254 people. The majority of the studies analyzed population-based secondary data, with authors specifically selecting complete information, resulting in a 100% response rate for most studies (n = 28). However, it should be noted that minor instances of non-response may still exist within the original datasets.

### Exposure assignment in studies of household air pollution

Most (n = 27) of the studies related to household air pollution exposure. Eleven of the studies used the Demographic and Health Survey (DHS) data to assign proxies of air pollution indirectly [18,19,47,48,53–55,62,65,75,76]. The DHS is a multi-country representative survey that has provided essential data on population, health, and nutrition in more than 90 LMICs since the early 1980’s. It recruits participants, usually women aged 14–49 years, using multi-stage cluster sampling and asks about the type of fuel used for cooking and the place of cooking [77], and so serves as a proxy only for household PM exposure. Fuel options include “electricity, liquid petroleum gas (LPG)/biogas, kerosene, cool/ignite, charcoal, wood, agricultural crops/straw, shrubs/grass, animal dung, and no cooking” in the households. Additionally, DHS data on smoking habits were used in one study [62] to assess the impact of maternal smoking on infant and child mortality.

Fifteen of the eligible household studies, including DHS studies (n = 11), administered structured questionnaires through interviews to investigate nuanced aspects of cooking practices. Four studies [49,68,69,71] gathered data on the types of cooking fuel used in households. Three studies [52,72,78] specifically scrutinized the use of wood biofuels. Another five studies [50,64,67,69,70] probed into the nature of the kitchen or the location of the cooking area. One study [73] surveyed the practice of mothers carrying their children while engaged in cooking activities. The remaining twelve [50,52,56–60,62,70,72–74] studies conducted interviews to gather data on secondhand smoke exposure from parents and neighbors.

The type of fuel used for cooking was a key variable used as a proxy for household air pollution exposure in the majority of HH studies, with exposed groups defined by the use of: ‘solid fuels’ [19,20,48,62,66]; ‘biomass fuels’[63] (coal, lignite, charcoal, wood, straw, shrubs, grass, agricultural crop, animal dung); being ‘exposed’ (wood, charcoal, dung, crop residues, shrubs, coal, kerosene)[18]_;_ ‘firewood’[49]_;_ ‘mixed fuels’ (using both clean and solid fuels)[20]; ‘household pollutants’ (smoking, nature and location of kitchen)[50]; ‘kitchen smoke’[51]; ‘indoor cooking’ [67,69,70]; ‘kerosene’[19]; ‘gas stove cooking fuel’[71]; ‘unclean cooking fuels’ (biomass, firewood, charcoal, kerosene)[68]; ‘charcoal fuel’[63] or ‘other pollutant cooking fuels’ (coal, lignite, paraffin, kerosene)[63]_;_ ‘wood smoke’[72]_;_ ‘wood biofuels’[52] or ‘polluting fuels’ (wood, dung, coal, paraffin)[55]. The comparator or non-exposed groups were defined as using: ‘non-solid fuels’ (electricity, LPG, natural gas, biogas, kerosene) [47,48,62]_;_ ‘clean fuels’ (natural gas, biogas, LPG, electricity)[18–20,55,63,68]; having a ‘non-smoker in the house’ [52,57,58,62,70–74]; living in ‘low polluted areas (ambient PM_2.5_ levels < 25 µg/m³, measured with a DustTrak II Model 8532 air sampler)[66]; having ‘non-firewood fuels’[49]; ‘never carrying a child while cooking’[73]; ‘outdoor cooking’ [18,67,69,70]_;_ ‘non-gas stove cooking fuel’[71]; ‘non-biomass cooking fuels’[53]; ‘separate kitchen in the household’ [18,64]; ‘no exposure to wood smoke’ [51,72] or ‘no exposure to wood biofuels’[52]. Kerosene was categorized as both ‘polluting fuels’ [19,63,68] and ‘clean /non-solid’ fuels [20,47,48,62] across different studies Gas cooking was also classified as a “clean” cooking fuel [20] (Table 2 and S4 File).

#### Exposure assignment used in ambient air pollution studies.

For studies that measured or modelled ambient air pollution, one study [20] employed a pre-established land use regression model (LUR) developed for NO_2_ and NO_x_ which explained 68% and 75% of NOx and NO_2_ variances, respectively, and assigned it to the study participants’ residential addresses [79]. Another study [65] was conducted by matching the DHS enumeration area (the lowest geographic cluster in the DHS survey) with global satellite-estimated PM_2.5_ concentrations modeled by the Atmospheric Composition Analysis Group at Washington and Dalhousie University of the United States and Canada [80]. Three studies [59,61,66] used a hand-held sampler to collect real-time PM_2.5_ measurements at villages in the designated enumeration area (EA). The mean average PM_2.5_ within the assigned EAs was then used to examine the impact of PM_2.5_ on morbidity and mortality for children under five years (Table 2 and S4 File).

#### Potential confounders and modifiers.

Potential confounders adjusted for in the studies related to household, parental, child, and other environmental and contextual factors, and are presented in the supplementary file (S4 File). In our review, we found that none of the studies fully controlled for all key potential confounding factors like socioeconomic status, household condition, behavioural factors, and child nutritional condition, which can significantly influence air pollution's impact on child respiratory health, alongside factors like physical activity, season, and time trends. Additionally, four studies failed to report any adjustments for confounding [50,51,56,58]. In most studies (n = 15) that underwent confounder assessment, data collection on potential confounders, including birth size, relied on self-report questionnaires, although two studies [56,58] utilized health registration records for data acquisition. Five studies conducted a sensitivity analysis, showing a relatively smaller exposure-outcome association when potential confounders (e.g., season, breastfeeding, and birthweight) were excluded [18,20,62,65,67].

We observed that most studies reported differences in prevalence between exposed and unexposed groups in potential confounders such as maternal education, type of residence, birth size, breastfeeding status, and household factors but did not match or adjust for these differences in the analysis. For those studies that did adjust for confounders, we noted that this reduced the effect of air pollution on child respiratory hospitalization and mortality [19,59].

#### Summary of findings.

The findings of this systematic review indicated that the majority of studies have focused on proxies for air pollution, such as the use of solid fuels for cooking and household tobacco smoking, with limited emphasis on rigorous, objective assessments of both household and ambient air pollution exposures and their associated health impacts in SSA.

### All-cause mortality

Sixteen studies evaluated the effect of air pollution on all-cause mortality in children under five years (Table 2). 14 of these studies reported on household air pollutants (i.e., solid fuels used for cooking or light; tobacco smoking at home) and two studies reported on ambient PM_2.5_ concentrations as the exposure. Ten of the fourteen studies reported the effects of using solid fuels on all-cause mortality, with seven of these studies reporting a significant association between solid fuel use and risk of all-cause mortality [18,47,48,53,55,62,63]. Conversely, three studies [19,20,54] reported that solid fuel use was not significantly associated with all-cause under-five mortality. Three studies [18,53,63] specifically reported a statistically positive association between biomass fuel use and increased risk of all-cause mortality, while two studies reported a similar adverse association with charcoal fuel use [18,63].

Three studies [18,63,64] highlighted the critical role of kitchen location, indicating that in-house kitchens were associated with an increased risk of all-cause mortality in children under five years. However, one study [63] reported that statistically significant association (HR = 0.98; 95% CI: 0.95–1.01).

Three studies reported that indoor tobacco smoking significantly contributed to all-cause mortality in children under five years [56,63,67] and one study [62] observed that maternal smoking, in conjunction with the use of solid fuels in households, resulted in a 59% increased risk of infant mortality.

Two studies reported that ambient PM_2.5_, estimated through satellite-based data from the Washington Atmospheric Composition Analysis Group (ACAG) and measured with DustTrak II Model 8532 air samplers (TSI Inc.), was an independent predictor of all-cause mortality in children under five years of age [65^,^ 66], while one study reported that neither NOx nor NO_2_ were associated with neonatal mortality [20].

### Respiratory mortality

Three studies related to respiratory mortality [50,66,73] reported varying findings, with one reporting a five-fold increased odds of ALRI-related mortality in children who were always carried by their mother while cooking compared to those who never had [73], and an increased but non-significant risk associated with a higher composite index of indoor air pollution (scored based on whether the kitchen was inside the house, the type of fire or fuel used, if the mother carried the child while cooking, and parental smoking habits)[73], and outdoor PM_2.5_ measured with DustTrak II Model 8532 air samplers (TSI Inc.) [66]. While our analysis showed a significant association between solid fuel exposure and under-five mortality, the limited availability of cause-specific mortality data restricted a deeper analysis of other air pollution-related causes of death.

### Respiratory hospitalisations

Respiratory hospitalization outcomes reported in eligible studies included pneumonia (n = 14 studies), wheezing illness(n = 1 study), ARI (n = 4 studies), asthma (n = 1 studies), bronchitis (n = 2 studies), and pulmonary tuberculosis (n = 1 studies) (Table 2). A total of eight studies observed the relationship between parental tobacco smoking and pneumonia in children under five years, with four studies [52,57,59,70] reporting statistically significantly adverse association, while the other four studies [61,68,69,74] showed an increased but statistically insignificant association. Additionally, three studies [49,50,72] reported a significant adverse association between exposure to household biomass fuels and ARI, while one study did not [52].

Three studies [49,50,52] observed no statistically significant association between passive smoking and the odds of ARI in children under five, whereas one study [72] reported a significant association. One study [71] found no significant association between comorbidity with asthma and bronchitis and the use of gas stoves for cooking (OR = 1.4; 95% CI: 0.5–4.5), although the likelihood of asthma was three times higher in children from households using gas stoves (OR = 3.8; 95% CI: 1.2–13.3) compared to those from households without gas stoves. Our review showed variability in the association between parental smoking and bronchitis, with one study [71] reporting an increased, but non-significant association (OR = 1.5; 95% CI: 0.8–2.7), and another [52] reporting a significant protective association between passive smoking and bronchitis (RR = 0.35; 95% CI: 0.13–0.99).

**Fig 2:**
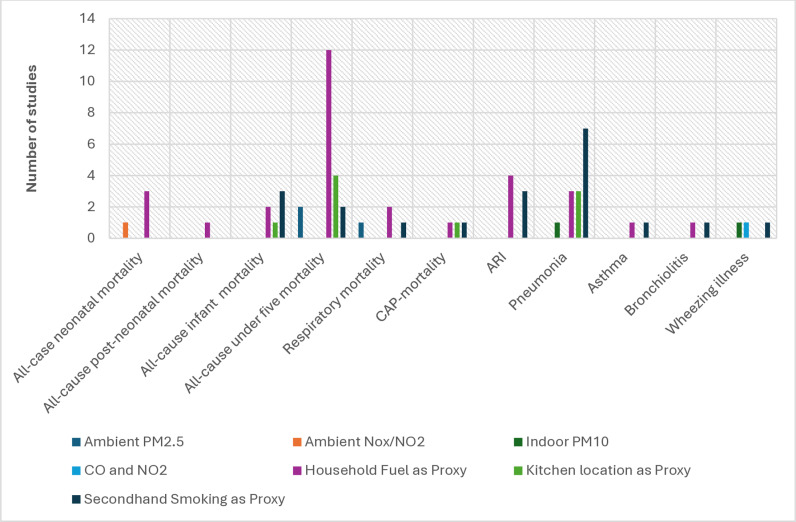
Distribution of mortality and respiratory hospitalizations by exposure type.

Fig 2 depicts the type and number of outcomes reported in the 31 studies by exposure type. The number of outcomes reported in each study ranged from 1 to 12, with all-cause under-five mortality associated with household fuel use (n = 12) and pneumonia associated with second-hand smoking (n = 7) being the most reported outcomes.

### Geographic disparity

The result of the systematic review showed the impact of air pollution exposure showed significant geographical disparities across SSA where indoor air pollution from solid fuel use in West Africa was a predominant factor, increasing the risk for under-five mortality. In Nigeria, two studies [47,54] reported that solid fuels were associated with high neonatal and child mortality rates. Specifically, 82% of neonatal deaths and 90% of post-neonatal deaths occurred in households where solid fuel use was prevalent, particularly in rural areas. These findings were further corroborated by Imo et al.,[48], where household solid fuel use was associated with over a two-fold increase in the risk for U5M.

However, studies in East Africa have identified a dual burden of indoor and outdoor pollution. In Kenya, exposure to ambient PM_2.5_, exceeding 25 µg/m³, elevated the risk of all-cause mortality by 22%[66], while in Ethiopia, each 10 µg/m³ increase in ambient PM_2.5_ corresponded to a 129% increase in U5M risk [65]. Indoor cooking with solid fuels exacerbated the risk of mortality, as demonstrated in a study in Kenya reported a 91% increased risk of U5M associated with indoor cooking using solid fuel [67], and in Ethiopia, where the absence of separate kitchen facilities increased infant mortality risk by 77% [64].

Two studies in South Africa reported households utilizing polluting fuels such as wood, coal, and paraffin face a nearly two-fold increase in U5M risk, with tobacco smoke further exacerbating the risk of sudden child death [55,58]. A paucity of studies in Central Africa mirrored the trends observed in other regions of SSA, indicating the significance of household energy sources across SSA in influencing mortality outcomes. Regionally, cause-specific mortality further revealed the variability in the impact of air pollution. Exposure to outdoor PM_2.5_ in East Africa [66] significantly associated with respiratory mortality, while in West Africa [50,51,73], indoor pollution from wood smoke and tobacco was implicated in ALRI and pneumonia-related deaths, with studies from the Gambia [73] and Nigeria [50^,^ 51] demonstrating the significant contribution of exposure to wood smoke and maternal tobacco smoking on child respiratory mortality.

The result of systematic review further demonstrated that exposure to household air pollution from solid fuels and tobacco smoke in East Africa was a significant risk factor for respiratory infections in children under five years of age. In Uganda, exposure to tobacco smoke increased the risk of pneumonia threefold [70], although outdoor cooking was not significantly associated with pneumonia risk. Similarly, in Tanzania, unclean cooking fuels from firewood and charcoal fuels were significantly associated with severe pneumonia, whereas passive smoking showed no significant association [68]. A study in Uganda [71] also demonstrated the role of gas cooking, which increased asthma risk, further emphasizing that household energy sources contributed significantly to childhood respiratory morbidity. The inconsistent association between tobacco smoking and ARI outcomes in some East African studies underscored the importance of both traditional cooking practices and modern fuels in shaping respiratory health outcomes.

In Central Africa, particularly in Cameroon, wood smoke exposure was significantly linked to acute respiratory infections [72], with passive tobacco smoke being a major contributor to hospitalization due to respiratory conditions (OR = 3.58). In South Africa, studies have reported significant risks posed by both outdoor and indoor air pollution from cooking fuel and tobacco smoke exposure. Tobacco smoke consistently elevated the risk of ARI, pneumonia, and bronchitis across studies in South Africa, with maternal smoking playing a particularly prominent role [57,59,60]. Roux et al.,[60] found maternal smoking was associated with an increased risk of pneumonia in infants, while exposure to indoor PM_10_ and tobacco smoke heightened the risk of pneumonia hospitalization [61]. Toluene was also linked to an elevated risk of LRTIs [59].

Studies in West Africa have reported hazards of indoor air pollution from traditional cooking fuels, such as firewood and kerosene, and maternal tobacco risks. In Nigeria, three studies [49–51] identified the use of firewood and latrine as significant predictors of ARIs, with additional evidence linking maternal smoking to under-five pneumonia in a 30-month prospective study [51]. However, a study in Niger [74] reported no significant association between passive smoking and respiratory pathogen carriage, indicating regional variations in risk factors.

The results of the systematic review showed diverse, region-specific air pollution-related health risks in children under five years in SSA. Studies from West Africa indicated a primary impact of indoor air pollution due to solid fuels, while East African studies reported significant risks from both indoor and outdoor pollutants (proxies thereof). In South Africa, maternal tobacco smoke was the primary concern, with Central Africa facing mirrored risks from biomass and tobacco smoke exposure.

### Urban-rural distinction

The finding of two studies showed that, in rural areas, reliance on solid fuels (wood, charcoal, and biomass) increases exposure to indoor air pollutants. A study in Nigeria found that rural households use solid fuels three times more than urban households [54]. Another study [63] conducted using DHS data from 23 Sub-Saharan African countries found that 91% of rural children were exposed to biomass smoke, with an increased under-five mortality risk (HR 1.21 for charcoal, 1.20 for other biomass fuels).

Urban-based study in Nairobi, Kenya [66] found exposure to outdoor PM_2.5_ raised children’s all-cause mortality risk by 22%, while in Adama, Ethiopia [20], a 10-unit increase in ambient NO_2_ was linked to a non-significantly higher neonatal mortality risk (OR = 0.91; 95% CI: 0.32–2.58). In a study in Ibadan Nigeria [51,81], indoor cooking with wood and charcoal significantly increased ARIs and pneumonia in children under five. In South Africa, maternal tobacco smoking in urban areas exacerbates these risks, with studies from Cape Town [58] and Johannesburg [57] linking maternal smoking to higher rates of sudden infant death and a fivefold increased risk of bacterial pneumonia in children.

In summary, this systematic review found significant adverse associations in 16 of 25 (64%) cross-sectional studies of exposure to solid cooking fuels and all-cause mortality, and 2 of 5 (40%) cross-sectional studies of exposure to second-hand/passive tobacco smoke during cooking and respiratory-related mortality. Additionally, 13 out of 31 studies (5 cross-sectional, 5 case-control, and 3 cohort) reported significant adverse associations between exposure to household cooking fuels (n = 8), household parental tobacco smoking (n = 6), indoor PM_10_ (n = 2), and an increased risk of respiratory hospitalizations in children aged under-five. However, seven (33.3%) findings were negative or reported non-significant associations.

### Meta-analyses

**Fig 3:**
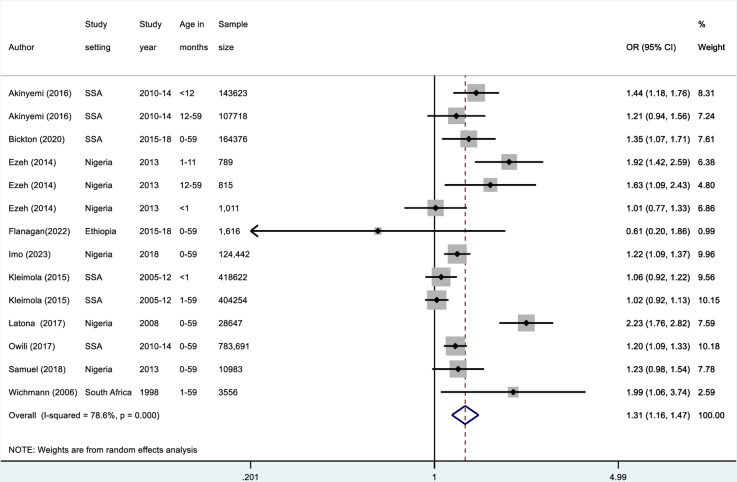
Forest plot of meta-analysed associations between exposure to solid fuel smoke and all-cause under-five mortality.

We conducted a meta-analysis of fourteen findings from ten studies [18–20,47,48,53–55,62,63] to estimate a pooled OR for all-cause mortality in children under five years exposed to solid fuels in the home. In Fig 3, the forest plot and the pooled result from the random effect model illustrated that children exposed to solid fuel had a 1.31 times higher odds of all-cause mortality compared to those non-exposed to solid fuels (OR = 1.31; 95% CI: 1.16–1.47; τ² = 0.0319; *X*^2^ = 60,7%, df = 13(P < 0.0001); I^2^ = 78.6. The I^2^ statistic indicated considerable heterogeneity between the studies, although the random effects meta-analytic statistic was significantly different from 1 (P <  0.0001), indicating a high confidence in the finding.

**Fig 4:**
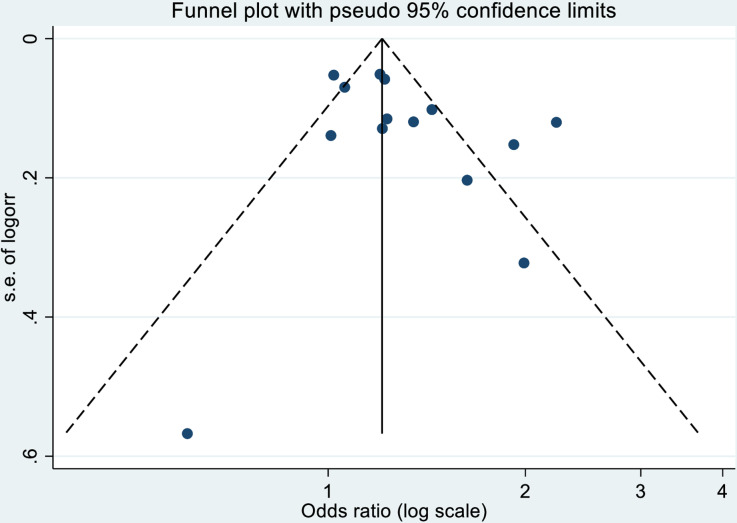
Funnel plot of the meta-analyzed association between exposure to solid fuel smoke and all-cause under-five mortality (X-axis -logarithmic scale of OR (logOR) Y-axis-standard error of logOR.

The funnel plot in Fig 4 indicated some asymmetry, with more studies on the right-hand side of the center (the pooled OR estimate) than on the left-hand side and very few studies at the bottom of the funnel, suggesting potential publication bias with perhaps a lack of publication of smaller studies. The contour-enhanced funnel plot (supplementary file , S6) indicates perceived missed studies in the bottom two-thirds (apart from one study down at the bottom) and to the left of the plot, containing regions of high and low statistical significance, suggesting that funnel asymmetry cannot be solely attributed to publication bias. The regression-based Egger test with REML estimation to address residual heterogeneity indicated that the slope coefficient was not significantly different from zero (p = 0.69), and the bias coefficient was also not statistically significant (p = 0.11). The overall p-value for the test of the null hypothesis (no small-study effects) was 0.11, suggesting no significant evidence of small-study effects. This implies that the observed heterogeneity cannot be concluded to arise from small-study effects.

To further explore the observed heterogeneity, we conducted three sub-group analyses: by study year, study setting (single-country vs multi-country study), and by geographic region (Supplementary file, S6). The first subgroup analysis to examine the potential impact of the introduction of the Sustainable Development Goals (SDGs) in 2015 showed that the estimated pooled OR for studies conducted before 2015 was 1.34 (95% CI: 1.16–1.54; I^2^ = 82.9; p < 0.0001), and for those during and after 2015 was 1.24 (95% CI: 1.10–1.39; I^2^ = 78,6%, p = 0.343). The subgroup analysis by study setting found the estimated pooled OR for multi-country studies to be 1.18 (95% CI:1.06–1.31; I^2^ = 64.4%, p = 0.015) and for single-country studies was 1.46 (95% CI; 1.16–1.83; I^2^ = 80.0%, p < 0.0001). The sub-group analysis based on geographic region showed the pooled OR for West Africa as 1.47 (95% CI: 1.16–1.86; I² =  84.2%, p <  0.000), for East Africa as 0.61 (95% CI: 0.20–1.85; p <  0.000), and for South Africa as 1.99 (95% CI: 1.04–3.68; p <  0.000). The I^2^ test result revealed there was still significant heterogeneity in all subgroup analyses. In our sensitivity analysis based on study quality, excluding one study with a “high risk” of confounding bias did not significantly alter the heterogeneity, which remained high (OR = 1.29, 95% CI: 1.15–1.45; I² = 79.4%, p < 0.0001) (Supplementary file, S6). The leave-one-out sensitivity analysis to explore the influence of a single study on the overall effect size estimate showed the 95% CI of each omitted study contained the overall meta-analytic effect size estimated using all studies (OR = 1.31) (Supplementary file, S6 File). Hence, the source of the between-study heterogeneity remains unexplained based on the observed data.

**Fig 5:**
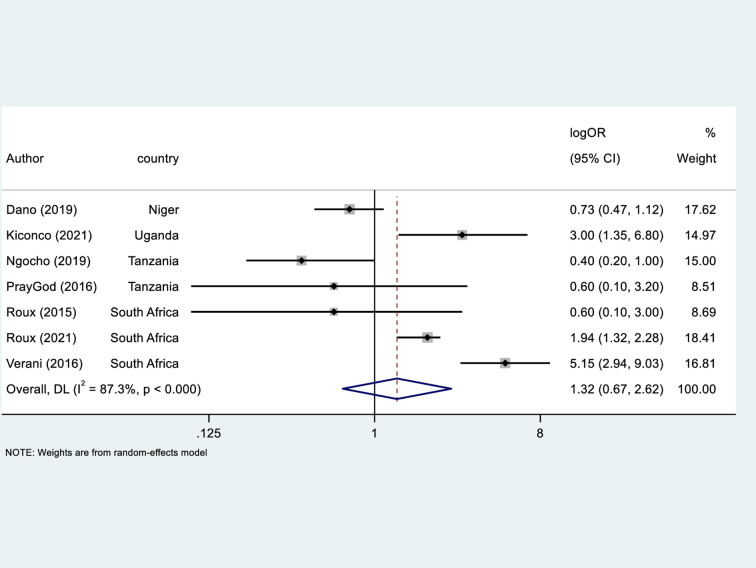
Forest plot of meta-analysed associations between exposure to passive smoking and pneumonia in children under-five years.

Our second meta-analysis, drawing from seven studies, investigated the association between exposure to indoor tobacco smoke and the odds of pneumonia in children aged under five years [57,68–70,74], for which we report an increased, but non-significant, odds ratio (OR) of 1.32 (95% CI; 0.67–2.62) (Fig 5). The I2 statistic indicated that there was high heterogeneity between the studies (I^2^ = 87.3%, P < 0.0001). The wide confidence interval and high heterogeneity mean there is substantial variability between the results of the seven original studies. A funnel plot was not produced in this instance because of the minimal number (<10) of studies [82].

## Discussion

This systematic review provides evidence on the relationship between exposure to sources of household air pollution (or proxies thereof) and both mortality and hospitalization for respiratory illness among children aged under five years living in SSA. Our review found that there was a dearth of studies that used measured air pollution measurements to assign exposure. The two studies [65^,^ 66] that reported exposure to ambient PM_2.5_, reported that higher levels of ambient PM_2.5_ were associated with an increased risk of all-cause mortality in children under five years. These were consistent with a cross-country panel review of sixteen Asian countries conducted by Anwar et al.[83] which concluded that each one µg/m^3^ increase in annual PM_2.5_ resulted in a 14.5% increase in the number of children dying before the age of five.

The systematic review indicated that exposure to solid fuel has a strong, significant increased risk of under-five mortality, especially when combined with parental smoking. Suryadhi et al.[84] supported this finding, noting a synergistic effect of combined exposure that warrants further consideration in public health interventions. However, as this conclusion stems from a single study, it may be subject to overestimation due to small sample size and quality issues.

The meta-analysis showed that exposure to solid fuels in the home, increased the odds of all-cause mortality in children aged under five years. This finding is in agreement with the recent meta-analysis by Lee et al.[85] who observed an increased risk of under-five years mortality in children exposed to polluting fuels used for household cooking or heating (RR = 1.25 (95% CI: 1.18–1.33)) compared with our study’s OR of 1.31 (95% CI: 1.16–1.47). The subgroup analysis by study period, pre- and post-2015, observed continued impact of solid fuel use in studies conducted in post-2015, the era of SDGs, with an increased odds of mortality of 24%, suggesting air pollution from indoor household sources remains a fundamental pollution source to control to prevent childhood mortality.

In our systematic review, only four studies assessed the relationship between HAP from cooking fuels (n = 3) and ambient PM_2.5_ (n = 1) with ARI and CAP-related mortality in children under five years of age in SSA [50,51,66,73]. These studies showed a significant association between household air pollution from cooking fuels and ambient PM_2.5_, and ARI and/or CAP-related mortality. This finding was in line with a recent modeling study by Frosta et al.[86], who observed that 49.8% of respiratory mortality among children under five years in SSA in 2018 was attributable to total exposure to ambient and indoor PM_2.5_. The systematic review indicated that consistently carrying a child while cooking increased the odds of ARI mortality in children under five by fivefold, likely due to greater exposure to harmful pollutants [73]. However, this finding is from a single study and may be confounded by uncontrolled factors like fuel type and ventilation. It could also be influenced by small sample size, raising concerns about overestimation. While this highlights a potential intervention area, caution is needed to avoid unintended harm, such as neglect or increased injury risk if discouraging child-carrying during cooking.

Our systematic review found an inconclusive association between indoor air pollution exposure (mainly measured using proxies such as passive smoking and cooking fuel type) and hospitalisations for ARI, pneumonia, bronchitis, and asthma. This uncertainty may arise from poorly measured exposure or incomplete adjustment for confounders. A similar review has shown that reliance on proxies can obscure true relationships [25]. The variation in epidemiological findings might be attributable to population characteristics, cultural differences, and the type of fuels used in a wide range of LMICs.

Our meta-analysis of seven studies of children exposed to second-hand tobacco smoke indicated they were underpowered and did not allow a definite conclusion to be drawn. A previous meta-analysis by Riestiyowati et al.[87] using data from 12 primary studies from four continents (Africa (Tanzania, Ethiopia, and Morocco), South America (Brazil), Asia (Indonesia, Bangladesh, Nepal, and Vietnam) and Australia), reported results from two separate analyses which showed a 66% and about a twofold increase in odds of pneumonia in under-five years children exposed to second-hand smoke.

The potential mechanisms for adverse effects of air pollution on children’s health have been proposed in recent studies [88–90]. It has been widely suggested that children’s physiological immaturity makes them inherently vulnerable to the adverse effects of air pollution. In addition, there are many demographic and behavioural factors that influence the impact of air pollution on children, such as this age group being more likely to spend much of their time inside homes [91^,^ 92].

The strengths of this systematic review include the focus on young children living in SSA and the inclusion of both household and ambient air pollution as exposures of interest, in an effort to study the impacts of total air pollution exposure. We employed a systematic, rigorous, and replicable approach for the review, including a comprehensive search strategy across a number of databases, retrieving published and preprinted articles to be able to incorporate the most recent articles. The quality appraisal process adhered to best practice guidelines tailored specifically for environmental health research and included screening by two reviewers throughout the whole process to minimize reviewer bias. The majority of studies used population-based large datasets such as DHS data, which serves as the primary and sometimes only source of essential data in Africa and other LMICs [26], allowing analysis with a large sample size and complete data.

However, there are limitations to our systematic review and meta-analysis. Our search focused on peer-reviewed articles in English, limiting inclusivity of articles published in other languages. Although efforts were made to encompass all studies from SSA countries, included studies were limited to eight countries, cautioning against broad extrapolation of results to all of SSA.

Exposure misclassification is likely to have occurred in some studies. For instance, kerosene fuel was inconsistently classified variably as ‘clean’ and ‘polluted’ [20,63,68], potentially leading to misclassification of exposure [93]. Furthermore, a major limitation is the reliance on proxy exposure measures, particularly from household surveys and indirect data. Relying only on information on fuels used for cooking and/or location of the kitchen within the property are relatively crude measures of exposure and do not account for time spent cooking, or nature and frequency of the biomass emissions. This introduces uncertainty when interpreting the findings and highlights the necessity for future research to use more precise exposure assessment methods. Consequently, our understanding of the causal association between household air pollution and health outcomes remains limited.

Methodological considerations including measurement bias, recall bias, and confounding, which all warrant consideration when interpreting findings. Importantly, low socioeconomic status (SES) can significantly confound the relationship between air pollution exposure and health outcomes. However, most studies in our review are cross-sectional and often inadequately adjusted for SES. This variability in study quality can lead to misleading associations. Studies that appropriately considered SES confounders demonstrated more robust associations between air pollution and adverse health effects [59,61]^.^.

Another potential limitation relates to the assignment of hospitalization outcomes, which necessitated a broad search approach without specific disease codes. In addition, between-study heterogeneity was detected in our meta-analysis and was not reduced in both subgroup analyses and sensitivity analyses based on study quality, with inherent differences between the studies in aspects such as population structure and study designs. However, our leave-one-out sensitivity analysis showed the robustness of our estimation. Future studies could benefit from conducting an Individual Participant Data (IPD) meta-analysis using DHS data to enable granular analyses and explore detailed geographical disparities

The findings of this review underscore the critical need for effective public health strategies to mitigate the harmful effect of air pollution on young children living in SSA. However, in order to develop such strategies it is vital that better designed studies are conducted to provide an evidence base for actions. Significant gaps remain in understanding causation due to the nature of the studies in our systematic review, meaning that causality cannot be implied. To better understand the impact of air pollution on health outcomes, particularly in vulnerable populations such as children under five years, there is an urgent need for studies that incorporate objective measurements of air pollution for both indoor and outdoor environments. Such studies will provide more accurate and reliable data, enabling us to identify areas where interventions could be targeted most effectively.

PM_2.5_ is a mixture of various pollutants, and the chemical composition and source of particles may have different toxicities and health effects among children [94], underscoring the need to better determine the impacts of differing sources of air pollution by improved measurement of not only total pollution concentrations but also source specific contributions. Therefore, more studies are needed using valid and reliable measurement techniques to estimate exposure levels more accurately and so to minimise exposure misclassification or bias.

Further investigation is needed on the effect of solid fuel use on under-five years mortality and morbidity, including behavioural, and contextual drivers of use of fuel. Although some evidence exists for the adverse effects of solid fuel use, these may not capture the diversity and complexity of fuel sources and types in SSA, and findings from one country or region may not be applicable to another due to differences in type, availability, and affordability of cooking fuels as well as behavioural factors [63,85]. Further, the effect of demographic and other contextual variables such as socioeconomic conditions, nutrition, immunization remains unclear in SSA [62,67]. These factors may confound or exacerbate the relationship between air pollution and child health and may vary across different regions and populations in SSA.

This review found that most studies were cross-sectional in design, which can only show the association between air pollution and child health at a given point in time and may not capture the cumulative and chronic effect of air pollution exposure [85,95]. Therefore, better designed studies are needed, such as randomised trials or cohort studies, that can better assess the causal relationship between air pollution and adverse child health outcomes.

The effect of air pollution on child health depends on the location and movement or activity of the child, such as whether they are indoors or outdoors, near or far from the source, or engaged in different levels of activity. Simultaneous measurement of indoor and outdoor air is rarely performed in studies from SSA [11]. The region lacks evidence on the effect of the total burden of indoor and ambient air pollution on under-five years mortality. Therefore, studies are needed to assess the interactions and synergies between indoor and outdoor sources and pollutants, so to be able to develop and implement policies and regulations that address all sources of air pollution, especially given exposure is affected by factors beyond the household or individual level, such as community emissions, industrial emissions, traffic, or agricultural sources.

## Conclusion

In summary, our meta-analysis found that the use of solid fuel in homes was associated with an increased risk of mortality in children under five years in SSA. However, a second meta-analysis provided insufficient evidence to establish a significant association between second-hand smoke and an increased risk of hospitalization due to pneumonia in children under five years of age. While there were very few studies that investigated the effects of ambient air pollution, and so we were unable to meta-analyse the results, the two eligible studies reported associations between increases in ambient PM_2.5_ and risk of all-cause mortality. We recommend that future studies conducted in SSA address previous study flaws to ensure that: 1) both indoor and ambient air pollution exposures are more accurately assessed, preferably by collecting measurements; 2) that potential confounders are accounted for in the statistical analyses; and, 3) that stronger epidemiological study designs better suited to infer causality, such as randomised trials of interventions taking into account total exposure, or cohort studies, are conducted.

## Supporting information

S1 File PRISMA-2020 Checklist. (DOCX)

S2 File Search strategy. (DOCX)

S3 File Irrelevant studies which were excluded during the title and abstract screening process (N=5304). (CSV)

S4 File Studies screened for full-text review and data extraction. (DOCX)

S5 File Risk of bias assessment. (DOCX)

S6 File Meta-analysis. (DOCX)

S7 File Certainty of evidence assessment. (DOCX)

## Abbreviations

DHS: Demographic and Health Survey
OHAT: Office of Health Assessment and Translation
NIEHS-NTP: National Institute of Environmental Health Science-National Toxicology Program
WHO: World Health Organization.
