# Supplementary material for: The effect of air pollution on morbidity and mortality among children aged under five in sub-Saharan Africa: Systematic review and meta-analysis
Source: PLoS One. 2025 Apr 10;20(4):e0320048. doi: 10.1371/journal.pone.0320048 (PMC11984980; doi:10.1371/journal.pone.0320048)
Supplement: S2 File — (DOCX) [file pone.0320048.s002.docx]

## **Supplementary file S2:** Search strategy

**Supplementary table 1 S2: EMBASE**

| 1 | exp air pollutant/ or environmental exposure/ or exp air pollution/ or exp particulate matter/ or Air pollution*.mp. | 350914 |
| --- | --- | --- |
| 2 | air quality.mp. or exp air quality/ | 47352 |
| 3 | indoor air pollution.mp. or exhaust gas/ or exp indoor air pollution/ or exp combustion/ or "particulate matter 2.5"/ | 70963 |
| 4 | carbon dioxide/ or ambient air/ or indoor air quality.mp. | 179484 |
| 5 | outdoor air pollution.mp. or exp asthma/ or exp nitrogen dioxide/ | 340860 |
| 6 | carbon monoxide/ or outdoor air quality.mp. | 47736 |
| 7 | ambient air pollution.mp. | 3895 |
| 8 | sulfur dioxide/ or ambient air quality.mp. or ozone/ | 51343 |
| 9 | air pollutant*.mp. | 48676 |
| 10 | exp cooking/ or exp biomass/ or household air pollution.mp. | 112165 |
| 11 | household air quality.mp. | 31 |
| 12 | traffic related air pollution.mp. or exp traffic/ | 174014 |
| 13 | particulate matter.mp. | 78688 |
| 14 | exp particulate organic matter/ or "particulate matter 1.0"/ or ultrafine particulate matter/ or suspended particulate matter/ or "particulate matter 2.5"/ or exp atmospheric particulate matter/ or particulate*.mp. or exp particulate matter exposure/ | 120549 |
| 15 | PM10.mp. | 14623 |
| 16 | "PM2.5".mp. | 26293 |
| 17 | carbon monoxide.mp. | 64897 |
| 18 | CO.mp. | 3048355 |
| 19 | Sulfur dioxide.mp. | 20213 |
| 20 | ozone/ or SO2.mp. | 52670 |
| 21 | nitrogen dioxide.mp. | 20278 |
| 22 | nitric oxide/ or NO2.mp. | 209005 |
| 23 | nitrous oxide/ or nitric oxide*.mp. or nitrogen oxide*.mp. or nitrous oxide*.mp. | 356672 |
| 24 | "NO".mp. | 7043682 |
| 25 | ozone.mp. or ozone depletion/ | 44919 |
| 26 | O3.mp. | 20363 |
| 27 | biomass.mp. | 136813 |
| 28 | biofuel production/ or exp biofuel/ or biofuel*.mp. | 32261 |
| 29 | exp methane emission/ or exp greenhouse gas emission/ or emission*.mp. or exp nitrous oxide emission/ | 569373 |
| 30 | exhaust gas/ or combustion/ or coal/ or exp internal combustion engine/ or combustion.mp. | 72742 |
| 31 | smoke.mp. | 91706 |
| 32 | smog/ or smog.mp. | 4434 |
| 33 | gaseous pollutant*.mp. | 1660 |
| 34 | particle pollution.mp. | 296 |
| 35 | 1 or 2 or 3 or 4 or 5 or 6 or 7 or 8 or 9 or 10 or 11 or 12 or 13 or 14 or 15 or 16 or 17 or 18 or 19 or 20 or 21 or 22 or 23 or 25 or 26 or 27 or 28 or 29 or 30 or 31 or 32 or 33 or 34 | 4997769 |
| 36 | child/ or hospitalized child/ or exp infant/ or preschool child/ or toddler/ | 3380990 |
| 37 | under-five child*.mp. | 2108 |
| 38 | baby.mp. or baby/ | 88372 |
| 39 | infant.mp. or infant disease/ or infant mortality/ or hospitalized infant/ | 976224 |
| 40 | child death/ or newborn death/ | 20768 |
| 41 | child health/ or child*.mp. or child health care/ or child hospitalization/ | 3534093 |
| 42 | newborn disease/ or neonatal pneumonia/ or neonatal pneumothorax/ | 37180 |
| 43 | newborn morbidity/ or newborn*.mp. or newborn/ or newborn death/ or newborn mortality/ | 850842 |
| 44 | (neonate* or neonatal).mp. | 441099 |
| 45 | "Pediatric Index of Mortality"/ or pediatric patient/ or Pediatric*.mp. or Paediatric*.mp. | 843950 |
| 46 | Paediatric.mp. | 133562 |
| 47 | 36 or 37 or 38 or 39 or 40 or 41 or 42 or 43 or 44 or 45 | 4598600 |
| 48 | morbidity/ or perinatal morbidity/ | 430738 |
| 49 | Morbidity.mp. | 839101 |
| 50 | mortality.mp. or all cause mortality/ or childhood mortality/ or mortality/ | 1992215 |
| 51 | death/ or "cause of death"/ or dying/ or perinatal death/ | 484797 |
| 52 | death.mp. | 1663632 |
| 53 | (respiratory tract infection* or respiratory infection* or respiratory disease* or respiratory tract disease*).mp. | 275451 |
| 54 | exp pneumonia/ or pneumonia*.mp. | 588435 |
| 55 | asthma.mp. | 359449 |
| 56 | (hospitalization* or hospitalisation*).mp. or hospital admission/ | 910982 |
| 57 | coughing/ or URTI.mp. or exp respiratory tract infection/ | 688741 |
| 58 | LRTI.mp. | 2743 |
| 59 | 48 or 49 or 50 or 51 or 52 or 53 or 54 or 55 or 56 or 57 or 58 | 5250598 |
| 60 | 35 and 47 and 59 | 225919 |
| 61 | Sub-Saharan Africa.mp. or exp "Africa south of the Sahara"/ | 341014 |
| 62 | 60 and 61 | 4953 |
| 63 | limit 62 to (human and english language and (infant <to one year> or child <unspecified age> or preschool child <1 to 6 years>)) | 3185 |

**Supplementary table 2 S2: PUBMED**

| SNO | Search statement | Result |
| --- | --- | --- |
|  | "Air Pollution"[MeSH Terms] OR "Particulate Matter"[MeSH Terms] OR "Air Pollutants"[MeSH Terms] OR "Traffic-Related Pollution"[MeSH Terms] OR "Sulfur Dioxide"[MeSH Terms] OR "Carbon Monoxide"[MeSH Terms] OR "Carbon Monoxide Poisoning"[MeSH Terms] OR "Nitrogen Dioxide"[MeSH Terms] OR "Nitrites"[MeSH Terms] OR "Nitric Oxide"[MeSH Terms] OR "Nitrogen Oxides"[MeSH Terms] OR "Ozone"[MeSH Terms:noexp] OR "Ozone Depletion"[MeSH Terms] OR "Biomass"[MeSH Terms] OR "Biofuels"[MeSH Terms] OR "Vehicle Emissions"[MeSH Terms] OR "Hydrogen Cyanide"[MeSH Terms] OR "Fires"[MeSH Terms] OR "Charcoal"[MeSH Terms] | 431,621 |
|  | ((((((((((((((((((((((((((((((((("air pollution*"[Text Word]) OR ("air quality"[Text Word])) OR ("indoor air pollution"[Text Word])) OR ("indoor air quality"[Text Word])) OR ("outdoor air pollution"[Text Word])) OR ("outdoor air quality"[Text Word])) OR ("ambient air pollution"[Text Word])) OR ("ambient air quality"[Text Word])) OR ("air pollutant*"[Text Word])) OR ("household air pollution"[Text Word])) OR ("household air quality"[Text Word])) OR ("traffic related air pollution*"[Text Word])) OR ("particulate matter"[Text Word])) OR (particulate*[Text Word])) OR (PM2.5[Text Word])) OR (PM10[Text Word])) OR ("carbon monoxide"[Text Word])) OR (CO[Text Word])) OR ("Sulfur dioxide"[Text Word])) OR (SO2[Text Word])) OR ("nitrogen dioxide"[Text Word])) OR (NO2[Text Word])) OR ("nitric oxide*"[Text Word])) OR ("nitrous oxide*"[Text Word])) OR (ozone[Text Word])) OR (O3[Text Word])) OR (biomass[Text Word])) OR (biofuel*[Text Word])) OR (emission*[Text Word])) OR (combustion[Text Word])) OR (smoke[Text Word])) OR (smog[Text Word])) OR ("gaseous pollutant*"[Text Word])) OR ("particle pollution*"[Text Word]) | 1,681,926 |
|  | (("schools, nursery"[MeSH Terms] OR "infant, newborn"[MeSH Terms:noexp] OR "Infant"[MeSH Terms:noexp] OR "Infant Death"[MeSH Terms]) AND "Infant Mortality"[MeSH Terms]) OR "Infant Health"[MeSH Terms] OR "Child Health"[MeSH Terms] OR "child, preschool"[MeSH Terms] OR "child, hospitalized"[MeSH Terms] OR "Child Mortality"[MeSH Terms] OR "infant, newborn, diseases"[MeSH Terms:noexp] | 1,060,812 |
|  | (("under five child*"[Text Word] OR "baby"[Text Word] OR "young child*"[Text Word] OR "infant"[Text Word] OR "child*"[Text Word] OR "newborn*"[Text Word] OR "neonate*"[Text Word] OR "neonatal"[Text Word]) OR (paediatric*[Text Word])) OR (pediatric*[Text Word]) | 3,634,291 |
|  | "Morbidity"[MeSH Terms:noexp] OR "Mortality"[MeSH Terms:noexp] OR "Hospital Mortality"[MeSH Terms] OR "Death"[MeSH Terms:noexp] OR "Respiration Disorders"[MeSH Terms] OR "Respiratory Tract Infections"[MeSH Terms] OR "Respiratory Tract Diseases"[MeSH Terms] OR "Pneumonia"[MeSH Terms] OR "Asthma"[MeSH Terms] OR "Hospitalization"[MeSH Terms] | 2,103,905 |
|  | ("morbidity"[Text Word] OR "mortality"[Text Word] OR "death"[Text Word] OR "respiratory tract infection*"[Text Word] OR "respiratory infection*"[Text Word] OR "respiratory tract disease*"[Text Word] OR "respiratory disease*"[Text Word] OR "pneumonia*"[Text Word] OR "asthma"[Text Word] OR "hospitalization*"[Text Word] OR "URTI"[Text Word] OR "LRTI"[Text Word]) OR (hospitalisation*[Text Word]) | 2,984,962 |
|  | "Africa South of the Sahara"[MeSH Terms] | 261,315 |
|  | "Sub Saharan Africa"[Text Word] OR "SSA"[Text Word] OR "Angola"[Text Word] OR "Benin"[Text Word] OR "Botswana"[Text Word] OR "Burkina Faso"[Text Word] OR "Burundi"[Text Word] OR "Cabo Verde"[Text Word] OR "Cameroon"[Text Word] OR "Central African Republic"[Text Word] OR "Chad"[Text Word] OR "Comoros"[Text Word] OR "Congo"[Text Word] OR "Cote d'Ivoire"[Text Word] OR "Equatorial Guinea"[Text Word] OR "Eritrea"[Text Word] OR "Ethiopia"[Text Word] OR "Gabon"[Text Word] OR "Gambia"[Text Word] OR "Ghana"[Text Word] OR "Guinea"[Text Word] OR "guinea bissau"[Text Word] OR "Kenya"[Text Word] OR "Lesotho"[Text Word] OR "Liberia"[Text Word] OR "Madagascar"[Text Word] OR "Malawi"[Text Word] OR "Mali"[Text Word] OR "Mauritania"[Text Word] OR "Mauritius"[Text Word] OR "Mozambique"[Text Word] OR "Namibia"[Text Word] OR "Niger"[Text Word] OR "Nigeria"[Text Word] OR "Rwanda"[Text Word] OR "Senegal"[Text Word] OR "Sierra Leone"[Text Word] OR "Somalia"[Text Word] OR "South Africa"[Text Word] OR "South Sudan"[Text Word] OR "Sudan"[Text Word] OR "Tanzania"[Text Word] OR "Togo"[Text Word] OR "Uganda"[Text Word] OR "Zambia"[Text Word] OR "Zimbabwe"[Text Word] | 531,494 |
|  | #1 OR #2 | 1,758,556 |
|  | #3 OR #4 | 3,634,291 |
|  | #5 OR #6 | 4,226,318 |
|  | #7 OR #8 | 542,195 |
|  | #9 AND #10 AND #11 AND #12 | 1,529 |
|  | #9 AND #10 AND #11 AND #12 AND ((humans[Filter]) AND (english[Filter]) AND (allchild[Filter] OR newborn[Filter] OR allinfant[Filter] OR infant[Filter] OR preschoolchild[Filter])) | 1,166 |

**Supplementary table 3 S2: Scopus**

|  | search stament | Result |
| --- | --- | --- |
|  | TITLE-ABS-KEY ( "air pollution*"  OR  "air quality"  OR  "indoor air pollution"  OR  "indoor air quality"  OR  "outdoor air pollution"  OR  "outdoor air quality"  OR  "ambient air pollution"  OR  "ambient air quality"  OR  "air pollutant*"  OR  "household air pollution"  OR  "household air quality"  OR  "traffic related air pollution*"  OR  "particulate matter"  OR  particulate*  OR  pm2.5  OR  pm10  OR  "carbon monoxide"  OR  co  OR  "Sulfur dioxide"  OR  so2  OR  "nitrogen dioxide"  OR  no2  OR  "nitric oxide*"  OR  "nitrogen oxide*"  OR  "nitrous oxide*"  OR  ozone  OR  o3  OR  biomass  OR  biofuel*  OR  emission*  OR  combustion  OR  smoke  OR  smog  OR  "gaseous pollutant*"  OR  "particle pollution*" ) | [6,018,703](https://www-scopus-com.wwwproxy1.library.unsw.edu.au/search/history/results.uri?origin=searchhistory&shid=10) |
|  | TITLE-ABS-KEY ( "under-five child*"  OR  baby  OR  infant  OR  child*  OR  newborn*  OR  neonate*  OR  neonatal  OR  "young child*"  OR  "pediatric patient*"  OR  "paediatric patient*" ) | [4,797,181](https://www-scopus-com.wwwproxy1.library.unsw.edu.au/search/history/results.uri?origin=searchhistory&shid=11) |
|  | TITLE-ABS-KEY ( morbidity  OR  mortality  OR  death  OR  "respiratory tract infection*"  OR  "respiratory infection*"  OR  "respiratory tract disease*"  OR  "respiratory disease*"  OR  pneumonia*  OR  asthma  OR  hospitalization*  OR  hospitalisation*  OR  urti  OR  lrti ) | [4,395,247](https://www-scopus-com.wwwproxy1.library.unsw.edu.au/search/history/results.uri?origin=searchhistory&shid=12) |
|  | TITLE-ABS-KEY ( "Sub Saharan Africa"  OR  ssa  OR  angola  OR  benin  OR  botswana  OR  "Burkina Faso"  OR  burundi  OR  "Cabo Verde"  OR  cameroon  OR  "Central African Republic"  OR  chad  OR  comoros  OR  congo  OR  "Cote d’Ivoire"  OR  "Equatorial Guinea"  OR  eritrea  OR  ethiopia  OR  gabon  OR  gambia  OR  ghana  OR  guinea  OR  "Guinea- Bissau"  OR  kenya  OR  lesotho  OR  liberia  OR  madagascar  OR  malawi  OR  mali  OR  mauritania  OR  mauritius  OR  mozambique  OR  namibia  OR  niger  OR  nigeria  OR  rwanda  OR  senegal  OR  "Sierra Leone"  OR  somalia  OR  "South Africa"  OR  "South Sudan"  OR  sudan  OR  tanzania  OR  togo  OR  uganda  OR  zambia  OR  zimbabwe ) | [1,105,819](https://www-scopus-com.wwwproxy1.library.unsw.edu.au/search/history/results.uri?origin=searchhistory&shid=13) |
|  | ( TITLE-ABS-KEY ( "air pollution*" OR "air quality" OR "indoor air pollution" OR "indoor air quality" OR "outdoor air pollution" OR "outdoor air quality" OR "ambient air pollution" OR "ambient air quality" OR "air pollutant*" OR "household air pollution" OR "household air quality" OR "traffic related air pollution*" OR "particulate matter" OR particulate* OR pm2.5 OR pm10 OR "carbon monoxide" OR co OR "sulfur dioxide" OR so2 OR "nitrogen dioxide" OR no2 OR "nitric oxide*" OR "nitrogen oxide*" OR "nitrous oxide*" OR ozone OR o3 OR biomass OR biofuel* OR emission* OR combustion OR smoke OR smog OR "gaseous pollutant*" OR "particle pollution*" ) ) AND ( TITLE-ABS-KEY ( "under-five child*" OR baby OR infant OR child* OR newborn* OR neonate* OR neonatal OR "young child*" OR "pediatric patient*" OR "paediatric patient*" ) ) AND ( TITLE-ABS-KEY ( morbidity OR mortality OR death OR "respiratory tract infection*" OR "respiratory infection*" OR "respiratory tract disease*" OR "respiratory disease*" OR pneumonia* OR asthma OR hospitalization* OR hospitalisation* OR urti OR lrti ) ) AND ( TITLE-ABS-KEY ( "sub saharan africa" OR ssa OR angola OR benin OR botswana OR "burkina faso" OR burundi OR "cabo verde" OR cameroon OR "central african republic" OR chad OR comoros OR congo OR "cote d’ivoire" OR "equatorial guinea" OR eritrea OR ethiopia OR gabon OR gambia OR ghana OR guinea OR "guinea- bissau" OR kenya OR lesotho OR liberia OR madagascar OR malawi OR mali OR mauritania OR mauritius OR mozambique OR namibia OR niger OR nigeria OR rwanda OR senegal OR "sierra leone" OR somalia OR "south africa" OR "south sudan" OR sudan OR tanzania OR togo OR uganda OR zambia OR zimbabwe ) ) | 2,002 |
|  | ( TITLE-ABS-KEY ( "air pollution*" OR "air quality" OR "indoor air pollution" OR "indoor air quality" OR "outdoor air pollution" OR "outdoor air quality" OR "ambient air pollution" OR "ambient air quality" OR "air pollutant*" OR "household air pollution" OR "household air quality" OR "traffic related air pollution*" OR "particulate matter" OR particulate* OR pm2.5 OR pm10 OR "carbon monoxide" OR co OR "sulfur dioxide" OR so2 OR "nitrogen dioxide" OR no2 OR "nitric oxide*" OR "nitrogen oxide*" OR "nitrous oxide*" OR ozone OR o3 OR biomass OR biofuel* OR emission* OR combustion OR smoke OR smog OR "gaseous pollutant*" OR "particle pollution*" ) ) AND ( TITLE-ABS-KEY ( "under-five child*" OR baby OR infant OR child* OR newborn* OR neonate* OR neonatal OR "young child*" OR "pediatric patient*" OR "paediatric patient*" ) ) AND ( TITLE-ABS-KEY ( morbidity OR mortality OR death OR "respiratory tract infection*" OR "respiratory infection*" OR "respiratory tract disease*" OR "respiratory disease*" OR pneumonia* OR asthma OR hospitalization* OR hospitalisation* OR urti OR lrti ) ) AND ( TITLE-ABS-KEY ( "sub saharan africa" OR ssa OR angola OR benin OR botswana OR "burkina faso" OR burundi OR "cabo verde" OR cameroon OR "central african republic" OR chad OR comoros OR congo OR "cote d’ivoire" OR "equatorial guinea" OR eritrea OR ethiopia OR gabon OR gambia OR ghana OR guinea OR "guinea- bissau" OR kenya OR lesotho OR liberia OR madagascar OR malawi OR mali OR mauritania OR mauritius OR mozambique OR namibia OR niger OR nigeria OR rwanda OR senegal OR "sierra leone" OR somalia OR "south africa" OR "south sudan" OR sudan OR tanzania OR togo OR uganda OR zambia OR zimbabwe ) ) AND ( LIMIT-TO ( EXACTKEYWORD , "human" ) OR LIMIT-TO ( EXACTKEYWORD , "humans" ) OR LIMIT-TO ( EXACTKEYWORD , "child" ) OR LIMIT-TO ( EXACTKEYWORD , "infant" ) OR LIMIT-TO ( EXACTKEYWORD , "preschool child" ) OR LIMIT-TO ( EXACTKEYWORD , "child, preschool" ) OR LIMIT-TO ( EXACTKEYWORD , "newborn" ) OR LIMIT-TO ( EXACTKEYWORD , "infant, newborn" ) ) AND ( LIMIT-TO ( LANGUAGE , "english" ) ) | 1,745/one study didn’t download |

**Supplementary table 4 S2: Web of science**

|  | Search statement | Result |
| --- | --- | --- |
| 1 | (((TS=(“air pollution*” OR “air quality” OR “indoor air pollution” OR “indoor air quality” OR “outdoor air pollution” OR “outdoor air quality” OR “ambient air pollution” OR “ambient air quality” OR “air pollutant*” OR “household air pollution” OR “household air quality” OR “traffic related air pollution*” OR “particulate matter” OR particulate* OR PM2.5 OR PM10 OR “carbon monoxide” OR CO OR “Sulfur dioxide” OR SO2 OR “nitrogen dioxide” OR NO2 OR “nitric oxide*” OR “nitrogen oxide*” OR “nitrous oxide*” OR ozone OR O3 OR biomass OR biofuel* OR emission* OR combustion OR smoke OR smog OR “gaseous pollutant*” OR “particle pollution*”)) AND TS=( “under-five child*” OR baby OR infant OR child* OR newborn* OR neonate* OR neonatal OR “young child*”OR pediatric* OR paediatric*)) AND TS=(morbidity OR mortality OR death OR “respiratory tract infection*” OR “respiratory infection*” OR “respiratory tract disease*” OR “respiratory disease*” OR pneumonia* OR asthma OR hospitalization* OR hospitalisation* OR URTI OR LRTI)) AND TS=(“Sub Saharan Africa” OR SSA OR Angola OR Benin OR Botswana OR “Burkina Faso” OR Burundi OR “Cabo Verde” OR Cameroon OR “Central African Republic” OR Chad OR Comoros OR Congo OR “Cote d’Ivoire” OR “Equatorial Guinea” OR Eritrea OR Ethiopia OR Gabon OR Gambia OR Ghana OR Guinea OR “Guinea- Bissau” OR Kenya OR Lesotho OR Liberia OR Madagascar OR Malawi OR Mali OR Mauritania OR Mauritius OR Mozambique OR Namibia OR Niger OR Nigeria OR Rwanda OR Senegal OR “Sierra Leone” OR Somalia OR “South Africa” OR “South Sudan” OR Sudan OR Tanzania OR Togo OR Uganda OR Zambia OR Zimbabwe) | 1,733 |
| 2 | (((TS=(“air pollution*” OR “air quality” OR “indoor air pollution” OR “indoor air quality” OR “outdoor air pollution” OR “outdoor air quality” OR “ambient air pollution” OR “ambient air quality” OR “air pollutant*” OR “household air pollution” OR “household air quality” OR “traffic related air pollution*” OR “particulate matter” OR particulate* OR PM2.5 OR PM10 OR “carbon monoxide” OR CO OR “Sulfur dioxide” OR SO2 OR “nitrogen dioxide” OR NO2 OR “nitric oxide*” OR “nitrogen oxide*” OR “nitrous oxide*” OR ozone OR O3 OR biomass OR biofuel* OR emission* OR combustion OR smoke OR smog OR “gaseous pollutant*” OR “particle pollution*”)) AND TS=( “under-five child*” OR baby OR infant OR child* OR newborn* OR neonate* OR neonatal OR “young child*”OR pediatric* OR paediatric*)) AND TS=(morbidity OR mortality OR death OR “respiratory tract infection*” OR “respiratory infection*” OR “respiratory tract disease*” OR “respiratory disease*” OR pneumonia* OR asthma OR hospitalization* OR hospitalisation* OR URTI OR LRTI)) AND TS=(“Sub Saharan Africa” OR SSA OR Angola OR Benin OR Botswana OR “Burkina Faso” OR Burundi OR “Cabo Verde” OR Cameroon OR “Central African Republic” OR Chad OR Comoros OR Congo OR “Cote d’Ivoire” OR “Equatorial Guinea” OR Eritrea OR Ethiopia OR Gabon OR Gambia OR Ghana OR Guinea OR “Guinea- Bissau” OR Kenya OR Lesotho OR Liberia OR Madagascar OR Malawi OR Mali OR Mauritania OR Mauritius OR Mozambique OR Namibia OR Niger OR Nigeria OR Rwanda OR Senegal OR “Sierra Leone” OR Somalia OR “South Africa” OR “South Sudan” OR Sudan OR Tanzania OR Togo OR Uganda OR Zambia OR Zimbabwe) | 1,717 |
