# Supplementary material for: The effect of air pollution on morbidity and mortality among children aged under five in sub-Saharan Africa: Systematic review and meta-analysis
Source: PLoS One. 2025 Apr 10;20(4):e0320048. doi: 10.1371/journal.pone.0320048 (PMC11984980; doi:10.1371/journal.pone.0320048)
Supplement: S4 File — (DOCX) [file pone.0320048.s004.docx]

## **Supplementary file S4**: Studies screened for full-text review and data extraction

Supplementary table 1 S4: Excluded Studies and Rationale for Exclusion During the Full-Text Review Process (N=284)

| **No** . | **Study reference** | **Title** | **Published Year** | **Journal** | **Volume** | **DOI** | **Exclusion reason** |
| --- | --- | --- | --- | --- | --- | --- | --- |
|  | Abayneh 2022 | Acute respiratory infections (ARIs) and factors associated with their poor clinical outcome among children under-five years attending pediatric wards of public hospital in Southwest district of Ethiopia: A prospective observational cohort study | 2022 | Eur. J. Inflamm. | 20 | 10.1177/1721727X221139266 | Ineligible outcome |
|  | Abdalla 2017 | Association of sociodemographic and household characteristics with non-fatal burns among children under the age of 10â€…years in Sudan: an exploratory secondary analysis of the Sudan Household Health Survey 2010. | 2017 | Inj Prev | 23 | 10.1136/injuryprev-2016-042208 | Ineligible outcome |
|  | Abebaw 2022 | Risk factors for childhood pneumonia at Adama Hospital Medical College, Adama, Ethiopia: a case-control study | 2022 | PNEUMONIA | 14 | 10.1186/s41479-022-00102-4 | Ineligible outcome |
|  | Achakulwisut 2019 | Global, national, and urban burdens of paediatric asthma incidence attributable to ambient NO<sub>2</sub> pollution: estimates from global datasets | 2019 | LANCET PLANETARY HEALTH | 3 | 10.1016/S2542-5196(19)30046-4 | Ineligible outcome |
|  | Achakulwisut 2019 | Global, national, and urban burdens of paediatric asthma incidence attributable to ambient NO 2 pollution: estimates from global datasets | 2019 | Lancet Planet. Health | 3 | 10.1016/S2542-5196(19)30046-4 | Study setting is out of scope |
|  | Adane 2020 | Prevalence and risk factors of acute lower respiratory infection among children living in biomass fuel using households: A community-based cross-sectional study in Northwest Ethiopia | 2020 | BMC Public Health | 20 | 10.1186/s12889-020-08515-w | Ineligible outcome |
|  | Addisu 2021 | Association of Acute Respiratory Infections with Indoor Air Pollution from Biomass Fuel Exposure among Under-Five Children in Jimma Town, Southwestern Ethiopia | 2021 | J. Environ. Public Health | 2021 | 10.1155/2021/7112548 | Ineligible outcome |
|  | Adebayo-Ojo 2022 | Short-Term Joint Effects of PM10, NO2 and SO2 on Cardio-Respiratory Disease Hospital Admissions in Cape Town, South Africa | 2022 | Int. J. Environ. Res. Public Health | 19 | 10.3390/ijerph19010495 | Ineligible outcome |
|  | Adebayo-Ojo 2022 | Short-Term Joint Effects of PM<sub>10</sub>, NO<sub>2</sub> and SO<sub>2</sub> on Cardio-Respiratory Disease Hospital Admissions in Cape Town, South Africa | 2022 | INTERNATIONAL JOURNAL OF ENVIRONMENTAL RESEARCH AND PUBLIC HEALTH | 19 | 10.3390/ijerph19010495 | Unspecified study population |
|  | Adebowale 2017 | Housing materials as predictors of under-five mortality in Nigeria: evidence from 2013 demographic and health survey | 2017 | BMC PEDIATRICS | 17 | 10.1186/s12887-016-0742-3 | Ineligible outcome |
|  | Adeleye 2022 | Mortality rate, carbon emissions, renewable energy and per capita income nexus in Sub-Saharan Africa | 2022 | PLoS ONE | 17 | 10.1371/journal.pone.0274447 | Ineligible outcome |
|  | Adesanya 2016 | A multilevel analysis of lifestyle variations in symptoms of acute respiratory infection among young children under five in Nigeria | 2016 | BMC Public Health | 16 | 10.1186/s12889-016-3565-0 | Ineligible outcome |
|  | Adesanya 2017 | Factors contributing to regional inequalities in acute respiratory infections symptoms among under-five children in Nigeria: A decomposition analysis | 2017 | Int. J. Equity Health | 16 | 10.1186/s12939-017-0626-7 | Ineligible outcome |
|  | Adesanya 2017 | Environmental Risks Associated with Symptoms of Acute Respiratory Infection among Preschool Children in North-Western and South-Southern Nigeria Communities | 2017 | INTERNATIONAL JOURNAL OF ENVIRONMENTAL RESEARCH AND PUBLIC HEALTH | 14 | 10.3390/ijerph14111396 | Ineligible outcome |
|  | Adjiwanou 2017 | Household environmental health hazards' effect on under-five mortality in sub-Saharan Africa: What can we learn from the Demographic and Health Survey? | 2017 | Global Public Health | 12 | https://dx.doi.org/10.1080/17441692.2017.1281327 | Ineligible outcome |
|  | Admasie 2018 | Children under Five from Houses of Unclean Fuel Sources and Poorly Ventilated Houses Have Higher Odds of Suffering from Acute Respiratory Infection in Wolaita-Sodo, Southern Ethiopia: A Case-Control Study | 2018 | J. Environ. Public Health | 2018 | 10.1155/2018/9320603 | Ineligible outcome |
|  | Ajumobi 2014 | High concentration of blood lead levels among young children in Bagega community, Zamfara - Nigeria and the potential risk factor. | 2014 | Pan Afr Med J | 18 Suppl 1 | 10.11694/pamj.supp.2014.18.1.4264 | Ineligible outcome |
|  | Ajumobi 2014 | High concentration of blood lead levels among young children in Bagega community, Zamfara - Nigeria and the potential risk factor | 2014 | The Pan African medical journal | 18 | https://dx.doi.org/10.11694/pamj.supp.2014.18.1.4264 | No data on air pollution |
|  | Akinyemi 2016 | Independent and combined effects of maternal smoking and solid fuel on infant and child mortality in sub-Saharan Africa | 2016 | Trop. Med. Int. Health | 21 | 10.1111/tmi.12779 | Duplication |
|  | Akinyemi 2018 | Household environment and symptoms of childhood acute respiratory tract infections in Nigeria, 2003-2013: a decade of progress and stagnation | 2018 | BMC INFECTIOUS DISEASES | 18 | 10.1186/s12879-018-3207-5 | Ineligible outcome |
|  | Akunne 2006 | Biomass solid fuel and acute respiratory infections: The ventilation factor | 2006 | Int. J. Hyg. Environ. Health | 209 | 10.1016/j.ijheh.2006.04.009 | Ineligible outcome |
|  | Albers 2015 | Household fuel use and child respiratory ill health in two towns in Mpumalanga, South Africa | 2015 | S. Afr. Med. J. | 105 | 10.7196/SAMJnew.7934 | Ineligible outcome |
|  | Alemayehu 2019 | Risk factors of acute respiratory infections among under five children attending public hospitals in southern Tigray, Ethiopia, 2016/2017 | 2019 | BMC PEDIATRICS | 19 | 10.1186/s12887-019-1767-1 | Ineligible outcome |
|  | Al-Janabi 2021 | A Cross-Sectional Analysis of the Association between Domestic Cooking Energy Source Type and Respiratory Infections among Children Aged under Five Years: Evidence from Demographic and Household Surveys in 37 Low-Middle Income Countries | 2021 | INTERNATIONAL JOURNAL OF ENVIRONMENTAL RESEARCH AND PUBLIC HEALTH | 18 | 10.3390/ijerph18168516 | Ineligible outcome |
|  | Aljurayyan 2011 | Human macrophage model of biomass smoke exposure shows impaired ingestion of streptococcus pneumoniae | 2011 | Thorax | 66 | https://dx.doi.org/10.1136/thoraxjnl-2011-201054b.131 | Ineligible outcome |
|  | Amadu 2023 | Assessing the combined effect of household cooking fuel and urbanicity on acute respiratory symptoms among under-five years in sub-Saharan Africa | 2023 | HELIYON | 9 | 10.1016/j.heliyon.2023.e16546 | Ineligible outcome |
|  | Ana 2009 | Environmental risk factors and health outcomes in selected communities of the Niger delta area, Nigeria | 2009 | Perspect. Public Health | 129 | 10.1177/1466424008094803 | Ineligible outcome |
|  | Ana 2015 | Indoor airborne microbial burden and risk of acute respiratory infections among children under five in Ibadan, Nigeria | 2015 | Indoor and Built Environment | 24 | https://dx.doi.org/10.1177/1420326X13499171 | Ineligible outcome |
|  | Anderson 1978 | Respiratory abnormalities in papua new Guinea children: The effects of locality and domestic wood smoke pollution | 1978 | Int. J. Epidemiol. | 7 | 10.1093/ije/7.1.63 | Ineligible outcome |
|  | Andualem 2020 | Acute respiratory symptoms and its associated factors among mothers who have under five-years-old children in northwest, Ethiopia | 2020 | Environ. Health Prev. Med. | 25 | 10.1186/s12199-020-00859-4 | Ineligible outcome |
|  | Andualem 2020 | Respiratory symptoms and associated risk factors among under-five children in Northwest, Ethiopia: Community based cross-sectional study | 2020 | Multidiscip. Resp. Med. | 15 | 10.4081/mrm.2020.685 | Ineligible outcome |
|  | Andualem 2020 | Pneumonia among Under-Five Children in Northwest Ethiopia: Prevalence and Predictors-A Community-Based Cross-Sectional Study | 2020 | INTERNATIONAL JOURNAL OF PEDIATRICS | 2020 | 10.1155/2020/3464907 | Ineligible outcome |
|  | Anenberg 2022 | Long-term trends in urban NO2 concentrations and associated paediatric asthma incidence: estimates from global datasets | 2022 | Lancet Planet. Health | 6 | 10.1016/S2542-5196(21)00255-2 | Ineligible outcome |
|  | Anenberg 2022 | Long-term trends in urban NO<sub>2</sub> concentrations and associated paediatric asthma incidence: estimates from global datasets | 2022 | LANCET PLANETARY HEALTH | 6 |  | Ineligible outcome |
|  | Anonymous 1991 | Indoor air pollution in developing countries | 1991 | Lancet | 337 |  | Review studies |
|  | Ardrey 2016 | The cooking and pneumonia study (CAPS) in Malawi: A nested pilot of photovoice participatory research methodology | 2016 | PLoS ONE | 11 | 10.1371/journal.pone.0156500 | Qualitative study |
|  | Armah 2015 | Indoor Air Pollution and Health in Ghana: Self-Reported Exposure to Unprocessed Solid Fuel Smoke | 2015 | ECOHEALTH | 12 | 10.1007/s10393-013-0883-x | Unspecified study population |
|  | Armstrong 1991 | Indoor airpollution exposure and lower respiratory infections in young gambian children | 1991 | Int. J. Epidemiol. | 20 | 10.1093/ije/20.2.424 | Ineligible outcome |
|  | Asante 2016 | Childhood respiratory morbidity and cooking practices among households in a predominantly rural area of Ghana | 2016 | Afr. J. Infect. Dis. | 10 | 10.21010/ajid.v10i2.5 | Ineligible outcome |
|  | Azuh 2021 | Socio-demographic and environmental determinants of child mortality in rural communities of Ogun State, Nigeria | 2021 | AFRICAN JOURNAL OF REPRODUCTIVE HEALTH | 25 | 10.29063/ajrh2021/v25i5s.15 | No full text available |
|  | Bailis 2005 | Mortality and greenhouse gas impacts of biomass and petroleum energy futures in Africa. | 2005 | Science | 308 | 10.1126/science.1106881 | Study setting is out of scope |
|  | Barnes 2009 | Household energy, indoor air pollution and child respiratory health in South Africa | 2009 | JOURNAL OF ENERGY IN SOUTHERN AFRICA | 20 | 10.17159/2413-3051/2009/v20i1a3296 | Qualitative study |
|  | Bateman 2009 | Systems for the management of respiratory disease in primary care - An international series: South Africa | 2009 | Prim. Care Respir. J. | 18 | 10.3132/pcrj.2009.00009 | No data on air pollution |
|  | Bekele 2017 | Factors associated with outcomes of severe pneumonia in children aged 2 months to 59 months at jimma university specialized hospital, southwest Ethiopia | 2017 | Curr. Paediatr. Res. | 21 |  | Ineligible outcome |
|  | Bergstra 1988 | Domestic air pollution and respiratory infections in young Gambian children: Analysis of data from a cross-sectional study | 1988 | ENVIRON. TROP. HEALTH REP. | | | Ineligible outcome |
|  | Bortey-Sam 2018 | Association between human exposure to heavy metals/metalloid and occurrences of respiratory diseases, lipid peroxidation and DNA damage in Kumasi, Ghana | 2018 | Environ. Pollut. | 235 | 10.1016/j.envpol.2017.12.005 | Ineligible outcome |
|  | Bruce 2000 | Indoor air pollution in developing countries: a major environmental and public health challenge | 2000 | BULLETIN OF THE WORLD HEALTH ORGANIZATION | 78 |  | Review studies |
|  | Buchner 2015 | Cooking and season as risk factors for acute lower respiratory infections in African children: A cross-sectional multi-country analysis | 2015 | PLoS ONE | 10 | 10.1371/journal.pone.0128933 | Ineligible outcome |
|  | Cai 2021 | Ambient air pollution and respiratory health in sub-saharan african children: A cross-sectional analysis | 2021 | Int. J. Environ. Res. Public Health | 18 | 10.3390/ijerph18189729 | Ineligible outcome |
|  | Cai 2021 | Late Breaking Abstract- Ambient air pollution and respiratory health in sub-Saharan African children: a cross-sectional analysis | 2021 | European Respiratory Journal | 58 | https://dx.doi.org/10.1183/13993003.congress-2021.PA1798 | Ineligible outcome |
|  | Campbell 1997 | Indoor air pollution and acute lower respiratory infections in young Gambian children. | 1997 | Health Bull (Edinb) | 55 |  | Ineligible outcome |
|  | Carlos 2023 | Air pollution exposure when cooking with electricity compared to gas | 2023 | medRxiv |  | 10.1101/2023.04.10.23288249 | Ineligible outcome |
|  | CarriÃ³n 2019 | Examining the relationship between household air pollution and infant microbial nasal carriage in a Ghanaian cohort | 2019 | Environ. Int. | 133 | 10.1016/j.envint.2019.105150 | Ineligible outcome |
|  | Chakaya 2021 | Global Tuberculosis Report 2020 - Reflections on the Global TB burden, treatment and prevention efforts. | 2021 | Int J Infect Dis | 113 Suppl 1 | 10.1016/j.ijid.2021.02.107 | Ineligible outcome |
|  | Chanie 2021 | Predictors of community acquired childhood pneumonia among 2-59 months old children in the Amhara Region, Ethiopia | 2021 | BMC Pulmonary Medicine | 21 | https://dx.doi.org/10.1186/s12890-021-01548-w | Ineligible outcome |
|  | Chinawa 2020 | Clinical profile, severity pattern and socio-demographic risk factors of acute lower respiratory tract infection (Alrti) in children in Enugu, Nigeria | 2020 | Curr. Respir. Med. Rev. | 16 | 10.2174/1573398X16999200727172120 | Ineligible outcome |
|  | Chineke 2009 | Harmattan Particulate Concentration and Health Impacts in Sub-Saharan Africa | 2009 | AFRICAN REVIEW OF PHYSICS | 3 |  | Ineligible outcome |
|  | ChirilÄƒ 1990 | Houses and allergic respiratory syndromes. | 1990 | Med Interne | 28 |  | Study setting is out of scope |
|  | Coker 2018 | A narrative review on the human health effects of ambient air pollution in sub-saharan africa: An urgent need for health effects studies | 2018 | Int. J. Environ. Res. Public Health | 15 | 10.3390/ijerph15030427 | Review study |
|  | Coker 2020 | Household air pollution profiles associated with persistent childhood cough in urban Uganda | 2020 | Environ. Int. | 136 | 10.1016/j.envint.2020.105471 | Ineligible outcome |
|  | Collings 1990 | Indoor woodsmoke pollution causing lower respiratory disease in children | 1990 | Trop. Doct. | 20 | 10.1177/004947559002000403 | Ineligible outcome |
|  | Daffe 2022 | Household level of air pollution and its impact on the occurrence of Acute Respiratory Illness among children under five: secondary analysis of Demographic and Health Survey in West Africa | 2022 | BMC Public Health | 22 | 10.1186/s12889-022-14611-w | Ineligible outcome |
|  | Dagne 2020 | Acute respiratory infection and its associated factors among children under-five years attending pediatrics ward at University of Gondar Comprehensive Specialized Hospital, Northwest Ethiopia: Institution-based cross-sectional study | 2020 | BMC Pediatr. | 20 | 10.1186/s12887-020-1997-2 | Ineligible outcome |
|  | Daouda 2022 | Prediction of personal exposure to PM2.5 in mother-child pairs in rural Ghana | 2022 | JOURNAL OF EXPOSURE SCIENCE AND ENVIRONMENTAL EPIDEMIOLOGY | 32 | 10.1038/s41370-022-00420-1 | Ineligible outcome |
|  | Das 2018 | Household air pollution (HAP), microenvironment and child health: Strategies for mitigating HAP exposure in urban Rwanda | 2018 | ENVIRONMENTAL RESEARCH LETTERS | 13 | 10.1088/1748-9326/aab047 | Ineligible outcome |
|  | DeLongueville 2014 | The air quality in African rural environments. Preliminary implications for health: The case of respiratory disease in the northern Benin | 2014 | Water Air Soil Pollut. | 225 | 10.1007/s11270-014-2186-4 | Ineligible outcome |
|  | Demissie 2021 | Acute lower respiratory tract infections and associated factors among under-five children visiting Wolaita Sodo University Teaching and Referral Hospital, Wolaita Sodo, Ethiopia | 2021 | BMC Pediatrics | 21 | https://dx.doi.org/10.1186/s12887-021-02888-6 | Ineligible outcome |
|  | denBoon 2007 | Association between passive smoking and infection with <i>Mycobacterium tuberculosis</i> in children | 2007 | PEDIATRICS | 119 | 10.1542/peds.2006-1796 | Unspecified study population |
|  | Desalu 2009 | Self-Reported Risk Factors of Asthma in a Nigerian Adult Population | 2009 | TURKISH THORACIC JOURNAL | 10 |  | Unspecified study population |
|  | Dherani 2022 | Association between household air pollution and nasopharyngeal pneumococcal carriage in Malawian infants (MSCAPE): a nested, prospective, observational study | 2022 | Lancet Global Health | 10 | 10.1016/S2214-109X(21)00405-8 | Ineligible outcome |
|  | Diboulo 2012 | Weather and mortality: A 10 year retrospective analysis of the nouna health and demographic surveillance system, Burkina Faso | 2012 | Global Health Action | 5 | 10.3402/gha.v5i0.19078 | Ineligible outcome |
|  | Dida 2022 | Factors predisposing women and children to indoor air pollution in rural villages, Western Kenya | 2022 | ARCHIVES OF PUBLIC HEALTH | 80 | 10.1186/s13690-022-00791-9 | Ineligible outcome |
|  | Dietler 2021 | Housing conditions and respiratory health in children in mining communities: An analysis of data from 27 countries in sub-Saharan Africa | 2021 | ENVIRONMENTAL IMPACT ASSESSMENT REVIEW | 89 | 10.1016/j.eiar.2021.106591 | Ineligible outcome |
|  | Dionisio 2008 | Measuring the exposure of infants and children to indoor air pollution from biomass fuels in The Gambia | 2008 | INDOOR AIR | 18 | 10.1111/j.1600-0668.2008.00533.x | Ineligible outcome |
|  | Dionisio 2011 | Measuring the exposure of infants and children to indoor air pollution from biomass fuels in the Gambia | 2011 | Epidemiology | 22 | https://dx.doi.org/10.1097/01.ede.0000392029.80000.89 | Ineligible outcome |
|  | Dionisio 2012 | Household Concentrations and Exposure of Children to Particulate Matter from Biomass Fuels in The Gambia | 2012 | ENVIRONMENTAL SCIENCE & TECHNOLOGY | 46 | 10.1021/es203047e | Ineligible outcome |
|  | Dionisio 2012 | The exposure of infants and children to carbon monoxide from biomass fuels in the Gambia: A measurement and modeling study | 2012 | J. Expos. Sci. Environ. Epidemiol. | 22 | 10.1038/jes.2011.47 | No data on air pollution |
|  | Dooyema 2012 | Outbreak of fatal childhood lead poisoning related to artisanal gold mining in northwestern Nigeria, 2010 | 2012 | Environmental Health Perspectives | 120 | https://dx.doi.org/10.1289/ehp.1103965 | No data on air pollution |
|  | Dutta 2021 | Impact of prenatal and postnatal household air pollution exposure on lung function of 2-year old Nigerian children by oscillometry | 2021 | Sci. Total Environ. | 755 | 10.1016/j.scitotenv.2020.143419 | Ineligible outcome |
|  | Eghomwanre 2022 | Concentrations of indoor gaseous air pollutants and risk factors associated with childhood asthma in Benin City, Nigeria | 2022 | Environ. Monit. Assess. | 194 | 10.1007/s10661-022-10026-7 | Ineligible outcome |
|  | Eghomwanre 2022 | Levels of indoor particulate matter and association with asthma in children in Benin City, Nigeria | 2022 | Environ. Monit. Assess. | 194 | 10.1007/s10661-022-10135-3 | Ineligible outcome |
|  | Ehrlich 1996 | Risk factors for childhood asthma and wheezing. Importance of maternal and household smoking. | 1996 | Am J Respir Crit Care Med | 154 | 10.1164/ajrccm.154.3.8810605 | Unspecified study population |
|  | Elizabeth 2013 | Air pollution impacts on pregnant women and low birth weight in Kenya | 2013 | Journal of the Neurological Sciences | 333 | https://dx.doi.org/10.1016/j.jns.2013.07.2148 | Ineligible outcome |
|  | Elshazali 2021 | Paediatric dilated cardiomyopathy in Khartoum state, Sudan: A prospective study | 2021 | BMJ Paediatrics Open | 5 | https://dx.doi.org/10.1136/bmjpo-2020-000970 | No data on air pollution |
|  | Etchie 2018 | The gains in life expectancy by ambient PM2.5 pollution reductions in localities in Nigeria | 2018 | Environ. Pollut. | 236 | 10.1016/j.envpol.2018.01.034 | Ineligible outcome |
|  | Ezeh 2014 | The effect of solid fuel use on childhood mortality in Nigeria: Evidence from the 2013 cross-sectional household survey | 2014 | Environ. Health Global Access Sci. Sour. | 13 | 10.1186/1476-069X-13-113 | Duplication |
|  | Ezzati 2000 | The contributions of emissions and spatial microenvironments to exposure to indoor air pollution from biomass combustion in Kenya | 2000 | Environ. Health Perspect. | 108 | 10.1289/ehp.00108833 | Ineligible outcome |
|  | Ezzati 2001 | Indoor air pollution from biomass combustion and acute respiratory infections in Kenya: An exposure-response study | 2001 | Lancet | 358 | 10.1016/S0140-6736(01)05777-4 | Ineligible outcome |
|  | Ezzati 2001 | Quantifying the effects of exposure to indoor air pollution from biomass combustion on acute respiratory infections in developing countries | 2001 | Environ. Health Perspect. | 109 | 10.1289/ehp.01109481 | Ineligible outcome |
|  | Fakunle 2016 | Housing quality and risk of acute respiratory infections among hospitalized children under five in Ibadan, Nigeria | 2016 | Indoor and Built Environment | 25 | https://dx.doi.org/10.1177/1420326X15599044 | No data on air pollution |
|  | Fakunle 2022 | Indoor bacterial and fungal aerosols as predictors of lower respiratory tract infections among under-five children in Ibadan, Nigeria | 2022 | BMC Pulmonary Medicine | 22 | https://dx.doi.org/10.1186/s12890-022-02271-w | No data on air pollution |
|  | Fakunle 2023 | Exposure-response relationship of residential dampness and mold damage with severe lower respiratory tract infections among under-five children in Nigeria | 2023 | Environmental Epidemiology | 7 | https://dx.doi.org/10.1097/EE9.0000000000000247 | No data on air pollution |
|  | Fisher 2021 | Air pollution and development in Africa: impacts on health, the economy, and human capital | 2021 | Lancet Planet. Health | 5 | 10.1016/S2542-5196(21)00201-1 | Ineligible outcome |
|  | Flanagan 2022 | Ambient and indoor air pollution exposure and adverse birth outcomes in Adama, Ethiopia | 2022 | Environ. Int. | 164 | 10.1016/j.envint.2022.107251 | Duplication |
|  | Foote 2013 | Impact of locally-produced, ceramic cookstoves on respiratory disease in children in rural western Kenya | 2013 | Am. J. Trop. Med. Hyg. | 88 | 10.4269/ajtmh.2012.12-0496 | Ineligible outcome |
|  | Frostad 2022 | Mapping development and health effects of cooking with solid fuels in low-income and middle-income countries, 2000â€“18: a geospatial modelling study | 2022 | Lancet Global Health | 10 | 10.1016/S2214-109X(22)00332-1 | Ineligible outcome |
|  | Fullerton 2009 | Biomass fuel use and indoor air pollution in homes in Malawi | 2009 | Occup. Environ. Med. | 66 | 10.1136/oem.2008.045013 | Ineligible outcome |
|  | Gedikondele 2011 | Nose and throat complications associated with passive smoking among Congolese school children. | 2011 | Afr Health Sci | 11 |  | Unspecified study population |
|  | Geremew 2020 | Place of food cooking is associated with acute respiratory infection among under-five children in Ethiopia: multilevel analysis of 2005â€“2016 Ethiopian Demographic Health Survey data | 2020 | Trop. Med. Health | 48 | 10.1186/s41182-020-00283-y | Ineligible outcome |
|  | Getaneh 2019 | Determinants of pneumonia among 2-59 months old children at Debre Markos referral hospital, Northwest Ethiopia: A case-control study | 2019 | BMC Pulm. Med. | 19 | 10.1186/s12890-019-0908-5 | Ineligible outcome |
|  | GivenMoonga 2021 | Geospatial approach to investigate spatial clustering and hotspots of blood lead levels in children within Kabwe, Zambia | 2021 | medRxiv |  | 10.1101/2021.03.16.21253682 | Ineligible outcome |
|  | Gizaw 2019 | Sanitation predictors of childhood morbidities in Ethiopia: Evidence from Dabat Health and Demographic Surveillance System | 2019 | Environmental Health and Preventive Medicine | 24 | <https://dx.doi.org/10.1186/s12199-019-0801-0> | Ineligible outcome |
|  | Gninafon 2011 | Exposure to combustion of solid fuel and tuberculosis: a matched case-control study | 2011 | EUROPEAN RESPIRATORY JOURNAL | 38 | 10.1183/09031936.00104610 | Unspecified study population |
|  | Gochicoa-Rangel 2011 | Pollution/biomass fuel exposure and respiratory illness in children | 2011 | Paediatric Respiratory Reviews | 12 |  | No data on air pollution |
|  | Gomez 2016 | Higher exposure to household air pollution is associated with acute lower respiratory illness in human immunodeficiency virus-infected Kenyan infants | 2016 | Open Forum Infectious Diseases | 3 | https://dx.doi.org/10.1093/ofid/ofw172.17 | Ineligible outcome |
|  | Grasemann 1997 | Metabolites of nitric oxide in the lower respiratory tract of children | 1997 | EUROPEAN JOURNAL OF PEDIATRICS | 156 | 10.1007/s004310050667 | Ineligible outcome |
|  | Gray 2010 | Childhood pneumonia in low and middle income countries: Burden, prevention and management | 2010 | Open Infect. Dis. J. | 4 | 10.2174/1874279301004020074 | Review study |
|  | Gray 2014 | Early determinants of lung function in African infants | 2014 | Pediatric Pulmonology | 49 | https://dx.doi.org/10.1002/ppul.23068 | Ineligible outcome |
|  | HÃ¼ls 2020 | Genetic susceptibility to asthma increases the vulnerability to indoor air pollution | 2020 | Eur. Respir. J. | 55 | 10.1183/13993003.01831-2019 | Ineligible outcome |
|  | Haefliger 2009 | Mass lead intoxication from informal used lead-acid battery recycling in Dakar, Senegal | 2009 | Environ. Health Perspect. | 117 | 10.1289/ehp.0900696 | Ineligible outcome |
|  | Hailemanam 2018 | Effect of Nutritional Status and Associated Factors on Pneumonia Treatment Outcome among Under-Five Children at St. Paul's Hospital Millennium Medical College, Addis Ababa, Ethiopia | 2018 | INTERNATIONAL JOURNAL OF CHILD HEALTH AND NUTRITION | 7 | 10.6000/1929-4247.2018.07.04.9 | No data on air pollution |
|  | HAPINInvestigators 2020 | Air Pollutant Exposure and Stove Use Assessment Methods for the Household Air Pollution Intervention Network (HAPIN) Trial | 2020 | ENVIRONMENTAL HEALTH PERSPECTIVES | 128 | 10.1289/EHP6422 | Ineligible outcome |
|  | Hassen 2020 | Determinants of acute respiratory infection (ARI) among under-five children in rural areas of Legambo District, South Wollo Zone, Ethiopia: A matched caseâ€“control study | 2020 | Int. J. Infect. Dis. | 96 | 10.1016/j.ijid.2020.05.012 | Ineligible outcome |
|  | Havens 2018 | The cooking and pneumonia study (CAPS) in Malawi: A cross-sectional assessment of carbon monoxide exposure and carboxyhemoglobin levels in children under 5 years old | 2018 | Int. J. Environ. Res. Public Health | 15 | 10.3390/ijerph15091936 | Ineligible outcome |
|  | Heft-Neal 2018 | Robust relationship between air quality and infant mortality in Africa | 2018 | Nature | 559 | 10.1038/s41586-018-0263-3 | Study setting is out of scope |
|  | Heft-Neal 2020 | Dust pollution from the Sahara and African infant mortality | 2020 | NATURE SUSTAINABILITY | 3 | 10.1038/s41893-020-0562-1 | Ineligible outcome |
|  | Hooper 2015 | Estimating asthma prevalence in rural Senegal | 2015 | American Journal of Respiratory and Critical Care Medicine | 191 |  | Unspecified study population |
|  | Houle 2023 | Household structure, composition and child mortality in the unfolding antiretroviral therapy era in rural South Africa: comparative evidence from population surveillance, 2000-2015 | 2023 | BMJ Open | 13 | 10.1136/bmjopen-2022-070388 | No data on air pollution |
|  | Howlett-Downing 2023 | Health risk assessment of PM2.5 and PM2.5-bound trace elements in Pretoria, South Africa | 2023 | J. Environ. Sci. Health Part A Toxic Hazard. Subst. Environ. Eng. | 58 | 10.1080/10934529.2023.2186653 | Ineligible outcome |
|  | Howlett-Downing 2023 | Health risk assessment of PM<sub>2.5</sub> and PM<sub>2.5</sub>-bound trace elements in Pretoria, South Africa | 2023 | JOURNAL OF ENVIRONMENTAL SCIENCE AND HEALTH PART A-TOXIC/HAZARDOUS SUBSTANCES & ENVIRONMENTAL ENGINEERING | 58 | 10.1080/10934529.2023.2186653 | Ineligible outcome |
|  | Hu 2023 | Effect of PM2.5 air pollution on the global burden of lower respiratory infections, 1990â€“2019: A systematic analysis from the Global Burden of Disease Study 2019 | 2023 | J. Hazard. Mater. | 459 | 10.1016/j.jhazmat.2023.132215 | Ineligible outcome |
|  | Hussein 2021 | Fuel type use and risk of respiratory symptoms: A cohort study of infants in the Northern region of Ghana | 2021 | SCIENCE OF THE TOTAL ENVIRONMENT | 755 | 10.1016/j.scitotenv.2020.142501 | Ineligible outcome |
|  | IMPALAConsortium 2022 | Household Microenvironment and Under-Fives Health Outcomes in Uganda: Focusing on Multidimensional Energy Poverty and Women Empowerment Indices | 2022 | INTERNATIONAL JOURNAL OF ENVIRONMENTAL RESEARCH AND PUBLIC HEALTH | 19 | 10.3390/ijerph19116684 | Ineligible outcome |
|  | Jafta 2017 | Indoor air quality of low and middle income urban households in Durban, South Africa | 2017 | Environ. Res. | 156 | 10.1016/j.envres.2017.03.008 | Ineligible outcome |
|  | Jafta 2019 | Association of childhood pulmonary tuberculosis with exposure to indoor air pollution: a case control study | 2019 | BMC public health | 19 | <https://dx.doi.org/10.1186/s12889-019-6604-9> | Unspecified study population |
|  | Jestin-Guyon 2023 | Impact of biomass fuel smoke on respiratory health of children under 15 years old in Madagascar | 2023 | RESPIRATORY MEDICINE AND RESEARCH | 83 | 10.1016/j.resmer.2023.100989 | Unspecified study population |
|  | Kang 2023 | Trends of global and regional aetiologies, risk factors and mortality of lower respiratory infections from 1990 to 2019: An analysis for the Global Burden of Disease Study 2019 | 2023 | Respirology | 28 | 10.1111/resp.14389 | Unspecified study population |
|  | Karimi 2020 | Saharan sand and dust storms and neonatal mortality: Evidence from Burkina Faso | 2020 | Sci. Total Environ. | 729 | 10.1016/j.scitotenv.2020.139053 | Ineligible outcome |
|  | Kawano 2022 | Association between satellite-detected tropospheric nitrogen dioxide and acute respiratory infections in children under age five in Senegal: spatio-temporal analysis | 2022 | BMC Public Health | 22 | 10.1186/s12889-022-12577-3 | Ineligible outcome |
|  | Keleb 2020 | Pneumonia remains a leading public health problem among under-five children in peri-urban areas of north-eastern Ethiopia | 2020 | PLoS ONE | 15 | 10.1371/journal.pone.0235818 | Ineligible outcome |
|  | Kelly 2015 | The effect of exposure to wood smoke on outcomes of childhood pneumonia in Botswana | 2015 | Int. J. Tuberc. Lung Dis. | 19 | 10.5588/ijtld.14.0557 | Ineligible outcome |
|  | Kelly 2018 | From kitchen to classroom: Assessing the impact of cleaner burning biomass-fuelled cookstoves on primary school attendance in Karonga district, northern Malawi | 2018 | PLoS ONE | 13 | 10.1371/journal.pone.0193376 | Unspecified study population |
|  | Kilabuko 2007 | Effects of cooking fuels on acute respiratory infections in children in Tanzania | 2007 | Int. J. Environ. Res. Public Health | 4 | 10.3390/ijerph200704040003 | Ineligible outcome |
|  | Kilabuko 2007 | Air quality and acute respiratory illness in biomass fuel using homes in Bagamoyo, Tanzania | 2007 | Int. J. Environ. Res. Public Health | 4 | 10.3390/ijerph2007010007 | Ineligible outcome |
|  | Kinney 2021 | Prenatal and Postnatal Household Air Pollution Exposures and Pneumonia Risk: Evidence From the Ghana Randomized Air Pollution and Health Study | 2021 | Chest | 160 | 10.1016/j.chest.2021.06.080 | Ineligible outcome |
|  | Kirby 2016 | Assessing use, exposure and health impacts of an advanced water filter and advanced cookstove distribution program in Rural Rwanda | 2016 | American Journal of Tropical Medicine and Hygiene | 95 | <https://dx.doi.org/10.4269/ajtmh.abstract2016> | Ineligible outcome |
|  | Kirenga 2018 | Lung function of children at three sites of varying ambient air pollution levels in Uganda: A cross sectional comparative study | 2018 | International Journal of Environmental Research and Public Health | 15 | https://dx.doi.org/10.3390/ijerph15122653 | Unspecified study population |
|  | Kishamawe 2019 | Trends, patterns and causes of respiratory disease mortality among inpatients in Tanzania, 2006â€“2015 | 2019 | Trop. Med. Int. Health | 24 | 10.1111/tmi.13165 | Ineligible outcome |
|  | Kodros 2018 | Quantifying the Contribution to Uncertainty in Mortality Attributed to Household, Ambient, and Joint Exposure to PM2.5 From Residential Solid Fuel Use | 2018 | GEOHEALTH | 2 | 10.1002/2017GH000115 | Ineligible outcome |
|  | Kodros 2018 | Quantifying the Contribution to Uncertainty in Mortality Attributed to Household, Ambient, and Joint Exposure to PM<sub>2.5</sub> From Residential Solid Fuel Use | 2018 | GEOHEALTH | 2 | 10.1002/2017GH000115 | Unspecified study population |
|  | Kouao 2019 | Exposure to indoor and outdoor air pollution among children under five years old in urban area | 2019 | GLOBAL JOURNAL OF ENVIRONMENTAL SCIENCE AND MANAGEMENT-GJESM | 5 | 10.22034/gjesm.2019.02.05 | Ineligible outcome |
|  | Kulkarni 2005 | Carbon loading of alveolar macrophages in adults and children exposed to biomass smoke particles | 2005 | Sci. Total Environ. | 345 | 10.1016/j.scitotenv.2004.10.016 | Ineligible outcome |
|  | Kumie 2009 | Magnitude of indoor NO2 from biomass fuels in rural settings of Ethiopia | 2009 | INDOOR AIR | 19 | 10.1111/j.1600-0668.2008.00555.x | Ineligible outcome |
|  | Larson 2022 | Long-Term PM2.5 Exposure Is Associated with Symptoms of Acute Respiratory Infections among Children under Five Years of Age in Kenya, 2014 | 2022 | Int. J. Environ. Res. Public Health | 19 | 10.3390/ijerph19052525 | Ineligible outcome |
|  | Larson 2022 | Long-Term PM<sub>2.5</sub> Exposure Is Associated with Symptoms of Acute Respiratory Infections among Children under Five Years of Age in Kenya, 2014 | 2022 | INTERNATIONAL JOURNAL OF ENVIRONMENTAL RESEARCH AND PUBLIC HEALTH | 19 | 10.3390/ijerph19052525 | Ineligible outcome |
|  | Lawande 1979 | Recovery of soil Amebas from the nasal passages of children during the dusty harmattan period in Zaria. | 1979 | Am J Clin Pathol | 71 | 10.1093/ajcp/71.2.201 | Ineligible outcome |
|  | Lawoyin 2001 | Risk factors for infant mortality in a rural community in Nigeria | 2001 | Journal of The Royal Society for the Promotion of Health | 121 | http://dx.doi.org/10.1177/146642400112100213 | Ineligible outcome |
|  | Lee 2019 | Prenatal household air pollution is associated with impaired infant lung function with sex-specific effects | 2019 | Am. J. Respir. Crit. Care Med. | 199 | 10.1164/rccm.201804-0694OC | Ineligible outcome |
|  | Lee 2019 | Prenatal Household Air Pollution Is Associated with Impaired Infant Lung Function with Sex-Specific Effects Evidence from GRAPHS, a Cluster Randomized Cookstove Intervention Trial | 2019 | AMERICAN JOURNAL OF RESPIRATORY AND CRITICAL CARE MEDICINE | 199 | 10.1164/rccm.201804-0694OC | Ineligible outcome |
|  | Lelieveld 2018 | Age-dependent health risk from ambient air pollution: a modelling and data analysis of childhood mortality in middle-income and low-income countries | 2018 | Lancet Planet. Health | 2 | 10.1016/S2542-5196(18)30147-5 | Ineligible outcome |
|  | Li 2023 | Source sectors underlying PM2.5-related deaths among children under 5 years of age in 17 low- and middle-income countries | 2023 | Environ. Int. | 172 | 10.1016/j.envint.2023.107756 | Study setting is out of scope |
|  | Liu 2022 | Lack of Associations between Environmental Exposures and Environmental Enteric Dysfunction among 18-Month-Old Children in Rural Malawi | 2022 | International Journal of Environmental Research and Public Health | 19 | https://dx.doi.org/10.3390/ijerph191710891 | Ineligible outcome |
|  | Liu 2023 | Global, regional, and national burden of preterm birth attributable to ambient and household PM2.5 from 1990 to 2019: Worsening or improving? | 2023 | Sci. Total Environ. | 871 | 10.1016/j.scitotenv.2023.161975 | Study setting is out of scope |
|  | Maes 2012 | Increasing the sustainability of household cooking in developing countries: Policy implications | 2012 | RENEWABLE & SUSTAINABLE ENERGY REVIEWS | 16 | 10.1016/j.rser.2012.03.031 | Ineligible outcome |
|  | Majdan 2015 | Assessment of the biomass related indoor air pollution in kwale district in Kenya using short term monitoring | 2015 | Afr. Health Sci. | 15 | 10.4314/ahs.v15i3.35 | Ineligible outcome |
|  | Makamure 2017 | Interaction between ambient pollutant exposure, CD14 (-159) polymorphism and respiratory outcomes among children in Kwazulu-Natal, Durban | 2017 | Hum. Exp. Toxicol. | 36 | 10.1177/0960327116646620 | Ineligible outcome |
|  | Malangu 2008 | Acute poisoning at two hospitals in Kampala-Uganda | 2008 | J. Forensic Leg. Med. | 15 | 10.1016/j.jflm.2008.04.003 | Unspecified study population |
|  | Malley 2017 | Preterm birth associated with maternal fine particulate matter exposure: A global, regional and national assessment | 2017 | Environ. Int. | 101 | 10.1016/j.envint.2017.01.023 | Ineligible outcome |
|  | Markos 2019 | Determinants of under-five pneumonia at Gondar University Hospital, Northwest Ethiopia: An unmatched case-control study | 2019 | J. Environ. Public Health | 2019 | 10.1155/2019/9790216 | Ineligible outcome |
|  | Martin 1991 | Indoor air pollution in developing countries | 1991 | Lancet | 337 | 10.1016/0140-6736(91)90982-U | Study setting is out of scope |
|  | Matooane 2003 | Health risk assessment for sulfur dioxide pollution in South Durban, South Africa | 2003 | Arch. Environ. Health | 58 | 10.3200/AEOH.58.12.763-770 | Ineligible outcome |
|  | McElroy 2022 | Saharan Dust and Childhood Respiratory Symptoms in Benin | 2022 | Int. J. Environ. Res. Public Health | 19 | 10.3390/ijerph19084743 | Ineligible outcome |
|  | Meel 2008 | Unnatural deaths among children in the Transkei region of South Africa | 2008 | Medicine, Science and the Law | 48 | https://dx.doi.org/10.1258/rsmmsl.48.3.232 | Ineligible outcome |
|  | Mercedes 2022 | Long-term air pollution exposure and markers of cardiometabolic health in the National Longitudinal Study of Adolescent to Adult Health (Add Health) Study | 2022 | medRxiv |  | 10.1101/2022.12.07.22283112 | Study setting is out of scope |
|  | Mercer 2002 | Rhinitis (allergic and nonallergic) in an atopic pediatric referral population in the grasslands of inland South Africa | 2002 | Ann. Allergy Asthma Immunol. | 89 | 10.1016/S1081-1206(10)62089-3 | Ineligible outcome |
|  | Mesagan 2018 | Household Environmental Factors and Childhood Morbidity in South-Western Nigeria | 2018 | FUDAN JOURNAL OF THE HUMANITIES AND SOCIAL SCIENCES | 11 | 10.1007/s40647-017-0204-9 | Ineligible outcome |
|  | MichaelBDillon 2020 | Reducing Exposures to Airborne Particles Through Improved Filtration: A High-Level Modeling Analysis | 2020 | medRxiv |  | 10.1101/2020.05.14.20101311 | Ineligible outcome |
|  | Mishra 2003 | Indoor air pollution from biomass combustion and acute respiratory illness in preschool age children in Zimbabwe | 2003 | Int. J. Epidemiol. | 32 | 10.1093/ije/dyg240 | Ineligible outcome |
|  | Mitku 2020 | The spatial modification of the non-linear effects of ambient oxides of nitrogen during pregnancy on birthweight in a South African birth cohort | 2020 | Environ. Res. | 183 | 10.1016/j.envres.2020.109239 | Ineligible outcome |
|  | Mlambo 2023 | Air Pollution and Health in Africa: The Burden Falls on Children | 2023 | ECONOMIES | 11 | 10.3390/economies11070196 | Ineligible outcome |
|  | Morakinyo 2017 | Health risk of inhalation exposure to sub-10 mu m particulate matter and gaseous pollutants in an urban-industrial area in South Africa: an ecological study | 2017 | BMJ OPEN | 7 | 10.1136/bmjopen-2016-013941 | Ineligible outcome |
|  | Mortimer 2017 | A cleaner burning biomass-fuelled cookstove intervention to prevent pneumonia in children under 5 years old in rural Malawi (the Cooking and Pneumonia Study): a cluster randomised controlled trial | 2017 | Lancet | 389 | 10.1016/S0140-6736(16)32507-7 | Ineligible outcome |
|  | Mortimer 2017 | A Cleaner Burning Biomass-Fueled Cookstove Intervention To Prevent Pneumonia In Children Under 5 Years Old In Rural Malawi (caps): A Cluster Randomised Controlled Trial | 2017 | AMERICAN JOURNAL OF RESPIRATORY AND CRITICAL CARE MEDICINE | 195 |  | Ineligible outcome |
|  | Mortimer 2020 | Pneumonia and Exposure to Household Air Pollution in Children Under the Age of 5 Years in Rural Malawi: Findings From the Cooking and Pneumonia Study | 2020 | Chest | 158 | 10.1016/j.chest.2020.03.064 | Ineligible outcome |
|  | Mortimer 2021 | Household Air Pollution The Importance of Intervening Early | 2021 | CHEST | 160 | 10.1016/j.chest.2021.08.049 | Ineligible outcome |
|  | Moturi 2010 | Risk factors for indoor air pollution in rural households in Mauche division, Molo district, Kenya | 2010 | Afr. Health Sci. | 10 |  | Ineligible outcome |
|  | Mulambya 2020 | Trends and factors associated with acute respiratory infection among under five children in zambia: Evidence from zambiaâ€™s demographic and health surveys (1996-2014) | 2020 | Pan Afr. Med. J. | 36 | 10.11604/pamj.2020.36.197.18799 | Ineligible outcome |
|  | Muthumbi 2017 | Risk factors for community-acquired pneumonia among adults in Kenya: a case-control study | 2017 | PNEUMONIA | 9 | 10.1186/s41479-017-0041-2 | Unspecified study population |
|  | Negash 2019 | Pneumococcal Carriage, Serotype Distribution, and Risk Factors in Children with Community-Acquired Pneumonia, 5 Years after Introduction of the 10-Valent Pneumococcal Conjugate Vaccine in Ethiopia | 2019 | Open Forum Infect. Dis. | 6 | 10.1093/ofid/ofz259 | Ineligible outcome |
|  | Nkosi 2014 | Association between selected air pollution sources, wheeze, asthma, and rhinoconjunctivitis among teenagers residing in proximity to mine dump tailings in South Africa | 2014 | European Respiratory Journal | 44 |  | Ineligible outcome |
|  | Nkwocha 2008 | Effects of industrial air pollution on the respiratory health of children | 2008 | INTERNATIONAL JOURNAL OF ENVIRONMENTAL SCIENCE AND TECHNOLOGY | 5 | 10.1007/BF03326048 | Ineligible outcome |
|  | Norman 2007 | Estimating the burden of disease attributable to urban outdoor air pollution in South Africa in 2000 | 2007 | S. Afr. Med. J. | 97 |  | Ineligible outcome |
|  | Norman 2007 | Estimating the burden of disease attributable to indoor air pollution from household use of solid fuels in South Africa in 2000 | 2007 | S. Afr. Med. J. | 97 |  | Ineligible outcome |
|  | Norman 2007 | Estimating the burden of disease attributable to lead exposure in South Africa in 2000 | 2007 | South African Medical Journal | 97 |  | No data on air pollution |
|  | Norman 2010 | Estimating the burden of disease attributable to four selected environmental risk factors in South Africa | 2010 | Rev. Environ. Health | 25 | 10.1515/REVEH.2010.25.2.87 | Ineligible outcome |
|  | Nsoh 2019 | Acute respiratory infection related to air pollution in Bamenda, north west region of Cameroon | 2019 | Pan Afr. Med. J. | 32 | 10.11604/pamj.2019.32.99.15228 | Ineligible outcome |
|  | Nwaichi 2021 | Ambient particulate matter levels and health profile in residents of Choba and Mgbuoba areas of Rivers State: A cross-sectional study | 2021 | J. Air Waste Manage. Assoc. | 71 | 10.1080/10962247.2020.1846636 | Ineligible outcome |
|  | Obiazi-Odiase 2015 | The Upshot of Passive Smoke (ETS) Exposure on Pneumonia Risk in Children Under 7 Years in Nigeria | 2015 | JOURNAL OF THORACIC ONCOLOGY | 10 |  | Unspecified study population |
|  | Odiase 2012 | The effects of environmental tobacco smoke on pneumonia risk in children under 7years in northern Nigeria | 2012 | Archives of Disease in Childhood | 97 | https://dx.doi.org/10.1136/archdischild-2012-302724.0381 | Ineligible outcome |
|  | Oguntoke 2013 | Biomass energy utilisation, air quality and the health of rural women and children in Ido LGA, south-western Nigeria | 2013 | Indoor Built Environ. | 22 | 10.1177/1420326X12444784 | Ineligible outcome |
|  | Okello 2018 | Women and girls in resource poor countries experience much greater exposure to household air pollutants than men: Results from Uganda and Ethiopia | 2018 | Environ. Int. | 119 | 10.1016/j.envint.2018.07.002 | Ineligible outcome |
|  | Olaoye 2021 | Assessment of Indoor Air Quality and Health Impact associated with the use of Different Types of Cooking Stoves amongst Rural Households in Kwara State, Nigeria | 2021 | Journal of applied science & environmental management | 25 | 10.4314/jasem.v25i1.4 | Ineligible outcome |
|  | Olopade 2011 | Respiratory symptoms in children and women exposed to biomass smoke during cooking | 2011 | American Journal of Respiratory and Critical Care Medicine | 183 |  | Study setting is out of scope |
|  | Oluwole 2011 | Indoor air pollution from biomass fuel: A risk factor for pulmonary dysfunction in women and children in Ibadan, Nigeria | 2011 | American Journal of Respiratory and Critical Care Medicine | 183 |  | Unspecified study population |
|  | Oluwole 2013 | Relationship between household air pollution from biomass smoke exposure, and pulmonary dysfunction, oxidant-antioxidant imbalance and systemic inflammation in rural women and children in Nigeria. | 2013 | Glob J Health Sci | 5 | 10.5539/gjhs.v5n4p28 | Ineligible outcome |
|  | Oluwole 2017 | Household biomass fuel use, asthma symptoms severity, and asthma underdiagnosis in rural schoolchildren in Nigeria: A cross-sectional observational study | 2017 | BMC Pulm. Med. | 17 | 10.1186/s12890-016-0352-8 | Unspecified study population |
|  | Omiyefa 2012 | ENVIRONMENTAL TOBACCO SMOKE AS A RISK FACTOR TO INCREASING RESPIRATORY CHILDHOOD INFECTION AND PNEUMONIA IN SOUTH-WEST REGION NIGERIA | 2012 | JOURNAL OF THORACIC ONCOLOGY | 7 |  | Ineligible outcome |
|  | Omiyefa 2012 | Environmental Tobacco Smoke as a Risk Factor to Increasing Respiratory Childhood Infection and Pneumonia in South-West region Nigeria | 2012 | EUROPEAN JOURNAL OF CANCER | 48 | 10.1016/S0959-8049(12)71908-1 | Ineligible outcome |
|  | Omiyefa 2015 | Environmental Tobacco Smoke as a Risk Factor to Increasing Respiratory Childhood Infection and Pneumonia in South-West Region Nigeria | 2015 | JOURNAL OF THORACIC ONCOLOGY | 10 |  | Ineligible outcome |
|  | Omiyefa 2017 | Environmental Tobacco Smoke as a Risk Factor to Increasing Respiratory Childhood Infection and Pneumonia in South West Regionn Nigeria | 2017 | JOURNAL OF THORACIC ONCOLOGY | 12 |  | Ineligible outcome |
|  | Omiyefa 2018 | Second hand smoke "a threat to child's health and development" | 2018 | Tobacco Induced Diseases | 16 | https://dx.doi.org/10.18332/tid/83779 | Ineligible outcome |
|  | Onyango 2012 | Risk factors of severe pneumonia among children aged 2-59 months in western Kenya: a case control study | 2012 | PAN AFRICAN MEDICAL JOURNAL | 13 |  | Ineligible outcome |
|  | Owili 2017 | Cooking fuel and risk of under-five mortality in 23 Sub-Saharan African countries: a population-based study | 2017 | Int. J. Environ. Health Res. | 27 | 10.1080/09603123.2017.1332347 | Duplication |
|  | OwusuBoadi 2006 | Factors affecting the choice of cooking fuel, cooking place and respiratory health in the Accra metropolitan area, Ghana | 2006 | J. Biosoc. Sci. | 38 | 10.1017/S0021932005026635 | Unspecified study population |
|  | Oyedele 2022 | Carbon dioxide emission and health outcomes: is there really a nexus for the Nigerian case? | 2022 | Environ. Sci. Pollut. Res. | 29 | 10.1007/s11356-022-19365-x | Ineligible outcome |
|  | Ozoh 2017 | Factors associated with high use of kerosene for cooking in nigeria and attitude towards the use of cleaner fuel | 2017 | American Journal of Respiratory and Critical Care Medicine | 195 | https://dx.doi.org/10.1164/ajrccmconference.2017.C60 | Ineligible outcome |
|  | Pandey 1989 | Indoor air pollution in developing countries and acute respiratory infection in children | 1989 | Lancet | 1 |  | Duplication |
|  | Pandey 1989 | INDOOR AIR POLLUTION IN DEVELOPING COUNTRIES AND ACUTE RESPIRATORY INFECTION IN CHILDREN | 1989 | Lancet | 333 | 10.1016/S0140-6736(89)90015-9 | Ineligible outcome |
|  | PASSNetwork 2023 | Prenatal smoking and drinking are associated with altered newborn autonomic functions | 2023 | PEDIATRIC RESEARCH | 93 | 10.1038/s41390-022-02060-5 | Ineligible outcome |
|  | Piddock 2014 | A cross-sectional study of household biomass fuel use among a periurban population in Malawi | 2014 | Ann. Am. Thorac. Soc. | 11 | 10.1513/AnnalsATS.201311-413OC | Unspecified study population |
|  | Plumlee 2013 | Linking geological and health sciences to assess childhood lead poisoning from artisanal gold mining in Nigeria | 2013 | Environmental Health Perspectives | 121 | https://dx.doi.org/10.1289/ehp.1206051 | Ineligible outcome |
|  | PrayGod 2016 | Indoor Air Pollution and Delayed Measles Vaccination Increase the Risk of Severe Pneumonia in Children: Results from a Case-Control Study in Mwanza, Tanzania | 2016 | PLoS ONE | 11 | 10.1371/journal.pone.0160804 | Duplication |
|  | Ratjen 2000 | Airway nitric oxide in infants with acute wheezy bronchitis | 2000 | PEDIATRIC ALLERGY AND IMMUNOLOGY | 11 | 10.1034/j.1399-3038.2000.00093.x | Ineligible outcome |
|  | Rehfuess 2006 | Assessing household solid fuel use: Multiple implications for the Millennium Development Goals | 2006 | Environ. Health Perspect. | 114 | 10.1289/ehp.8603 | Ineligible outcome |
|  | Rehfuess 2009 | Solid fuel use and cooking practices as a major risk factor for ALRI mortality among African children | 2009 | J. Epidemiol. Community Health | 63 | 10.1136/jech.2008.082685 | Ineligible outcome |
|  | Roberts 2021 | Global impact of landscape fire emissions on surface level PM2.5 concentrations, air quality exposure and population mortality | 2021 | Atmos. Environ. | 252 | 10.1016/j.atmosenv.2021.118210 | Ineligible outcome |
|  | Roomaney 2022 | Estimating the burden of disease attributable to household air pollution from cooking with solid fuels in South Africa for 2000, 2006 and 2012 | 2022 | South African medical journal | 112 | 10.7196/SAMJ.2022.v112i8b.16474 | Unspecified study population |
|  | Rudan 2008 | Epidemiology and etiology of childhood pneumonia | 2008 | Bull. WHO | 86 | 10.2471/BLT.07.048769 | Ineligible outcome |
|  | Rudasingwa 2020 | Potential risk factors contributing to acute respiratory infections among under 5 years children in Rwanda | 2020 | International Journal of Infectious Diseases | 101 | https://dx.doi.org/10.1016/j.ijid.2020.09.831 | Ineligible outcome |
|  | Rumchev 2007 | Indoor air pollution from biomass combustion and respiratory symptoms of women and children in a Zimbabwean village | 2007 | Indoor Air | 17 | https://dx.doi.org/10.1111/j.1600-0668.2007.00494.x | Ineligible outcome |
|  | Rylance 2019 | Lung health and exposure to air pollution in Malawian children (CAPS): a cross-sectional study | 2019 | Thorax | 74 | 10.1136/thoraxjnl-2018-212945 | Unspecified study population |
|  | Rylance 2020 | Determinants of lung health across the life course in Sub-Saharan Africa | 2020 | Int. J. Tuberc. Lung Dis. | 24 | 10.5588/ijtld.20.0083 | Ineligible outcome |
|  | Sackou 2014 | Indoor environment and respiratory symptoms among children under five years of age in a peri-urban area of Abidjan | 2014 | Indoor Built Environ. | 23 | 10.1177/1420326X13491276 | Ineligible outcome |
|  | Sanbata 2014 | Association of biomass fuel use with acute respiratory infections among under- five children in a slum urban of Addis Ababa, Ethiopia | 2014 | BMC Public Health | 14 | 10.1186/1471-2458-14-1122 | Ineligible outcome |
|  | Schraufnagel 2020 | The health effects of ultrafine particles | 2020 | EXPERIMENTAL AND MOLECULAR MEDICINE | 52 | 10.1038/s12276-020-0403-3 | Ineligible outcome |
|  | Sebba 2022 | Children's Air Pollution Profiles in Kilimanjaro, Tanzania | 2022 | Tanzania Journal of Health Research | 23 | https://dx.doi.org/10.4314/thrb.v23i1.1S | Unspecified study population |
|  | Seedat 2019 | ENVIRONMENTAL CONTROL OF OUTDOOR ALLERGENS | 2019 | CURRENT ALLERGY & CLINICAL IMMUNOLOGY | 32 |  | Ineligible outcome |
|  | Semple 2014 | Commentary: Switching to biogas - What effect could it have on indoor air quality and human health? | 2014 | BIOMASS & BIOENERGY | 70 | 10.1016/j.biombioe.2014.01.054 | Ineligible outcome |
|  | Shayo 2022 | Household Air Pollution from Cooking Fuels Increases the Risk of Under-Fives Acute Respiratory Infection: Evidence from Population-Based Cross-Sectional Surveys in Tanzania | 2022 | Ann. of Global Health | 88 | 10.5334/aogh.3650 | Ineligible outcome |
|  | Shiferaw 2023 | Fine particulate matter air pollution and the mortality of children under five: a multilevel analysis of the Ethiopian Demographic and Health Survey of 2016 | 2023 | Front. Public Health | 11 | 10.3389/fpubh.2023.1090405 | Duplication |
|  | Shiferaw 2023 | Fine particulate matter air pollution and the mortality of children under five: a multilevel analysis of the Ethiopian Demographic and Health Survey of 2016 | 2023 | Front. Public Health | 11 | 10.3389/fpubh.2023.1090405 | Duplication |
|  | Simkovich 2020 | Design and conduct of facility-based surveillance for severe childhood pneumonia in the household air pollution intervention network (Hapin) trial | 2020 | ERJ Open Res. | 6 | 10.1183/23120541.00308-2019 | Ineligible outcome |
|  | Smith 2012 | The global burden of disease attributable to household air pollution from cooking with solid fuels: Estimates from the GBD 2010 | 2012 | Epidemiology | 23 | https://dx.doi.org/10.1097/01.ede.0000416989.22943.f3 | Study setting is out of scope |
|  | SmokeFreeFdn 2011 | THE EFFECTS OF ENVIRONMENTAL TOBACCO SMOKE (ETS) ON PNEUMONIA RISK IN CHILDREN UNDER 7 YEARS IN NORTHERN NIGERIA | 2011 | PEDIATRIC RESEARCH | 70 | 10.1038/pr.2011.763 | Unspecified study population |
|  | Sofoluwe 1968 | Smoke pollution in dwellings of infants with bronchopneumonia | 1968 | Archives of environmental health | 16 |  | Ineligible outcome |
|  | Solomon 2022 | Prevalence of pneumonia and its determinant factors among under-five children in Gamo Zone, southern Ethiopia, 2021 | 2022 | Frontiers in Pediatrics | 10 | <https://dx.doi.org/10.3389/fped.2022.1017386> | Ineligible outcome |
|  | Starnes 2023 | Childhood mortality and associated factors in Migori County, Kenya: evidence from a cross-sectional survey | 2023 | BMJ Open | 13 | 10.1136/bmjopen-2023-074056 | Duplication |
|  | Stroud 2022 | CLIMATE CHANGE, CHILDREN'S DEVELOPMENT AND THE GRIFFITHS III COMMUNITY | 2022 | Archives of Disease in Childhood | 107 | https://dx.doi.org/10.1136/archdischild-2022-rcpch.513 | Ineligible outcome |
|  | Stroupe 2018 | The use of smoke based mosquito prevention methods: A risk factor for acute respiratory infections in children? | 2018 | American Journal of Tropical Medicine and Hygiene | 99 |  | Ineligible outcome |
|  | Suhaimi 2022 | The impact of traffic-related air pollution on lung function status and respiratory symptoms among children in Klang Valley, Malaysia | 2022 | Int. J. Environ. Health Res. | 32 | 10.1080/09603123.2020.1784397 | Unspecified study population |
|  | Sulaiman 2017 | Wood fuel consumption and mortality rates in Sub-Saharan Africa: Evidence from a dynamic panel study | 2017 | Chemosphere | 177 | 10.1016/j.chemosphere.2017.03.019 | Ineligible outcome |
|  | Tamire 2021 | High Levels of Fine Particulate Matter (PM2.5) Concentrations from Burning Solid Fuels in Rural Households of Butajira, Ethiopia | 2021 | INTERNATIONAL JOURNAL OF ENVIRONMENTAL RESEARCH AND PUBLIC HEALTH | 18 | 10.3390/ijerph18136942 | Ineligible outcome |
|  | Tamire 2022 | Household fuel use and its association with potential respiratory pathogens among healthy mothers and children in Ethiopia | 2022 | PLoS ONE | 17 | 10.1371/journal.pone.0277348 | Ineligible outcome |
|  | Tang 2022 | The Association between the Burden of PM2.5-Related Neonatal Preterm Birth and Socio-Demographic Index from 1990 to 2019: A Global Burden Study | 2022 | Int. J. Environ. Res. Public Health | 19 | 10.3390/ijerph191610068 | Ineligible outcome |
|  | Taylor 2012 | Prevalence of acute respiratory infections in women and children in western sierra leone due to smoke from wood and charcoal stoves | 2012 | Int. J. Environ. Res. Public Health | 9 | 10.3390/ijerph9062252 | Ineligible outcome |
|  | Tazinya 2018 | Risk factors for acute respiratory infections in children under five years attending the Bamenda Regional Hospital in Cameroon | 2018 | BMC Pulm. Med. | 18 | 10.1186/s12890-018-0579-7 | Duplication |
|  | Teather 2015 | Air quality and children's health in rural Kenya | 2015 | AIR POLLUTION XXIII | 198 | 10.2495/AIR150181 | Ineligible outcome |
|  | Tesfaye 2023 | Spatial patterns and spatially-varying factors associated with childhood acute respiratory infection: data from Ethiopian demographic and health surveys (2005, 2011, and 2016) | 2023 | BMC Infect. Dis. | 23 | 10.1186/s12879-023-08273-1 | Ineligible outcome |
|  | Thacher 2013 | Biomass fuel use and the risk of asthma in Nigerian children | 2013 | Respir. Med. | 107 | 10.1016/j.rmed.2013.09.009 | Unspecified study population |
|  | Thorsson 2014 | Carbon monoxide concentrations in outdoor wood-fired kitchens in Ouagadougou, Burkina Faso - Implications for women's and children's health | 2014 | Environ. Monit. Assess. | 186 | 10.1007/s10661-014-3712-y | Ineligible outcome |
|  | Toe 2022 | Ambient air pollution is associated with vascular disease in Ugandan HIV-positive adolescents | 2022 | AIDS | 36 | 10.1097/QAD.0000000000003186 | Unspecified study population |
|  | Tolcos 2000 | Chronic prenatal exposure to carbon monoxide results in a reduction in tyrosine hydroxylase-immunoreactivity and an increase in choline acetyltransferase-immunoreactivity in the fetal medulla: Implications for sudden infant death syndrome | 2000 | JOURNAL OF NEUROPATHOLOGY AND EXPERIMENTAL NEUROLOGY | 59 | 10.1093/jnen/59.3.218 | Ineligible outcome |
|  | Toure 2019 | Observed and Modeled Seasonal Air Quality and Respiratory Health in Senegal During 2015 and 2016 | 2019 | GEOHEALTH | 3 | 10.1029/2019GH000214 | Ineligible outcome |
|  | Troeger 2018 | Estimates of the global, regional, and national morbidity, mortality, and aetiologies of lower respiratory infections in 195 countries, 1990â€“2016: a systematic analysis for the Global Burden of Disease Study 2016 | 2018 | Lancet Infect. Dis. | 18 | 10.1016/S1473-3099(18)30310-4 | Study setting is out of scope |
|  | Tumwesigire 1995 | Environmental risk factors for acute respiratory infections among children of military personnel in Uganda. | 1995 | East Afr Med J | 72 |  | Ineligible outcome |
|  | TUMWESIGIRE 1995 | ENVIRONMENTAL RISK-FACTORS FOR ACUTE RESPIRATORY-INFECTIONS AMONG CHILDREN OF MILITARY PERSONNEL IN UGANDA | 1995 | EAST AFRICAN MEDICAL JOURNAL | 72 |  | Study setting is out of scope |
|  | vanBree 1995 | Dose-effect models for ozone exposure: Tool for quantitative risk estimation | 1995 | TOXICOLOGY LETTERS | 82-3 | 10.1016/0378-4274(95)03484-6 | Ineligible outcome |
|  | Vanker 2014 | Indoor air pollution and tobacco smoke exposure in an African birth cohort study | 2014 | Pediatric Pulmonology | 49 | https://dx.doi.org/10.1002/ppul.23068 | Ineligible outcome |
|  | Vanker 2015 | Home environment and indoor air pollution exposure in an African birth cohort study | 2015 | Sci. Total Environ. | 536 | 10.1016/j.scitotenv.2015.06.136 | Ineligible outcome |
|  | Vanker 2017 | Early-life exposure to indoor air pollution or tobacco smoke and lower respiratory tract illness and wheezing in African infants: a longitudinal birth cohort study | 2017 | Lancet Planet. Health | 1 | 10.1016/S2542-5196(17)30134-1 | Ineligible outcome |
|  | vanNiekerk 1977 | The house-dust mite and childhood asthma in the Cape Peninsula. | 1977 | S Afr Med J | 52 |  | Ineligible outcome |
|  | Venn 2001 | Increased risk of allergy associated with the use of kerosene fuel in the home | 2001 | Am. J. Respir. Crit. Care Med. | 164 | 10.1164/ajrccm.164.9.2103101 | Ineligible outcome |
|  | Venn 2005 | Proximity of the home to roads and the risk of wheeze in an Ethiopian population | 2005 | Occup. Environ. Med. | 62 | 10.1136/oem.2004.017228 | Unspecified study population |
|  | Vesper 2008 | Higher Environmental Relative Moldiness Index (ERMIsm) values measured in Detroit homes of severely asthmatic children | 2008 | Sci. Total Environ. | 394 | 10.1016/j.scitotenv.2008.01.031 | Unspecified study population |
|  | Wafula 1987 | Diagnosis of acute respiratory infections (ARI) among under fives in the paediatric observation ward (POW), Kenyatta National Hospital, Nairobi | 1987 | East African medical journal | 64 |  | Ineligible outcome |
|  | Wafula 1990 | Indoor air pollution in a Kenyan village | 1990 | East African Medical Journal | 67 |  | Ineligible outcome |
|  | Wafula 2022 | Indoor air pollutants and respiratory outcomes among residents of an informal urban setting in Uganda: A cross-sectional study | 2022 | medRxiv |  | https://dx.doi.org/10.1101/2022.07.28.22278151 | Ineligible outcome |
|  | Wafula 2023 | Indoor air pollutants and respiratory symptoms among residents of an informal urban settlement in Uganda: A cross-sectional study | 2023 | PLoS One | 18 | 10.1371/journal.pone.0290170 | Ineligible outcome |
|  | Waller 2018 | Estimate suggests many infant deaths in sub-Saharan Africa attributable to air pollution | 2018 | Nature | 559 | 10.1038/d41586-018-05394-5 | Ineligible outcome |
|  | Weber 1999 | Risk factors for severe respiratory syncytial virus infection leading to hospital admission in children in the western region of The Gambia | 1999 | Int. J. Epidemiol. | 28 | 10.1093/ije/28.1.157 | Unspecified study population |
|  | Wedderburn 2023 | Co-trimoxazole prophylaxis for children who are HIV-exposed and uninfected: a systematic review | 2023 | J Int AIDS Soc | 26 | 10.1002/jia2.26079 | Review study |
|  | Wesley 1996 | Assessment and 2-year follow-up of some factors associated with severity of respiratory infections in early childhood | 1996 | S. AFR. MED. J. | 86 |  | Ineligible outcome |
|  | Wetsman 2018 | Air-pollution trackers seek to fill Africa's data gap news/704/172/639/638/204/675 | 2018 | Nature | 556 | https://dx.doi.org/10.1038/d41586-018-04330-x | Ineligible outcome |
|  | Wichmann 2008 | Association between children's household living conditions and eczema in the Polokwane area, South Africa | 2008 | Health Place | 14 | 10.1016/j.healthplace.2007.08.002 | Unspecified study population |
|  | WilsonChibwe 2021 | The health effects of Chunga Dumpsite on surrounding communities in Lusaka, Zambia | 2021 | medRxiv |  | 10.1101/2021.12.21.21268110 | Ineligible outcome |
|  | Wonodi 2012 | Evaluation of risk factors for severe pneumonia in children: The pneumonia etiology research for child health study | 2012 | Clin. Infect. Dis. | 54 | 10.1093/cid/cir1067 | Study setting is out of scope |
|  | Woolley 2020 | Investigating the association between wood and charcoal domestic cooking, respiratory symptoms and acute respiratory infections among children aged under 5 years in uganda: A cross-sectional analysis of the 2016 demographic and health survey | 2020 | Int. J. Environ. Res. Public Health | 17 | 10.3390/ijerph17113974 | Ineligible outcome |
|  | Woolley 2020 | Use of biomass cooking fuel and risk of respiratory symptoms and Acute Respiratory Infections in Ugandan children aged under 5 years: cross-sectional analysis | 2020 | European Respiratory Journal | 56 | https://dx.doi.org/10.1183/13993003.congress-2020.1997 | Ineligible outcome |
|  | Woolley 2022 | Association of household cooking location behaviour with acute respiratory infections among children aged under five years; a cross sectional analysis of 30 Sub-Saharan African Demographic and Health Surveys | 2022 | Atmos. Environ. | 276 | 10.1016/j.atmosenv.2022.119055 | Ineligible outcome |
|  | Wu 2005 | Development of an individual exposure model for application to the Southern California children's health study | 2005 | Atmos. Environ. | 39 | 10.1016/j.atmosenv.2004.09.061 | Study setting is out of scope |
|  | Xue 2021 | Associations between exposure to landscape fire smoke and child mortality in low-income and middle-income countries: a matched case-control study | 2021 | Lancet Planet. Health | 5 | 10.1016/S2542-5196(21)00153-4 | Unspecified study population |
|  | Yadate 2023 | Determinants of pneumonia among under-five children in Oromia region, Ethiopia: unmatched case-control study | 2023 | ARCHIVES OF PUBLIC HEALTH | 81 | 10.1186/s13690-023-01103-5 | Ineligible outcome |
|  | Yusuf 2022 | Association between Environmental Exposures and Asthma among Children in King Williams Town, South Africa | 2022 | DISEASES | 10 | 10.3390/diseases10040123 | Unspecified study population |
|  |  | Global, regional, and national comparative risk assessment of 84 behavioural, environmental and occupational, and metabolic risks or clusters of risks for 195 countries and territories, 1990-2017: a systematic analysis for the Global Burden of Disease Stu | 2018 | Lancet | 392 | 10.1016/S0140-6736(18)32225-6 | Ineligible outcome |

**Supplementary** **table 2 S4: Summary characteristics of included studies**

| **No**. | **Author(s), publication year** | **Study area, country, study year** | **Design**  **sample size(N), response rate (R%)** | **Participant characteristics (age of children)** | **Pollutant exposure or proxy exposure** | **Exposure measurement method** | **Outcome, assessment method** | **Potential confounders** | **Effect estimates (95%CI)** | **Data extractor/s, date of data extraction** | **Source of data, confirmation of eligibility** |
| --- | --- | --- | --- | --- | --- | --- | --- | --- | --- | --- | --- |
|  | Akinyemi et al., 2016 | 15 SSA countries, 2010-2014 DHS | Cross-sectional  N=143,602  R=100 | Women with their U5C categorized into infants (birth–11 months) and children (12–59 months) | 1.Maternal smoking  2. Solid fuels: coal, lignite, charcoal, wood, straw/shrubs/grass, dung, and crop residues | This information is derived from responses to two questions in the demographic and health survey (DHS) women’s questionnaire:1: Do you currently smoke cigarettes? 2: Do you currently smoke or use any other type of tobacco?” A woman who answers “yes” to either question is classified as a smoker. Additionally, the type of cooking fuel used in the household is recoded into “solid” (e.g., coal, wood) and “non-solid” (e.g., electricity, natural gas) categories. | Infant and child mortality, infant mortality defined as death between birth and 11th month of life and child mortality is death between 12th and 59 month of life and surveyed using DHS questionnaire. | Maternal age at child’s birth, maternal education, maternal occupation, household wealth index, type of residence, perceived birth size, number of birth (single or multiple), birth order and birth interval. | Infant mortality.  Smoking + solid fuel: HR: 1.59 (1.26–1.99);  no smoking + solid fuel: HR: 1.44 (1.18-1.76);  smoking + non-solid fuel: HR: 0.86 (0.44–1.68). Vs no smoking + non-solid fuel.  Child mortality.  Smoking + solid fuel: HR:1.41 (0.99–1.99); no smoking + solid fuel: HR: 1.21 (0.94–1.56);smoking + non-solid fuel: HR: 1.72 (0.71–4.17) Vs no smoking + non-solid fuel. | Eyasu Alem Lake, 2 August 2024 | Published data, Eligible |
|  | Bickton et al., 2020 | 14 SSA countries, 2015-18 DHS (spell out what DHS is first before using the acronym) | Cross-sectional  N=164376  R=100 | Women with their U5C (0-59 months) | 1.Only charcoal  2.Other biomass fuels: wood, dung, kerosene, coal, crop residues, shrubs  3. Kitchen location: in the house, separate building, outdoor | In the DHS interview mothers were asked about their cooking fuel type which was categorized as:  wood, charcoal, dung, kerosene, crop residues, shrubs, and coal.  Clean Fuels: Natural gas, biogas, liquefied petroleum gas (LPG), and electricity. | Under-five mortality (U5M), death between birth and the fifth birthday of the child and was surveyed. | Sex of the child, birth order, number of under-five children in the HH, and mother’s age at birth. | OR: 1.33 (1.03– 1.71). | Eyasu Alem Lake, 2 August 2024 | Published data, Eligible |
|  | Dano et al., 2019 | Niamey, Niger, 2015-16 | Cross-sectional  N=637  R=100 | Parents with their children (1-59 months) | Passive cigarette smoking in the house | Researchers collected data on this exposure via a questionnaire administered during confidential interviews. | Carriage of S. pneumonia, Nasopharyngeal swabbing was processed using a molecular method. SP was determined by a multiplex real-time PCR with the FTD Respiratory pathogens 21 plus. | Age (months), sex, paternal education & occupation, S. pneumoniae co-carriage with another bacteria, S.pneumoniae co-carriage with a virus, attendance at a day care centre, Flooring of the house, number of children in the house. | OR: 0.73 (95% CI: 0.47 – 1.12). | Eyasu Alem Lake, 1 August 2024 | Published data, Eligible |
|  | Egondi et al., 2018 | Nairobi, Kenya, 2003-13 | Semi-Ecological  N=21,641  R=100 | Mother with their children (below 5 years) | Outdoor particles (PM2.5) | Measured in real-time using DustTrak II 8532 hand-held samplers placed at a height of approximately 1.5 meters above ground level. These samplers logged data every minute. Sampling occurred along a fixed route within specific areas, including predefined geographical checkpoints | Child Mortality, the data was derived from nested studies within the Nairobi Urban Health and Demographic Surveillance system  (NUHDSS). | Gender, age, and socioeconomic status. | **All-cause mortality**  OR: 1.22 (95% CI: 1.08–1.39).  **Respiratory cause mortality**  (IRR=1.22; 95%CI: 0.88-1.42**).** | Eyasu Alem Lake, 5 August 2024 | Published data, Published data, Eligible |
|  | Ezeh et al., 2014 | Nigeria, 2013 NDHS | Cross-sectional  N=30,726  R=100 | Women with their U5C categorized into neonates (0-28 days of age), post-neonates (1-11 months of age), and children (12 -59 months of age) | Solid fuels: coal, charcoal, wood, agricultural crop, animal dung, straw/shrubs/grass | 1. Exposure to solid fuel was determined by asking respondents about their household’s main cooking fuel during DHS interview. Responses categorized as:solid fuels: coal, lignite, charcoal, wood, straw, shrubs, grass, agricultural crop, and animal dung; 2. nonsolid fuels: electricity, liquefied petroleum gas (LPG), natural gas, biogas, and kerosene. | Neonatal mortality (death between birth-28 days), post-neonatal mortality (1-11 months) and child mortality (12-59 months). | Sex, mother’s (education, working status and age at the birth of the child, perception of her newborn’s size at birth), and breastfeeding. | Neonatal mortality  HR: 1.01 (0.73–1.26).  Post-neonatal mortality: HR:1.92 (1.42–2.58),  Child mortality:  HR: 1.63, (1.09–2.42). | Eyasu Alem Lake, 1August 2024 | Published data, Eligible |
|  | Fakunle et al., 2014 | Ibadan, Nigeria, 2012 | Case-control  N=220 (cases)  N=220 (controls)  R=100 | Mother with their U5C (below 5 years) | 1. Firewood for cooking  2. Lantern smoke  3.Smoking in the house  4.Carrying the child while cooking | Collected from mother using interview questionnaire. | Acute respiratory infection (ARI). World health organization (WHO) definition for ARI used. | Child age, gender, and maternal education, number of 5 persons in household, previous and family history of ARI, parental smoking¸ use of mosquito coils, keeping pets/livestock, use of lantern at night. | OR 9.3 (3.6–  24.1). | Eyasu Alem Lake, 3 August 2024 | Published data, Eligible |
|  | Flanagan et al., 2022 | Adama, Ethiopia, 2015-18 | Prospective Cohort  N=2085  R=100 | Pregnant women (0-30 days) | 1.Ambient NO2 and NOx  2. Solid fuel: wood, charcoal, cow dung  3.Mixed fuel: solid and clean (electricity, gas/kerosene, LPG) fuels. | Exposure to ambient air pollution and indoor air pollution, nitrogen oxides (NOX) and nitrogen dioxide (NO2) were measured at over 40 sites during wet and dry seasons (for six days each). Land-use regression (LUR) models were developed for NO_X_ and NO_2_ for Adama. NO_2_ and NO_X_ data was collected with a Thermo Scientific NO-NO2NOX analyzer (model 42i) at the Ethiopian Meteorological Institute site. Cooking fuel categories were categorized to: “clean fuel” (electricity, gas/kerosene, and, “solid fuel” (wood/charcoal and cow dung only), and “mixed fuel types” using questionnaire. | Neonatal death (deaths occurring within the first 30 days after birth). Self-reported questionnaire during the participant’s postnatal visit or by phone if an in-person meeting was not possible. | Maternal age, education, parity, and Human Immune Deficiency Virus (HIV) status. | NOx:(OR: 0.72; (95% CI: 0.39–1.36), NO_2_( OR: 0.91; (95% CI: 0.32–2.58), Clean vs other fuel type†(AOR: 0.61; (95% CI: 0.20–1.85). | Eyasu Alem Lake, 3 August 2024 | Published data, Eligible |
|  | Francisco et al., 1993 | Rural Gambia, 1990 | Cases and control  N=129(cases)  N=144(dead controls)  N=270(live controls)  R=100 | Mother/guardian their child (less than 2 years | 1.Parental smoking  2. Mother carried child while cooking  3. indoor air pollution | A detailed questionnaire which covered potential risk factors for death from acute lower respiratory infection (ALRI) was administered to the families of cases and controls as soon as possible after the death of a case. | ALRI death, deaths in children aged < 5 years are recorded and cause of death was confirmed by three physicians and was accepted when at least two physicians agree on the diagnosis. | Vitamin A intake, ANC visit, weight for age, immunization status, child ever visited welfare clinic, child treated with western medicine with the last illness. | Mother carried child while cooking (never vs always)  OR: 5.23 (1.72-15.92).  Parental smoking (none vs both)  OR: 3.67 (1.09-12.42). | Eyasu Alem Lake, 12 August 2024 | Published data, Eligible |
|  | Heathfield et al., 2020 | Cape Town, South Africa, 2013-17 | Retrospective document review  N=1608 | Medical records with infant death (below 1 years) | Passive smoking from mothers or other family members in house | The data collected from the medico-legal case folders retrieved from the archives. | Sudden unexpected infant death (SUID), all infant death cases admitted for forensic investigation were reviewed, including those thought to be due to unnatural causes (2013 South African Health Act No. 16) . | Co-slept in an adult bed, HIV exposed, premature infants, breastfed, missed antenatal appointments, drinking alcohol and drug use. |  | Eyasu Alem Lake, 10 August 2024 | Published data, Eligible |
|  | Imo et al., 2023 | Nigeria, 2018 NDHS | Cross-sectional  N=124,442  R=100 | Women with U5C (0-5 years) | Solid fuel: coal/lignite, wood, charcoal, straw/shrubs/ grass, agricultural crops, animal dung | Using Nigerian demographic and health survey (NDHS) the author categorized what type fuel used for cooking into: solid (coal/lignite, charcoal, wood, straw/shrubs/grass, agricultural crops and animal dung) and non-solid (electricity, gas and kerosene) fuels. | Under-five mortality, defined as the risk of a live-born child dying between birth and their fifth birthday (0–59 months), measured as the duration of survival since birth in months and surveyed using DHS questionnaire. | Maternal age, mother’s and father’s educational attainment, mother’s and father’s employment status, place of residence and region. | HR: 2.26 (2.06–2.49) | Eyasu Alem Lake, 16 August 2024 | Published data, Eligible |
|  | Johnson et al., 1992 | Ibadan, Nigeria, 1985-86 | Case-control  N=103(cases)  N=103(control)  R=100 | Mother/guardian their child (2 weeks - 59 months) | Household pollutants | The information through interview obtained regarding domestic variables encompassed the smoking habits of parents and close neighbors, the type of kitchen fuel used, the location of cooking areas, parental occupation and income, and the number of individuals residing in the household. | Outcome of ARI hospitalization, ALRI syndrome definition of Denny and Clyde were  used. |  |  | Eyasu Alem Lake, 9 August 2024 | Published data, Eligible |
|  | Johnson et al., 2008 | Nigeria, a 30-month follow-up study (no study year found) | Prospective study  N=419  R=100 | Mother/guardian their child (2 weeks - 59 months) | 1.Cooking fuels: Kerosene +gas, wood + kerosene  2. Kitchen location: inside, corridor, separate  3. Smoker in the house | Clinical data, including respiratory illness risk factors like domestic smoke exposure, immunization status, feeding practices, and parental socioeconomic details, were recorded in a pre-coded questionnaire. | Community acquired pneumonia (CAP) associated mortality, a lower respiratory infection that satisfied the ALRI syndrome definition of Denny and Clyde were included. | Admission Season, maternal education, social class, cigarette smoker (s) in the house, crowding. | OR:2.92 (1.18–6.87) | Eyasu Alem Lake, 9 August 2024 | Published data, Eligible |
|  | Kiconco et al., 2021 | Kampala, Uganda, 2019 | Cross sectional  N=336  R=100 | Parents/caretakers with their children (2 -59 months) | 1.Place of cooking  2. Parental smoking | A structured questionnaire captured detailed data on acute respiratory symptoms, socio-demographics, breastfeeding history, birth weight, immunization status, environmental factors, and medical conditions. | Pneumonia, presence cough and/or difficulty in breathing with fast breathing and/or chest in drawing and the definition was modified by presence of positive chest X-ray findings of pneumonia. | Age of the child, sex, marital status, residence, parental education, religion, tribe, immunization status, nutritional status, exclusive breast feeding and comorbidities. | Place of cooking (indoor vs outdoor):  OR: 0.9 (0.54-1.53).  Parental/caretaker smoking:  OR:3.0 (1.35-6.80). | Eyasu Alem Lake, 8 August 2024 | Published data, Eligible |
|  | Kleimola et al., 2015 | 27 SSA countries, 2005-12 DHS | Cross sectional  N=418622(neonatal) N=404254(under-five)  R=100 | Women with their U5C categorized into neonates (birth–28days), infants and/ or children (29 days to 59 months) | 1. Solid fuels: coal, charcoal, biomass like wood, crop waste and dung  2. Kerosene | Using DHS data the exposure of interest was categorized into: clean fuels (electricity, liquid petroleum gas, natural gas, and biogas), kerosene, and solid fuels (coal, charcoal, and biomass such as wood, crop waste, and dung). | Neonatal and child mortality, in the DSH the omen’s questionnaire collects a birth history including information on age at death of all liveborn children (stillbirths are not included in the birth history). | Sex, birth order, mother’s education, mother’s age at birth, whether she currently smokes cigarettes, household wealth index, urban versus rural residence, and country. | Using solid fuels vs clean fuels:  neonatal mortality  RR: 1·06 (0·92, 1·21).  Child Mortality  RR: 1·02 (0·92, 1·13).  Using kerosene vs clean fuels; neonatal mortality  RR: 1.22 (1·01, 1·48).  Child mortality:  RR: 0·91 (0·78, 1·07). | Eyasu Alem Lake, 8 August 2024 | Published data, Eligible |
|  | Latona et al., 2017 | Nigeria, 2008 NDHS | Cross-sectional  N=28647  R=100 | Women with their U5C (0–5 years) | Biomass cooking fuels | This information was derived from NDHS questionnaire. | Child Mortality, self-report from mothers/respondents. | Residence, religion, wealth Index, household size, availability of toilet facility, condition of floor, cooking fuel material, size of baby at birth, preceding birth interval, postnatal visit within two months. | HR = 0.448(0.354 – 0.567) | Eyasu Alem Lake, 8 August 2024 | Published data, Eligible |
|  | Nantanda et al., 2013 | Kampala, Uganda, 2011-12 | Cross sectional  N=614  R=100 | Caretakers with their children (2 -59 months) | 1.Tobacco smoking  2.Gas use for cooking | A pretested questionnaire in English which translated to local language Luganda were used to collect the information. | **Asthma,** the modified version of GINA (Global Initiative for Asthma) guidelines was used for diagnosis.  **Bronchiolitis:** the case definition of bronchiolitis was based on South African guidelines for diagnosis, management and prevention of acute viral bronchiolitis. | Maternal asthma, history of allergy in patient, gender, exposure to tobacco smoke, prematurity, exclusive breastfeeding for at least 3 months, education level of caretaker. | **Asthma**  Use of gas for cooking  OR: 3.8 (1.2, 13.3)  Exposure to tobacco smoke:  OR: 1.5 (0.8–2.7)  **Bronchiolitis**  Use of gas for cooking OR:1.4 (0.5–4.5)  Exposure to tobacco smoke OR:1.3 (0.7–2.3) | Eyasu Alem Lake, 8 August 2024 | Published data, Eligible |
|  | Ngocho et al., 2019 | Kilimanjaro, Tanzania, 2017 | Case control  N=113 (cases)  N=350 (control)  R=100 | Parents with their U5C (2–59 months) | 1.Unclean cooking fuels: kerosene, biomass, firewood, charcoal  2. Smoking in the house members | Families using gas or electricity to cook were considered to use clean cooking fuel, while biomass, firewood, charcoal and kerosene were considered unclean and all the recorded information obtained through interviewee using questionnaire. | Pneumonia, hospitalized children aged 2–59 months who met the WHO case definition of pneumonia with X-ray-confirmed pneumonia were included. | Age in moth, sex, birth weight, exclusive breastfeeding, completed immunization status, prior antibiotics/zinc tablets, weight for age Z-score( WAZ), income activities, other children under 5 years of age at home, parent/guardian education, cigarette smoking. | OR:1.7 (1.0–2.8) | Eyasu Alem Lake, 8 August 2024 | Published data, Eligible |
|  | Owili et al., 2017 | 23 SSA countries, 2010-14 DHS | Cross sectional  N=783,691  R=100 | Women with their U5C (0–5 years) | 1.Charcoal  2. Other biomass: wood, agricultural crops, dung, straw/shrubs/grass  3.Other pollutant cooking fuel: coal, lignite, paraffin/kerosene  4.Kitchne location  5. Smoking in the house | The information was derived from DHS data and classified into: ‘clean’ fuel (electricity, natural gas, biogas or liquefied petroleum gas) as the reference group; biomass cooking fuels divided as ‘charcoal’ (i.e. majorly used indoors) and other ‘biomass’ fuels – mainly used outdoors for cooking (wood, straw/shrubs/grass, agricultural crops, or animal dung); and ‘other’ pollutant cooking fuel (coal, lignite or paraffin/kerosene). | Under-five mortality, all-cause mortality of the under-five with time-to-event being age in months was used. The survey had a question asking whether the child was alive or dead at the time of the interview. | Country, residence and administrative provinces, child’s sex, breastfeeding status number of under-5 children in household (HH), mother’s age and family size, wealth index, mother’s education, mother’s and father’s occupation, members smoking in HH. | HR: 1.21(1.10–1.34) and 1.20 (1.08–1.32) for charcoal and biomass cooking fuel respectively compared to clean fuels. | Eyasu Alem Lake, 10 August 2024 | Published data, Eligible |
|  | PrayGod et al., 2016 | Mwanza, Tanzania, 2013-14 | Case-control  N=42 (cases)  N=72 (controls)  R=100 | Parents/guardians with their children (2–59 months) | 1.Cooking fuel: firewood, wood charcoal  2.Smoking  3.Outdoor cooking | The author used structured questionnaire to collect data on cooking fuel sources (electricity, gas, wood charcoal, or firewood) and indoor/outdoor food preparation to investigate indoor air pollution's role in severe pneumonia risk. They also determined parental smoking status through structured questionnaires. | Severe pneumonia, based on WHO definitions if they had fast breathing (>50 breaths per minute for those aged 2 to 11 months and >40 breaths for those aged 12 to 59 months). | Age, sex, measles vaccination, vitamin A supplementation in the past 6 months, received antibiotics before referral, Enterobacter sp. | OR:5.5 (1.4, 22.1) | Eyasu Alem Lake, 10 August 2024 | Published data, Eligible |
|  | Roux et al., 2015 | Paarl,  South Africa | Cohort  N=1000  R=69.7 | Mother-infant pairs to 1 year of age | Maternal smoking | The author uses a structured questionnaire to obtain environmental data on follow-up visits from the mother. | Pneumonia: WHO case definition cough/difficulty breathing and tachypnea (≥50 breaths/min for 2–12 months) or chest indrawing. Severe pneumonia was diagnosed with tachypnea (>60 breaths/min for <2 months), chest indrawing, or danger signs (cyanosis, seizures, inability to drink, reduced consciousness). | Study site, season, maternal education sex, child’s HIV exposure, child age, birth weight, feeding status, vaccination, MUAC. | OR=1.94 (1.32-2.28) | Eyasu Alem Lake, 10 August 2024 | Published data, Eligible |
|  | Roux et al., 2021 | Drakenstein,  South Africa | Cohort  N=1143  R=100 | Mother-child pairs | 1. Indoor PM10  2.Maternal smoking | PM10 was measured over 24 hours using an air pump, with a standard of 40 μg/m³. Toluene levels were monitored for 2 weeks using diffusion tubes. Maternal smoking was determined by urine cotinine levels, with <500 ng/mL indicating non/passive smokers and >500 ng/mL for active smokers. | Pneumonia: WHO case definition cough/difficulty breathing and tachypnea (≥50 breaths/min for 2–12 months) or chest indrawing. Severe pneumonia was diagnosed with tachypnea (>60 breaths/min for <2 months), chest indrawing, or danger signs (cyanosis, seizures, inability to drink, reduced consciousness). | Birth characteristics, WHO danger signs, clinical conditions and chest radiograph result. | OR:3.17 (0.38–26.46)  Exposure to indoor PM_10_  OR:1.33 (0.45-3.95)  Exposure to maternal smoking. | Eyasu Alem Lake, 10 August 2024 | Published data, Eligible |
|  | Samuel et al., 2018 | Nigeria, 2013 NDHS | Cross-sectional  N=10,983  R=100 | Women with their U5C(0-59 months) | 1.Solid fuel: wood, dung, charcoal  2. Location of kitchen inside the house | The information was derived from NDHS data which had asked the use of solid fuels for cooking and kitchens location in homes. | Under-five mortality, the respective mothers self-report during NDHS interview about the survival status of their under-five child. | Wealth, education, residence, region. | OR:1.23.(0.98-1.54) | Eyasu Alem Lake, 1 August 2024 | Published data, Eligible |
|  | Shifa et al., 2018 | Gamo Gofa, Ethiopia, 2011-14 | Case control  N=381 (cases)  N=762 (controls)  R=100 | Under-five children (below five years) | 1.Lack separate kitchen in household  2.Light source in the household | A pre-tested closed-ended structured Amharic questionnaire was utilized for data collection. | Infant and under five mortality, the probability of dying between  birth and the first birthday and the probability of dying before the fifth birthday, respectively. | Sex, mother’s education, wealth index, husband occupation and marital status of the mother. | Under-five mortality  OR:1.77(1.16–2.70)  Infant mortality  OR: 1.94(1.13 3.33) | Eyasu Alem Lake, 11August 2024 | Published data, Eligible |
|  | Shiferaw et al., 2023 | Ethiopia, 2016 EDHS | Cross-sectional  N=10452  R=100 | Women with their U5C (below 5 years) | Ambient PM_2_  _.5_ | The data source from ambient PM2.5 was derived from a global study by the Atmospheric Composition Analysis Group(ACAG) at Washington and Dalhousie University in the United States and Canada, respectively and it was a satellite based PM2.5 estimate 0.001º×0.001º spatial resolution. | Under-five mortality: death before the age of  60 months and obtained from Ethiopian demographic and health survey (EDHS). | Mother’s education, wealth, family size, child sex, size of the child at birth, child age, the plurality of child, place of delivery, the place where food is cooked, residence. | OR: 2.29 (1.44,  3.65) | Eyasu Alem Lake, 12 August 2024 | Published data, Eligible |
|  | Starnes et al., 2023 | Migori County, Kenya, 2021 | Cross-sectional  N=15,999  R=100 | Household living U5C (below 5 years) | Indoor smoking | Household heads aged 18 or older were surveyed. Exposure to indoor smoking was delineated as the presence of in-house cooking stove which lacked ventilation during the data collection time. | Under-five mortality: defined as in the study the occurrence of reported deaths before celebrating the fifth birthday. | Year birth, sex, maternal age, parents currently married/in relationship, mother with secondary  or more education, birth order, wealth, birth spacing ≤18 months, born during long rain season, household region. Visited by community health workers (CHW) in the last 3 months. | HR: 1.916 (1.075 - 3.415) | Eyasu Alem Lake, 14 August 2024 | Published data, Eligible |
|  | Tazinya et al., 2018 | Bamenda, Cameroon, 2014-15 | Cross-sectional  =512  R=100 | Parents/guardians with their children (2 -59 months) | 1.Wood smoke  2.Passive smoking | Data was gathered via a structured questionnaire covering demographic, clinical, and socioeconomic variables of the child and guardians. Wood smoke exposure was defined as spending over 30 minutes daily in such an environment. Passive smoking referred to any child living with a smoker at home. | ARI, case definition based on the Integrated Management of Childhood Illnesses (IMCI) classification for children presenting with cough or difficulty breathing.: mild ARI (no pneumonia), moderate ARI (pneumonia) and severe ARI (severe pneumonia). | Age, sex, birth weight, co-infection with HIV, immunization status, breastfeeding, nutritional status, parental education, parents’ age, school attendance and overcrowding, HIV status, tendency at day care. | Exposure to wood smoke (not vs exposed):  OR:1.85(1.22–2.78)  Passive smoking (no vs yes)  OR:3.58 (1.45–8.84) | Eyasu Alem Lake, 14 August 2024 | Published data, Eligible |
|  | Ujunwa et al., 2014 | Enugu, Nigeria, 2007-08 | Cross-sectional  N=436  R=100 | Parents/caregivers with their children (below five years) | 1. Wood biofuel  2. Passive smoking | A structured pro forma collected socio-demographic characteristics. Any history of either parent smoking at home was noted as partial smoking exposure | ARI, Case definition was according to WHO working group on case management of ARIs, which defined ARI as a clinical state presenting with rapid breathing more than expected upper limit for age with or without chest in drawing, too sick to feed, nasal discharge, cough, fever with or without auscultatory findings of less than 2 weeks. | Age, malnutrition, breastfeeding, residence, maternal/paternal education, family size, attendance to day care centre, immunization status. | Pneumonia:  Passive smoking: RR:1.39 (1.05-1.83);  wood biofuel  RR: 2.09 (1.39-3.14).  Bronchitis:  Passive smoking:RR: 0.35 (0.1262-0.99); wood biofuel  RR: 1.09 (0.5-2.39).  AURI:  Passive smoking: RR: 0.91 (0.76-1.08); wood biofuel  RR: 0.74 (0.64-0.85. | Eyasu Alem Lake, 2 August 2024 | Published data, Eligible |
|  | Vanker et al., 2017 | Paarl, South Africa, 2013-15 | Longitudinal  N=1137 mothers with 1143 live births  R=90 | Mothers and their live-born children were followed for three years from birth. | 1.PM_10_  2. CO  3. Nitrogen dioxide, sulfur dioxide, and volatile organic compounds (benzene and toluene) | PM_10_ was measured with an AirChek 52 pump, and carbon monoxide was monitored using an Altair detector, both left in homes for 24 hours. NO_2_ and SO_2_ were measured with Radiello filters, while benzene and toluene were assessed with thermal desorption tubes, all placed for two weeks. | LRTI: defined by WHO criteria. Active surveillance included LRTI occurring at or shortly after birth. Wheeze episodes were self-reported by caregivers or diagnosed by trained staff during study visits. Both groups were trained in wheezing recognition. Recurrent wheezing was defined as two or more episodes. Smoking was assessed using a questionnaire. | Socioeconomic quartile, infant characteristics (sex, WAZ at birth, age), maternal HIV exposure | Maternal smoking:  IRR 1.62: p=0.004  PM_10:_  IRR: 1.43, p=0.008  OR 5.13, p=0.012 | Eyasu Alem Lake, 11 August 2024 | Published data, Eligible |
|  | Verani et al., 2016 | Soweto, South Africa,2010-12 | Case control N=889(cases)  N=2628(control)  R=100 | Parents/guardians with their children (below 5 years) | Secondhand smoking | Nurses conducted in-person interviews with the parent/guardian of enrolled cases and controls to collect data on the child's medical history, demographics, and household information. | Presumed bacterial pneumonia (PBP), HIV uninfected children with lower respiratory tract infection and consolidation on chest radiograph or nonconsolidated infiltrate with C-reactive protein ≥40mg/L. | Race, sex, malnutrition, heart disease, previous pneumonia hospitalization, mother HIV-infected, exclusive breastfeeding up to age 4 months, water source indoor tap, crowding. | OR: 5.15 (2.94–9.03) | Eyasu Alem Lake, 7 August 2024 | Published data, Eligible |
|  | Wichmann et al., 2006 | South Africa, 1998 SADHS | Cross-sectional  N=3556  R=100 | Women with U5CU (1-59 months) | 1. Polluting fuels: wood, dung, coal, paraffin, and combination with clean fuels (LPG, natural gas, electricity) | The 1998 SDHS asked participants on fuel types and categorized responses as: polluting fuels (wood, dung, coal, or paraffin without LPG/natural gas or electricity) and clean fuels (exclusively LPG/natural gas or electricity). | Under five mortality: outcome variable was ascertained by asking a woman whether she had given birth to any children and whether any of them were still alive and, if not, at what age the child, or children had passed away. | Age, sex, birth order, mother’s age at birth, preceding birth interval, breastfed, access to flush toilet, water source, asset index, family size, year of birth. | OR: 1.99 (1.04, 3.68) | Eyasu Alem Lake, 9 August 2024 | Published data, Eligible |
|  | Winterbach et al., 2021 | Western cape province, South Africa, 2012-16 | Retrospective  N=440  R=100 | Archives with Sudden unexpected death of infants (SUDI) admission (below one year) | Cigarette smoke | A data collection form was devised to capture various variables and information was obtained from original case files stored in mortuary archives or their electronic counterparts on Livelink (Open Text, Canada). | SUDI and data collection form for numerous variables including demographic information, circumstances surrounding death, clinical history and autopsy findings and risk factors for SUDI. Data were collected from original case files in the archives of each mortuary and/or from their electronic equivalents stored on Livelink (Open Text, Canada). | Low birthweight, prematurity, maternal alcohol use in pregnancy, bed sharing. |  | Eyasu Alem Lake, 6 August 2024 | Published data, Eligible |

**Footnote**: Efforts were made to identify and retrieve any missing data through a thorough search of relevant databases and by contacting the authors of studies with missing data. However, no additional data were received. Studies with critical missing data, such as the review's primary and secondary outcomes or exposure of interest, were excluded from the review. All included studies had fully available data necessary for the analysis.
