# Supplementary material for: The effect of air pollution on morbidity and mortality among children aged under five in sub-Saharan Africa: Systematic review and meta-analysis
Source: PLoS One. 2025 Apr 10;20(4):e0320048. doi: 10.1371/journal.pone.0320048 (PMC11984980; doi:10.1371/journal.pone.0320048)
Supplement: S5 File — (DOCX) [file pone.0320048.s005.docx]

## **Supplementary file S**5: Risk of bias assessment

**Supplementary table1 S5: Quality assessment tool used to rate eligible studies (adapted from the OHAT for systematic review and meta-analysis)**

| **Bias** | **Risk of bias (question/domains)** | **Type of risk** |
| --- | --- | --- |
| **Key criteria** |  |  |
| **Recruitment strategy** | **Was the strategy for recruiting participants consistent across study groups?** | **Low:** Recruitment, and inclusion/exclusion were applied similarly across groups and any one of the following:   - Study participants were recruited from the same population at the same time frame; or - Study participants were not all recruited from the same population, but proportions of participants from each population in each study group are uniform.   **Probably low:** There is insufficient information about participant selection to permit a judgment of ‘low’, but there is indirect evidence that suggests that participant recruitment and inclusion/exclusion criteria was consistent, as described by the criteria for a judgment of ‘low’.  **Probability high:** There is insufficient information but indirect evidence that suggests that participant recruitment or inclusion/exclusion criteria was inconsistent.  **High:** Any one of the following:   - Protocols for recruitment or inclusion/exclusion criteria were applied differently across study groups; or - Study participants were recruited at different time frames; or - Study participants were recruited from different populations and proportions of participants from each population in each study group are not uniform.   **Not applicable:** participant selection is not an element of study design capable of introducing risk of bias in the study. |
| Exposure assessment | Was measurement assessment robust? | **Low:** The reviewers judge that there is low risk of exposure misclassification if:   - Direct measurement of air pollution concentrations in indoor air using a validated measurement device or, - Direct independent observation of the presence of specific indoor sources (e.g., stoves or heaters) or, - Assignment of outdoor (ambient) exposure using either   - Land use regression model fitted to locally acquired data from air pollution monitors and predictor data, or   - Validated emissions-dispersion (chemical transport) model fitted to locally acquired and valid emissions inventory - Independent and validated data on proximity and intensity of exposure to outdoor sources of air pollution (including traffic, power stations, industry).   **Probably low:** There is indirect evidence to judge the low risk of bias with uncertainty about the validation of the measurement.  **Probably high:** There is indirect evidence that the exposure was assessed using poorly validated methods including:Questionnaire (self-reported) data on exposure to indoor (stoves and heaters) and outdoor (traffic, power stations, industry) sources.  **High:** There is direct evidence that the exposure was assessed using methods with poor validity (all other assessments methods) OR There is low confidence in the accuracy of the exposure assessment methods including assessment method not described in sufficient detail to classify as above. |
| **Outcome assessment** | Outcome assessment methods are accurate. | **Low:** Under-five mortality- the data taken from well functioned vital registration system and the information was grasped by trained data collectors but not by researcher   - Hospitalization is assessed using standardized predefined or diagnostic investigation prospectively by physician or trained health worker at same time frame in both groups and those assessors including study subjects are adequately blinded about to the exposure level or unlikely to broken blinding before reporting the outcome.   **Probably low:** Under-five mortality- is taken from population census, household survey, demographic surveillance sites, direct parent/guardian interview and the data collector is not the researcher   - Hospitalization assessed retrospectively using standardized predefined or diagnostic investigation by trained assessor at same time frame in both groups.   OR   - Indirect evidence that outcome assessed using standard criteria but collected by researcher.   **Probably high:** There is under-five mortality data taken from population census, household survey, demographic surveillance sites, direct parent/guardian interview and the data collector is the researcher.  There is indirect evidence that hospitalization assessed using non-standard criteria such as non-validated questionnaire accompanied with insufficient information about blinding of outcome assessors and/or method is not reported.  **High:** The study did not report information of outcome measurement and there is no any evidence for outcome measurement.  There is direct evidence of hospitalization was assessed based on self-reports (parents, family) and data collected by the researcher or no any evidence about hospitalization assessment. |
| **Confounding bias** | Study properly included all important confounders | **Low:** the study accounted for (i.e., matched, stratified, multivariate analysis or otherwise statistically controlled for) important potential confounders or reported that the potential confounders were evaluated and omitted because inclusion did not substantially affect the results.  **Probably low**: the study accounted for most but not all of the important confounders this lack of accounting is not expected to introduce substantial bias  **Probably high**: the study accounted for some but not all of the important potential confounders AND this lack of accounting may have introduced substantial bias. Or, there is likely unmeasured or uncontrolled confounding based on the included confounders, and their measurement and/or strategy of evaluating confounding is unclear.  **High**: did not account for potential confounders OR there was no adjustment for confounding.  **Not applicable**: confounding cannot introduce bias. |
| **Incomplete outcome data** | Was addressed incomplete data adequately | **Low**: Any one of the following:   1. No missing outcome data; or 2. Reasons for missing outcome data unlikely to be related to true outcome (for survival data, censoring unlikely to introduce bias); or 3. Attrition or missing outcome data balanced in numbers across exposure groups, with similar reasons for missing data across groups; or 4. The proportion of missing outcomes compared with observed event risk not enough to have a relevant impact on the intervention effect estimate; or 5. Missing data have been imputed using appropriate methods.   **Probably low:** There was inadequate information on incomplete data to judge for low risk, but indirect evidence proposes the lack of incomplete data.  **Probably high:** There was insufficient information to judge a high risk of bias but indirect evidence suggests the presence of incomplete data.  **High:** Any one of the following:   - Reason for missing outcome data likely to be related to true outcome, with either imbalance in numbers or reasons for missing data across exposure groups; or - The proportion of missing outcomes compared with observed event risk enough to induce biologically relevant bias in intervention effect estimate;   Or   - Potentially inappropriate application of imputation.   **Not applicable:** The presence of incomplete data did not introduce bias. |
| **Selective reporting** | Selective reporting of outcomes or analyses. | **Low**: All the study’s pre-specified (primary and secondary) outcomes outlined in the protocol, methods, abstract, and/or introduction that are of interest in the review have been reported in the pre-specified way.  **Probably low**: insufficient information available regarding selective reporting to judge low risk of bias but indirect evidence indicate that the study is free of selective reporting.  **Probably high:** : insufficient information available regarding selective reporting to judge high risk of bias but indirect evidence indicate that the study is not free from selective reporting.  **High**:  Any one of the following:   - Not all of the study’s pre-specified primary outcomes (as outlined in the protocol, methods, abstract, and/or introduction) have been reported; or - One or more primary outcomes is reported using measurements, analysis methods or subsets of the data (e.g., subscales) that were not pre-specified; or - One or more reported primary outcomes were not pre-specified (unless clear justification for their reporting is provided, such as an unexpected effect); or - One or more outcomes of interest are reported incompletely.   **Not applicable:** there is evidence that selective outcome reporting is not introducing bias in the study |
| **Conflict of interest** | Sources of funding | **Low:** study did not receive any funding and no financial interest.  **Probably low:** information for low risk of bias judgment was inadequate but evidence from indirect sources showed lack of financial interest.  **Probably high:** information for high risk of bias judgment was inadequate but evidence from indirect sources showed the presence of financial interest.  **High:** study received funding from an entity with financial interest. |
| **Other sources of bias** | Bias caused by other sources which are not covered. | **Low:** no other sources of bias.  **Probably low**: there is inadequate information to judge for low risk, but indirect evidence proposes the lack of other sources of bias.  **Probably high:** there is inadequate information to judge for high risk, but indirect evidence proposes the presence of other sources of bias.  **High:** The presence of at least one critical source of bias. |

**Supplementary table2 S5: Results of risk of bias assessment conducted for each study**

| **#1: Akinyemi et al., 2016** | | |
| --- | --- | --- |
| **Design** | **Cross-sectional** | |
| **Participant** | Household with under-five children | |
| **Exposure** | Exposure to maternal smoking and solid fuel use | |
| **Outcome** | Infant and child mortality | |
| **Risk assessment** | **Supportive response** | **Author’s judgment** |
| Recruitment strategy | The study used DHS data. Households were usually selected through stratified two-stage cluster sampling with census enumeration areas as primary sampling units. Men aged 15–59 years and women aged 15–49 years in sampled households were then interviewed by trained field workers. The study selection process was independent to exposure or outcome status and so there is low bias in the selection of participants. | Low |
| Exposure assessment | The DHS used a standardized questionnaire via interview of the household head or his/her representative to collect data including type of cooking fuel. The study also categorized solid (coal, lignite, charcoal, wood, straw/shrubs/grass, agricultural crop and animal dung) and non-solid (electricity, LPG, natural gas, biogas, kerosene) cooking fuel. The study is susceptible to recall bias due to the nature of the data acquisition measurement employed, and it would be better to complement this method with direct observation of the fuels. Additionally, there was a lack of information on how households were categorized when mixed fuels were used. | Probably High |
| Outcome assessment | Infant mortality defined as death between birth-11 months and child mortality was categorized as death between 12-59 months and the data collected using the DHS questionnaire. The data was obtained through self-report from respondents. Despite being a major event there might be potential for recall bias or inaccuracy in reporting. | Probably Low |
| Confounding bias | The author adjusted for maternal age at child’s birth, maternal education, maternal occupation, household wealth index, type of residence, perceived birth size, number of births (single or multiple), birth order and birth interval. The study noted that they did not control for other sources of environmental pollution or other potentially important confounding variables such as birthweight, vaccination status, breastfeeding status, malnutrition, overcrowding condition, safety of drinking water, region, or other existing disease conditions. | Probably high |
| Incomplete outcome data | Sensitivity analyses were conducted to investigate the role of birthweight (missing data). Non-inclusion of birthweight in the models resulted in the risk of childhood mortality associated with maternal smoking and solid fuel being underestimated, which is a known concern/ (which has been previously reported in other literature). | Probably Low |
| Selective reporting | All the study’s specified outcomes were adequately reported. | Low |
| Conflict of interest | The authors declared no competing interests. There is no evidence of financial conflict of interest. However, it should be noted that the absence of a means to declare bias due to financial interest does not rule out the potential for such bias | Probably Low |
| Other sources of bias | Cause effect association may have been underestimated due to the cross-sectional nature of the data. All-cause mortality may include mortality outcomes that were not associated to maternal smoking or solid fuel use. | Probably Low |

| **#2: Bickton et al., 2020** | | |
| --- | --- | --- |
| **Design** | **Cross-sectional** | |
| **Participant** | Under-five yr children | |
| **Exposure** | Exposure to biomass fuel for cooking | |
| **Outcome** | Under-five all-cause mortality | |
| **Risk assessment** | **Supportive response** | **Author’s judgment** |
| Recruitment strategy | The study used DHS data. Households were selected through stratified two-stage cluster sampling with census enumeration areas as primary sampling units. Men aged 15–59 years and women aged 15–49 years in sampled households were then interviewed by trained field workers. The authors study selection process was independent to exposure or outcome status and so there is low bias in the selection of participants. | Low |
| Exposure assessment | Exposure to HAP as the use of biomass fuels for cooking. Exposure to ‘HAP’ defined as the use of biomass fuels for cooking. During the DHS interviews, mothers were asked the question “What type of cooking fuel do you use?” and researchers categorized responses into exposed (wood, charcoal, dung, kerosene, crop residues, shrubs, and coal) and clean fuels (natural gas, biogas, liquefied petroleum gas (LPG) and electricity). The study is susceptible to recall bias due to the nature of the data acquisition measurement employed, and it would be better to complement this method with direct observation of the fuels. Additionally, there is a lack of information on how to categorize for mixed users. | Probably High |
| Outcome assessment | Under-five mortality, death between birth-5 yrs. The data was obtained through self-report from respondents, despite there being potential for recall bias or inaccuracy in reporting. | Probably Low |
| Confounding bias | The study adjusted for the following potential confounders: sex, birth order, number of under-five children in the household, and mother’s age at birth, mother’s education level (none, primary, secondary, higher), occupation, residence (urban/rural) and wealth index. The study did not adjust for other important confounding variables such as birthweight, vaccination status, breastfeeding status, malnutrition, safety of drinking water, region, or other existing disease conditions. | Probably high |
| Incomplete outcome data | The study used missing indicator method by creating an indicator variable for those with missing data on kitchen location and smoking frequency in the household. | Probably Low |
| Selective reporting | All of the study’s specified outcomes were adequately reported. | Low |
| Conflict of interest | This study had no funding source. | Low |
| Other sources of bias | Causal association may have been underestimated due to the cross-sectional nature of the data. The all-cause mortality may include mortality outcomes that were not associated to maternal smoking and solid fuel use. | Probably Low |

| **# 3: Dano et al., 2019** | | |
| --- | --- | --- |
| **Design** | **Cross-sectional** | |
| **Participant** | Children aged 1 to 59 months | |
| **Exposure** | Passive cigarette smoking in the house | |
| **Outcome** | Carriage of S. pneumonia | |
| **Risk assessment** | **Supportive response** | **Author’s judgment** |
| Recruitment strategy | Children aged 1-59 months with respiratory infection (severe cough or tachypnea with axillary temperature above 38°C) who were hospitalized in pediatric departments of two hospitals were enrolled from January 2015 to June 2016. Children <one month or >five years and children suffering from heart disease or chronic respiratory infection were excluded. The study selection process was independent of the exposure or outcome status. However, there is no documentation regarding who selected the study participants; if researcher led there might be a risk of selection bias. | Probably Low |
| Exposure assessment | Data on potential risk factors were gathered by confidential interview based on a questionnaire. Smoking is a sensitive issue in some communities and there might be reporting bias. | Probably High |
| Outcome assessment | Nasopharyngeal swabbing was processed using a molecular method. SP was determined by a multiplex real-time PCR with the FTD Respiratory pathogens 21 plus. the study did not report the outcome based on standard diagnosis criteria (international classification of disease or primary care). | Probably Low |
| Confounding bias | The study adjusted for sociodemographic factors (age (months), sex, paternal education, housing quality and crowding, attendance at health center), medical factors. However, the author did not consider other important potential confounding variables such as indoor and outdoor polluting fuels, vaccination status, nutrition, breastfeeding, or birthweight. | Probably high |
| Incomplete outcome data | The study had complete outcome data. | Low |
| Selective reporting | All the study’s specified outcomes were adequately reported. | Low |
| Conflict of interest | The authors declared no competing interests. While this study had a funding source, there is no evidence of financial conflict of interest. However, it should be noted that the absence of a declaration of bias due to financial interest does not rule out the potential for such bias. | Probably Low |
| Other sources of bias | The causeal association may have been underestimated due to the cross-sectional nature of the data. Additionally, the small sample size (n=637) and the institutionalized nature of the study might compromise its true representativeness of the condition within the general population. | Probably Low |

| **#4: Egondi et al., 2018** | | |
| --- | --- | --- |
| **Design** | **Semi-Ecological** | |
| **Participant** | Birth to 59 months | |
| **Exposure** | Exposure to PM2.5 | |
| **Outcome** | Child mortality | |
| **Risk assessment** | **Supportive response** | **Author’s judgment** |
| Recruitment strategy | Child morbidity data for children <five years of age were obtained from nested studies within the NUHDSS, including the Maternal and Child Health (MCH) study and the INDEPTH Vaccination Project (IVP). The MCH study recruited cohorts of mother–child pairs and followed them up every 4 months. The study included a mother–child pair if the mother resided in the slum when pregnant and if the child was ≤6 months old at the time of recruitment. The authors study selection process was independent of exposure or outcome status and there was no evidence of bias in the selection of participants. | Low |
| Exposure assessment | The study conducted a real-time measurement of (PM_2.5_) using DustTrak II 8532 hand-held samplers at a height of about 1.5 m above ground level (where?) with a resolution of 1-min logging. Sampling took place from early morning to evenings according to a fixed route of measurement within areas, including fixed geographical checkpoints (how often)?. The estimated PM2.5 concentrations were averaged at the enumeration area (EA) level and then the EA average PM2.5 concentrations were assigned to each household/everyone within each EA. the exposure level was categorized to either < or ≥ 25 *µ*g/m^3^, however the measurements were done for less than 24 hrs and so do not represent the diurnal range in pollutant concentrations. Though the study was conducted on multiple day, this categorization may have over or under estimated the concentrations given they did not record over 24 hrs. The assignment to Enumeration Areas (EA) in the study did not account for individuals who may have moved out of the EA during the study period. This oversight may lead to a failure in accurately representing the true average population exposure. | Probably Low |
| Outcome assessment | The study utilized child mortality data derived from nested studies within the Nairobi Urban Health and Demographic Surveillance System (NUHDSS). NUHDSS registers demographic events such as births, deaths, and migration every four months, providing data on individual participants. The Verbal Autopsy (VA) process, integrated into NUHDSS, determines the probable cause of death (COD) using a standardized questionnaire. VA interviews are conducted promptly after a death is reported to minimize recall or reporting bias. Field supervisors visit the affected households to console the bereaved family. The final cause of death is determined through the consensus of two out of three physicians who independently review the VA questionnaires and assign a single COD based on the ICD-10. | Low |
| Confounding bias | The study adjusted for household wealth index, sex, and age of the child. However, other important potentially confounding variables such as meteorological factors, season, smoking in the households, population density, activity, time trends, region, occupation, and other socioeconomic status were not adjusted for. | Probably high |
| Incomplete outcome data | The study had complete outcome data. | Low |
| Selective reporting | All the study’s specified outcomes were adequately reported. | Low |
| Conflict of interest | The author reported no funding received for this study and further declared no conflict of interest | Low |
| Other biases | All-cause mortality might include mortality does not associate with PM_2.5_ | Probably Low |

| **#5: Ezeh et al., 2014** | | |
| --- | --- | --- |
| **Design** | **Cross-sectional** | |
| **Participant** | Under five years | |
| **Exposure** | Exposure to solid fuel during cooking | |
| **Outcome** | Neonatal mortality (death between birth and 28 days of age), post-neonatal mortality (death between 1 and 11 months of age) and child mortality (death between 12 and 59 months of age) | |
| **Risk assessment** | **Supportive response** | **Author’s judgment** |
| Recruitment strategy | This study is a secondary analysis of data from NDHS conducted in Nigeria. The samples are based on a two-stage cluster design where, firstly, Enumeration Areas (EA) are drawn from census files and, in the second stage, in each EA selected, a sample of households is drawn from an updated list of households. The study selection process was unrelated to exposure or outcome status and no means to conduct bias in the selection of participants. | Low |
| Exposure assessment | The study used data from DHS and, respondents were asked “What type of fuel does your household mainly use for cooking, which responses categorized into two groups based on NDHS definitions, solid (coal/lignite, charcoal, wood, straw/shrubs/grass, agricultural crop, animal dung) and non-solid (electricity, liquefied petroleum gas (LPG), natural gas, biogas, kerosene) fuels. The study is susceptible to recall bias due to the nature of the data acquisition measurement employed, and it would be better to complement this method with direct observation of the fuels. Additionally, there is a lack of information on how to categorize for mixed users. | Probably High |
| Outcome assessment | Neonatal mortality (death between birth and 28 days of age), post-neonatal mortality (death between 1 and 11 months of age) and child mortality (death between 12 and 59 months of age). Several The data was obtained through self-report from respondents. Despite being a major event there might be potential for recall bias or inaccuracy in reporting. | Probably Low |
| Confounding bias | The study controlled for potential confounders including residence, household wealth index, mothers (age, education and working status, perceived baby size), sex, breastfeeding status and location of kitchen. The study did not made adjustment for other important confounding variables such as, birth-interval, birthweight, vaccination status, malnutrition, overcrowding condition, safety of drinking water, region, other existing disease condition. | Probably high |
| Incomplete outcome data | Important variables such as ventilation facility, duration of cooking, and frequency of cooking were not used in this analysis because they were not collected in the 2013 NDHS | Probably Low |
| Selective reporting | All the study’s specified outcomes were adequately reported. | Low |
| Conflict of interest | The author reported no funding received for this study and further declared no conflict of interest | Low |
| Other sources of bias | Causal effects could not be established because the analyses were based on a retrospective cross-sectional study. The all-cause mortality may include mortality outcomes that were not associated to maternal smoking and solid fuel use | Probably Low |

| **#6: Fakunle et al., 2014** | | |
| --- | --- | --- |
| **Design** | **Cross-sectional** | |
| **Participants** | Children under 5 years. | |
| **Exposure** | Exposure to firewood for cooking, lantern smoke, smoking in the house, carrying the child while cooking | |
| **Outcome** | ARI | |
| **Risk assessment** | **supportive response** | **Author’s judgment** |
| Recruitment strategy | Cases were recruited with the World Health Organization’s definition of ARI and Controls were selected consecutively from children under 5 who were the same age and gender and whose mothers had the same level of education. Children with chronic illness, congenital heart disease, asthma, recurrent pneumonia, measles or a history of measles in the last month were excluded. The study selection process was independent of the exposure or outcome status. However, there is no documentation regarding who selected the study participants, if the researcher conducted the process there might be a risk of selection bias. It is possible that controls shared some risk factors with cases which were hidden. | Probably Low |
| Exposure assessment | The data was collected from a mother using an interviewer. The study is susceptible to recall bias due to the nature of the data acquisition measurement employed, and it would be better to complement this method with direct observation of the fuels. Additionally, there is a lack of information on how to categorize for mixed users. | Probably High |
| Outcome assessment | The study employed the World Health Organization's definition of ARI. However, there is a lack of documentation regarding who was responsible for measuring the outcome. If researchers conducted the process themselves, there could be a risk of measurement bias due to lack of blinding. | Probably Low |
| Confounding bias | The study controlled for age in months, keeping pet/livestock, use of mosquito coils, family history of ARI, previous ARI. However, the study did not observer other important confounders such as vaccination status, malnutrition, breastfeeding, birth weight and outdoor pollutants | Probably high |
| Incomplete outcome data | The study did not have incomplete outcome data | Low |
| Selective reporting | All the study’s specified outcomes were adequately reported. | Low |
| Conflict of interest | The study did not report its funding source, and there is no information available regarding the presence of any conflicts of interest. | Probably high |
| Other bias | The case-control study might demonstrate a stronger causal relationship; however, it carries a risk of recall bias. | Probably Low |

| **#7: Flanagan et al., 2022** | | |
| --- | --- | --- |
| **Design** | **Cross-sectional** | |
| **Participant** | pregnant women were who first visit to an antenatal care clinic for the current pregnancy | |
| **Exposure** | Exposure to ambient air pollution and indoor air pollution | |
| **Outcome** | Neonatal death | |
| **Risk assessment** | **Supportive response** | **Author’s judgment** |
| Recruitment strategy | Pregnant women were recruited during their first visit to an antenatal care clinic for the current pregnancy after providing informed consent. The study selection process was unrelated to exposure or outcome status and no means to conduct bias in the selection of participants. | Low |
| Exposure assessment | Nitrogen oxides (NOX) and nitrogen dioxide (NO2) were measured at over 40 sites during both wet and dry seasons (for six days each) in Adama, Ethiopia. Land-use regression (LUR) models were developed to assess NOX and NO2 exposure. Data collection involved the use of a Thermo Scientific NO-NO2NOX analyzer (model 42i) at the Ethiopian Meteorological Institute site, although the data reported several gaps. The LUR was also unable to account for some sources of ambient air pollution, including outdoor solid waste burning and traffic intensity. Enumeration Areas (EA) assignments in the study did not consider individuals who might have relocated during the study, potentially skewing population exposure estimates.  Indoor air pollution data, gathered through interviews and categorized into "clean fuel" (electricity, gas, kerosene) and "solid fuel" (wood, charcoal, cow dung), displayed exposure misclassification issues kerosene. The study also lacks confirmation of freedom from recall bias in fuel through observation of fuels. | Probably high |
| Outcome assessment | Outcome data was derived from self-reported questionnaire responses during the participants’ postnatal visit or by phone if an in-person meeting was not possible. Despite being a major event there might be potential for recall bias or underreporting. | Probably Low |
| Confounding bias | Adjusting for confounders identified a priori, including age, education, parity, and HIV status was done. However, pre-pregnancy BMI, nutritional habits and/or folic acid supplementation, parity, low birthweight and pre-eclampsia was not controlled. Self-reported data on lifestyle and behavioral risk factors (smoking status, environmental tobacco smoke exposure, khat usage, and alcohol consumption) may also be prone to response-bias and recall-bias. | Probably high |
| Incomplete outcome data | The study did not obtain geographic coordinates for a significant number (n=261) of study participants. Additionally, 469 women did not complete the study. However, the analysis excludes these participants, conducting the analysis only with those who did complete the study. The missed data may avert the true association between exposure and outcome variable | Probably high |
| Selective reporting | All the study’s specified outcomes were adequately reported. | Low |
| Conflict of interest | The study was funded, and the author declared has no known conflict of interest for the study. | Probably Low |
| Other bias | The small sample size in the study might compromise its true representativeness of the condition within the general population. | Probably Low |

| **#8: Francisco et al., 1993** | | |
| --- | --- | --- |
| **Design** | **Case-Control** | |
| **Participant** | Children aged < 2 years | |
| **Exposure** | Exposure to parental smoking, mother carried child while cooking and indoor air pollution | |
| **Outcome** | ALRI death | |
| **Risk assessment** | **Supportive response** | **Author’s judgment** |
| Recruitment strategy | A mortality surveillance system, facilitated by a network of village reporters, was established. Verbal autopsies were conducted to gather information about the causes of death from mothers or guardians. Subsequently, the cause of death was determined independently by three physicians. A cause of death was accepted when at least two physicians agreed on the diagnosis, which occurred for 92% of the deaths in the present study. The study selection process was independent of the exposure or outcome status. Additionally, the study did not report the diagnostic methods employed by the physicians, whether they relied on laboratory tests or standard diagnostic criteria such as the International Classification of Diseases or Primary Care. | Probably high |
| Exposure assessment | A detailed questionnaire which covered potential risk factors for death from ALRI was administered to the families of cases and controls as soon as possible after the death of a case. The study is susceptible to recall bias due to the nature of the data acquisition measurement employed, and it would be better to complement this method with direct observation of the fuels. Smoking is sensitive issue to some communities and may be underreported through interviewing. Additionally, there is a lack of information on how to categorize for mixed users. | Probably High |
| Outcome assessment | Deaths in children aged < 5 years are recorded and cause of death was confirmed by three physicians and was accepted when at least two physicians agree on the diagnosis. The study did not report the diagnostic methods employed by the physicians, whether they relied on laboratory tests or standard diagnostic criteria such as the International Classification of Diseases or Primary Care. | Probably Low |
| Confounding bias | The study controlled for seriocomic score, maternal education, indicators of crowding and health factors (vaccination, malnutrition, ANC visit, and treatment). The study did not consider other important confounding variables such as birth-interval, birthweight, breastfeeding status, safety of drinking water, place of residence | Probably Low |
| Incomplete outcome data | The study had not missing data | Low |
| Selective reporting | All of the study’s specified outcomes were adequately reported. | Low |
| Conflict of interest | The study did not report its funding source, and there is no information available regarding the presence of any conflicts of interest. | Probably high |
| Other bias | The case-control study might demonstrate a stronger causal relationship; however, it carries a risk of recall bias. Additionally, the institutionalized nature of the study might compromise its true representativeness of the condition within the general population. | Probably Low |

| **#9: Heathfield et al., 2020** | | |
| --- | --- | --- |
| **Design** | **Cross-sectional** | |
| **Participant** | Infants | |
| **Exposure** | Exposure to passive smoking from mothers or other family members in house | |
| **Outcome** | Infant death | |
| **Risk assessment** | **Supportive response** | **Author’s judgment** |
| Recruitment strategy | The study included those of individuals younger than 1 year at the time of death who thought to be due to unnatural causes as defined in the National Health Act No. 61 of 2003. The study selection process was independent of the exposure or outcome status. However, there is no documentation regarding who selected the study participants, if the researcher conducted the process there might be a risk of selection bias. | Probably Low |
| Exposure assessment | For each case, information pertaining to demographics, environment, death scene, circumstances surrounding death, risk factors, forensic investigation and cause of death was collected from the medico-legal case folders/ archives. The study lacks assurance against reporting bias, as smoking status may be underreported by respondents. | Probably High |
| Outcome assessment | The study collected outcome information from medico-legal case folders in the archives. Using data from a vital registration system would enhance accuracy compared to self-reports from mothers. However, the study does not provide information on the functionality of the registration system. | Probably Low |
| Confounding bias | The study is pure descriptive study and didn’t consider confounding adjusting. | High |
| Incomplete outcome data | The study reported missing data, attributed either to misplaced documentation or unanswered questions during the postmortem investigation process. This limitation impedes the ability to observe associations between SUDI cases and demographic factors. | Probably high |
| Selective reporting | All the study’s specified outcomes were adequately reported. | Low |
| Conflict of interest | The study was not funded, and the author declared has no known conflict of interest for the study. | Probably Low |
| Other bias | No other potential source of bias is suspected | Low |

| **#10: Imo et al., 2023** | | |
| --- | --- | --- |
| **Design** | **Cross-sectional** | |
| **Participant** | Under-five children | |
| **Exposure** | Exposure to solid fuel for cooking | |
| **Outcome** | Under-five mortality | |
| **Risk assessment** | **Supportive response** | **Author’s judgment** |
| Recruitment strategy | This study is a secondary analysis of data from NDHS conducted in Nigeria. The samples are based on a two-stage cluster design where, firstly, Enumeration Areas (EA) are drawn from census files and, in the second stage, in each EA selected, a sample of households is drawn from an updated list of households. The study selection process was independent to exposure or outcome status and no means to conduct bias in the selection of participants. | Low |
| Exposure assessment | Exposure data were collected through interviews utilizing a structured questionnaire. Cooking fuel was subsequently categorized into solid fuels (coal/lignite, charcoal, wood, straw/shrubs/grass, agricultural crops, and animal dung) and non-solid fuels (electricity, gas, and kerosene). The use of solid fuels for cooking was employed as a proxy measure. However, the study lacks assurance against recall bias, as direct observation of the fuels was not conducted. Furthermore, the categorization of kerosene with other clean fuels results in misclassification of exposure status. Additionally, there is insufficient information provided on how to categorize mixed users. | Probably High |
| Outcome assessment | The study measured the outcome variable as the duration of survival since birth, recorded in months. Survival time was determined by the age at death, while children who were still alive at the survey date were censored at their current age. Due to the self-reporting nature of the data collected from women, there is a possibility of reporting bias. | Probably Low |
| Confounding bias | The study controlled maternal age, paternal education, occupation, region, residence and neighborhood poverty. However, the study did not made adjustment for other important confounding variables such as birth-interval, birthweight, vaccination status, breastfeeding status, malnutrition, overcrowding condition, safety of drinking water, paternal smoking in the household and other existing disease condition. | Probably high |
| Incomplete outcome data | Missing data were excluded from the analysis by considering only women who reported using cooking fuels in kitchens situated within their households. | Probably Low |
| Selective reporting | All the study’s specified outcomes were adequately reported. | Low |
| Conflict of interest | The study had no specific funding source, and the authors declared no conflicts of Interest. | Low |
| Other sources of bias | Cause effect association may have been underestimated due to the cross-sectional nature of the data. The all-cause mortality may include mortality outcomes that were not associated to solid fuel use. | Probably Low |

| **#11 Johnson et al., 1992** | | |
| --- | --- | --- |
| **Design** | **Case-control** | |
| **Participant** | children aged between 2 weeks and 59 months | |
| **Exposure** | Exposure to household pollutants | |
| **Outcome** | Outcome of ARI hospitalization | |
| **Risk assessment** | **Supportive response** | **Author’s judgment** |
| Recruitment strategy | The study included children aged 2 weeks to 59 months diagnosed with acute lower respiratory infections (ALRI), such as croup, bronchiolitis, pneumonia, and empyema thoracis. To ensure thorough evaluation, only four cases were admitted per week. Age-matched controls were selected within 4 days of subject recruitment, consisting of healthy children attending the same hospital for immunizations, those hospitalized for elective surgery, and those receiving routine follow-up for non-respiratory ailments. The selection process was consistent for both groups and unrelated to exposure or outcome status. However, there is no documentation on who conducted the participant selection, posing a risk of selection bias if researchers were involved. Additionally, the diagnosis of lower respiratory disease using the Glasgow, Manchester, Newcastle, Birmingham criteria may not accurately reflect the true prevalence of specific illnesses. | Probably Low |
| Exposure assessment | The study reported collecting information on domestic variables, such as parents' and close neighbors' smoking habits, kitchen fuel type, cooking area location, parental occupation and income, and household size. However, the methods used for data collection, whether through face-to-face interviews, self-administered questionnaires, or direct observation, were not explained. | Probably High |
| Outcome assessment | The cause death ARI was diagnosed of using the Glasgow, Manchester, Newcastle, Birmingham criteria and this may not accurately reflect the true prevalence of specific cause. | Probably Low |
| Confounding bias | Binary association analysis was conducted using the chi-square test, but no multivariable analysis was performed to address potential confounding variables. | High |
| Incomplete outcome data | The study did not have incomplete outcome data | Low |
| Selective reporting | All the study’s specified outcomes were adequately reported. | Low |
| Conflict of interest | The study had funding sources but no evidence for financial conflict of interest. | Low |
| Other sources of bias | The institutionalized nature of the study may compromise its true representation of the condition within the general population. | Probably Low |

| **#12 Johnson et al., 2008** | | |
| --- | --- | --- |
| **Design** | **Cross-sectional** | |
| **Participant** | Hospitalized children aged 2 weeks to 59 months | |
| **Exposure** | Exposure to kitchen smoke | |
| **Outcome** | CAP-associated mortality | |
| **Risk assessment** | **Supportive response** | **Author’s judgment** |
| Recruitment strategy | Children aged 2 weeks to 5 years with symptom complex of a lower respiratory infection that satisfied the ALRI syndrome definition of Denny and Clyde were recruited prospectively on alternate weekdays. Otherwise-eligible subjects with clinical and/or radiographic features of pulmonary tuberculosis were excluded. The selection process was consistent for both groups and unrelated to exposure or outcome status. However, there is no documentation on who conducted the participant selection, posing a risk of selection bias if researchers were involved. | Probably Low |
| Exposure assessment | The authors reported collecting information on recognized risk factors for respiratory illnesses, including exposure to domestic cigarette and kitchen smoke, immunization status, feeding practices, and parental socioeconomic circumstances. This data was recorded in a precoded questionnaire. However, the study lacks assurance against recall bias, as direct observation of fuels was not conducted. Moreover, due to the sensitive nature of smoking in some communities, it may be underreported during interviews. Furthermore, there is insufficient information provided on how to categorize kitchen smoke, including the type of smoke and the type of kitchen used. | Probably High |
| Outcome assessment | The study was a follow-up investigation, with outcomes focused on both mortality and survival rates, as well as the length of hospital admission among survivors. The diagnosis of the cause of death relied on radiographic and clinical findings. | Low |
| Confounding bias | The relationship between agents and outcome variables was assessed using the Chi-squared test, with or without Yates' or the Mantel-Haenszel correction, or the Fisher's exact test for categorical variables. Significant differences in continuous variables were examined using either Student's t-test or analysis of variance (ANOVA). However, the study did not conduct multivariable analysis to control for the effects of confounding variables. | Probably high |
| Incomplete outcome data | The study did not have incomplete outcome data | Low |
| Selective reporting | All the study’s specified outcomes were adequately reported. | Low |
| Conflict of interest | The study appears to have had a funding source, and no competing interests were declared. However, there is no evidence of financial conflict of interest. | Probably low |
| Other sources of bias | The inherent limitations in the study design and analysis method preclude drawing definitive conclusions regarding the association of wood smoke with CAP fatality. | Probably high |

| **#13 Kiconco et al., 2021** | | |
| --- | --- | --- |
| **Design** | **Cross-sectional** | |
| **Participant** | Under-five children | |
| **Exposure** | Place of cooking and exposure to parental smoking | |
| **Outcome** | Pneumonia | |
| **Risk assessment** | **Supportive response** | **Author’s judgment** |
| Recruitment strategy | the study all children aged 2-59 months who attended to KIU-TH with acute respiratory symptoms during the study period were consecutively recruited for the study. The study excluded children with obvious clinical features of acute aspiration (near drowning and acute foreign body inhalation) since there was a known cause. The selection process was consistent for both groups and unrelated to exposure or outcome status. However, there is no documentation on who conducted the participant selection, posing a risk of selection bias if researchers were involved. | Probably Low |
| Exposure assessment | A structured questionnaire was employed to gather exposure variables. However, the study lacks assurance against recall bias since direct observation of fuels was not conducted. Additionally, the sensitive nature of smoking in certain communities may lead to underreporting during interviews. Furthermore, there is insufficient information regarding the location of cooking, especially for mixed users or those cooking in separate buildings versus in-house users. | Probably High |
| Outcome assessment | The study utilizes the WHO definition of pneumonia, which includes the presence of cough and/or difficulty in breathing, along with fast breathing and/or chest indrawing. This definition was further modified to include positive chest X-ray findings of pneumonia. | Low |
| Confounding bias | The study controlled for age, sex, residence, parental/caretaker education, marital status, religion, tribe, immunization, breastfeeding, and nutritional status. However, the study did not control other important confounding such as birthweight, outdoor pollutants, crowding and existing health conditions like HIV and TB. | Probably high |
| Incomplete outcome data | The study did not have incomplete outcome data | Low |
| Selective reporting | All the study’s specified outcomes were adequately reported. | Low |
| Conflict of interest | The authors did not report any funding sources. However, they declared no conflicts of interest, and there is no evidence of financial conflict of interest. | Probably Low |
| Other bias | The inherent limitations in the study design preclude drawing definitive conclusions regarding the association exposure and outcome variable. The institutionalized nature of the study may compromise its true representation of the condition within the general population. | Probably Low |

| **#14 Kleimola et al., 2015** | | |
| --- | --- | --- |
| **Design** | **Cross-sectional** | |
| **Participant** | Neonatal (0–28 days) and child (from 29 days to 59 months) | |
| **Exposure** | Exposure to polluting fuels | |
| **Outcome** | Neonatal and child mortality | |
| **Risk assessment** | **Supportive response** | **Author’s judgment** |
| Recruitment strategy | The study used DHS data. Households are usually selected through stratified two-stage cluster sampling with census enumeration areas as primary sampling units. Men aged 15–59 years and women aged 15–49 years in sampled households are then interviewed by trained field workers. The study selection process was independent to exposure or outcome status and no means to conduct bias in the selection of participants | Low |
| Exposure assessment | The Household Questionnaire is used to collect information on demographic and health information of household residents and visitors as well as household characteristics, including type of cooking fuel used by the household. The exposure of interest was type of fuel the household “mainly use[s] for cooking,” categorized into clean fuels (electricity, liquid petroleum gas, natural gas, and biogas), kerosene, and solid fuels (coal, charcoal, and biomass such as wood, crop waste, and dung). The study is susceptible to recall bias due to the nature of the data acquisition measurement employed, and it would be better to complement this method with direct observation of the fuels. Additionally, there is a lack of information on how to categorize for mixed users. | Probably High |
| Outcome assessment | The outcomes of birth history, including information on the age at death of all liveborn children, were obtained through maternal interviews. The data was self-reported by respondents. Despite being a significant event, there is potential for recall bias or inaccuracies in reporting. | Probably Low |
| Confounding bias | The study controlled for maternal education, mother's age at birth, maternal smoking, birth order, child's sex, wealth index, urban versus rural residence, and country of residence. However, adjustments were not made for other significant confounding variables, such as birthweight, vaccination status, breastfeeding status, malnutrition, overcrowding, water safety, paternal smoking, household income, and existing disease conditions. Residual confounding may still exist from factors influencing mortality that were not accounted for in the analysis. | Probably High |
| Incomplete outcome data | Children with missing information on household cooking fuel use (n = 586), those whose households responded "other" (n = 415) or "no food cooked in household" (n = 475) regarding the type of cooking fuel used, children with missing information on any covariate (n = 118), and children with inconsistent or unknown ages at death (n = 288) were excluded from the analysis. Omission of underreported child deaths on the DHS is most common for deaths occurring in early infancy, rendering the current study sample susceptible to omitted deaths, particularly in the neonatal population. However, the study excluded this missing data from the analysis, and the large sample size may mitigate the impact of the omitted data. | Probably Low |
| Selective reporting | All the study’s specified outcomes were adequately reported. | Probably Low |
| Conflict of interest | The authors did not report any funding sources. However, they declared no conflicts of interest, and there is no evidence of financial conflict of interest. | Probably Low |
| Other bias | The inherent limitations in the study design preclude drawing definitive conclusions regarding the association exposure and outcome variable. The all-cause mortality may include mortality outcomes that were not associated to solid fuel use. | Probably Low |

| **#15: Latona et al., 2017** | | |
| --- | --- | --- |
| **Design** | **Cross-sectional** | |
| **Participant** | Children under 5 years | |
| **Exposure** | Exposure to biomass cooking fuels | |
| **Outcome** | Under-five mortality | |
| **Risk assessment** | **Supportive response** | **Author’s judgment** |
| Recruitment strategy | This study uses data from the Nigeria Demographic and Health Survey (NDHS) 2008. Sample was selected using a stratified two-stage cluster design consisting of 888 clusters, 286 in the urban and 602 in the rural areas. Also, an average of 41 households was selected in each cluster, by equal probability systematic sampling. All women aged 15-49 years who were either permanent residents of the households in the 2008 NDHS sample or visitors present in the households on the night before the survey were eligible to be interviewed. The study selection process was independent to exposure or outcome status and no means to conduct bias in the selection of participants. | Low |
| Exposure assessment | The data were obtained from women aged 15-49 years who were interviewed about household characteristics using a structured questionnaire. However, the study lacks assurance against recall bias since direct observation of fuels was not conducted. Furthermore, there is insufficient information provided on how to categorize biomass and non-biomass fuels, including those used by mixed users. | Probably High |
| Outcome assessment | The outcomes of birth history, including information on the age at death of all liveborn children, were obtained through maternal interviews. The data was self-reported by respondents. Despite being a significant event, there is potential for recall bias or inaccuracies in reporting. | Probably Low |
| Confounding bias | The authors conducted Cox proportional hazard regression and controlled for region of residence, religion, wealth index, household size, availability of toilet facility, condition of the floor, size of baby at birth, preceding birth interval, and postnatal visit within 2 months. However, the study did not adjust for other important confounding variables such as maternal age and education status, vaccination status, breastfeeding status, malnutrition, overcrowding, safety of drinking water, and other existing disease conditions. | Probably high |
| Incomplete outcome data | One of the main challenges in collecting retrospective information on births and deaths is the underreporting of births and deaths for children who were not living at the time of the survey. Omission of underreported child deaths on the DHS is most common. However, the study excluded this missing data from the analysis, and the large sample size may help mitigate the impact of the omitted data. | Probably Low |
| Selective reporting | All the study’s specified outcomes were adequately reported. | Low |
| Conflict of interest | The study did not provide information about funding source and conflict of interest. | Probably High |
| Other sources of bias | The inherent limitations in the study design preclude drawing definitive conclusions regarding the association exposure and outcome variable. The all-cause mortality may include mortality outcomes that were not associated to biomass fuel use. | Probably Low |

| **#16: Nantanda et al., 2013** | | |
| --- | --- | --- |
| **Design** | **Cross-sectional** | |
| **Participant** | Children aged 2 to 59 months | |
| **Exposure** | Exposure to Tobacco smoking and gas cooking | |
| **Outcome** | Asthma and Bronchitis | |
| **Risk assessment** | **Supportive response** | **Author’s judgment** |
| Recruitment strategy | The study enrolled children aged 2 to 59 months who presented at the pediatric emergency unit of Mulago Hospital with symptoms of cough and/or difficulty in breathing, in addition to fast breathing. Caretakers provided informed written consent for their participation. Children with heart conditions or cardiac failure secondary to severe anemia, as determined by the caretaker's history, physical examination findings, and medical records, were excluded. Only children with acute asthma symptoms were recruited. The standardized WHO definition was used to enroll study participants. The selection process was independent of exposure or outcome status. However, there is no documentation regarding who selected the study participants; if the researcher conducted the process, there might be a risk of selection bias. | Probably Low |
| Exposure assessment | The data were collected using a pretested questionnaire administered by nurses. However, the study lacks assurance against recall bias as direct observation of fuels was not conducted. Collecting information about tobacco smoking, which is sensitive for some individuals, may lead to underreporting or inaccuracies in reporting. Additionally, there is insufficient information on how to categorize mixed users. | Probably High |
| Outcome assessment | The modified version of the GINA (Global Initiative for Asthma) guidelines was used for asthma diagnosis. However, there are still no universally accepted gold standard diagnostic criteria available for childhood asthma diagnosis. For bronchiolitis, the South African guidelines for diagnosis, management, and prevention of acute viral bronchiolitis were utilized. | Probably Low |
| Confounding bias | The study controlled for sex, history of allergy, maternal asthma, prematurity, exclusive breastfeeding for last three months, education level of caretaker. However, the study did not consider other important confounding variables such as vaccination status, malnutrition, crowding, birthweight, outdoor pollutant, existing conditions like HIV and TB. | Probably high |
| Incomplete outcome data | The study did not have incomplete outcome data. | Low |
| Selective reporting | All the study’s specified outcomes were adequately reported. | Low |
| Conflict of interest | The study was funded, and the authors declared no conflict of interest. | Probably Low |
| Other bias | The inherent limitations in the study design preclude drawing definitive conclusions regarding the association exposure and outcome variable. Additionally, the institutionalized nature of the study might compromise its true representativeness of the condition within the general population. | Probably Low |

| **#17: Ngocho et al., 2019** | | |
| --- | --- | --- |
| **Design** | **Case control** | |
| **Participant** | Children aged 2–59 months, | |
| **Exposure** | Exposure to unclean cooking fuel and parental smoking | |
| **Outcome** | Pneumonia | |
| **Risk assessment** | **Supportive response** | **Author’s judgment** |
| Recruitment strategy | The study recruited hospitalized children aged 2–59 months who met the WHO case definition for pneumonia. Trained research nurses conducted the recruitment process, obtaining consent from cases with X-ray-confirmed pneumonia to participate in the study. Controls matching the characteristics (sex and age; +1 month) of the cases were identified and recruited from the community. The selection process was random and did not bias exposure or outcome status. However, the selection of cases from hospitals and controls from the community may introduce inconsistencies across groups. It is possible that controls shared some risk factors with cases that were not accounted for. | Probably Low |
| Exposure assessment | The data were collected through interviews, and families using gas or electricity for cooking were categorized as using clean cooking fuel, while those using biomass, firewood, charcoal, and kerosene were considered to use unclean fuel. However, the study lacks assurance against recall bias as direct observation of fuels was not conducted. The parents of children with CAP may have underreported their smoking habits because they knew the health consequences of smoking. Additionally, there is insufficient information on how to categorize mixed users. | Probably High |
| Outcome assessment | The WHO case definition of pneumonia was utilized, with X-ray confirmation conducted to support the diagnosis. | Low |
| Confounding bias | The study controlled for several important variables, including maternal characteristics (age, education, marital status, income, medical insurance), crowding, age, sex, birthweight, vaccination status, breastfeeding status, malnutrition, overcrowding condition, and safety of drinking water. However, adjustments were not made for inter-birth interval and other existing disease conditions. | Probably Low |
| Incomplete outcome data | The study has a 100% reported rate, but for some variables such as smoking habits. | Probably Low |
| Selective reporting | All the study’s specified outcomes were adequately reported. | Low |
| Conflict of interest | The study was funded, and the authors declared no conflict of interest. More the study provide information about the funded body had no any role in the study process. | Probably Low |
| Other bias | The institutionalized nature of the study might compromise its true representativeness of the condition within the general population. | Probably Low |

| **#18: Owili et al., 2017** | | |
| --- | --- | --- |
| **Design** | **Cross-sectional** | |
| **Participant** | 0–59 months | |
| **Exposure** | Exposure to cooking fuel used in the household | |
| **Outcome** | Under-five mortality | |
| **Risk assessment** | **Supportive response** | **Author’s judgment** |
| Recruitment strategy | The study used DHS data. Households are usually selected through stratified two-stage cluster sampling with census enumeration areas as primary sampling units. Men aged 15–59 years and women aged 15–49 years in sampled households are then interviewed by trained field workers. The authors study selection process was independent to exposure or outcome status and no means to conduct bias in the selection of participants. | Low |
| Exposure assessment | The data were collected from women aged 15-49 years through interviews regarding household characteristics using a structured questionnaire. However, the study lacks assurance against recall bias since direct observation of fuels was not conducted. The study classified fuels into four main groups: 'clean' fuel (electricity, natural gas, biogas, or liquefied petroleum gas) as the reference group; biomass cooking fuels divided into 'charcoal' (mainly used indoors) and other 'biomass' fuels (mainly used outdoors for cooking, such as wood, straw/shrubs/grass, agricultural crops, or animal dung); and 'other' pollutant cooking fuel (coal, lignite, or paraffin/kerosene). Additionally, there is insufficient information provided on how to categorize those used by mixed users. | Probably High |
| Outcome assessment | The study defines our outcome indicator as all-cause mortality of children under five, with time-to-event being age in months (0 – 59), represented as 1 for yes and 0 otherwise. The under-five mortality question inquired whether the child was alive or deceased at the time of the interview; if the child was deceased, their age at death was recorded. Several DHS studies have shown evidence of downward bias in reporting child deaths. | Probably Low |
| Confounding bias | The study adjusted for kitchen location, country, residence, child's sex, breastfeeding status, number of under-5 children, mother's age, family size, wealth index, mother's education, mother's occupation, and father's occupation. However, it could not account for unmeasured potential confounders such as specific details of tobacco smoking (e.g., number of cigarettes smoked in the household) and infectious diseases affecting both living and deceased children. Additionally, other environmental, social, genetic, and health-related factors such as unsafe water, incomplete vaccination, short inter-birth intervals, and low birthweight could have influenced the outcomes. | Probably high |
| Incomplete outcome data | The DHS survey data were collected for general purposes and may therefore be limited in the information contained therein. However, the analysis was restricted to complete data, and the large sample size may help mitigate the impact of those missing data on overall analysis result. | Probably Low |
| Selective reporting | All the study’s specified outcomes were adequately reported. | Low |
| Conflict of interest | The study was funded but the funder had no role in the study design and overall analytical steps and the authors declared no conflict of interest. | Probably Low |
| Other bias | This study was based on cross-sectional data collected retrospectively, thus making it challenging to ascertain a true measure of causality. | Probably Low |

| **#19: PrayGod., 2016** | | |
| --- | --- | --- |
| **Design** | **Case-control** | |
| **Participant** | Children aged 2–59 months | |
| **Exposure** | Exposure to indoor cooking fuel and parental smoking | |
| **Outcome** | Severe pneumonia | |
| **Risk assessment** | **Supportive response** | **Author’s judgment** |
| Recruitment strategy | Children diagnosed with either severe pneumonia or very severe pneumonia based on World Health Organization definitions at admission, who had not yet received treatment for these conditions, were defined as cases. Controls included children aged 2 to 59 years attending the outpatient department or admitted into the hospital pediatric ward on the same day as cases, diagnosed with illnesses other than pneumonia, severe pneumonia, or very severe pneumonia. The selection process was independent of exposure or outcome status. Selecting cases and controls from the same hospital may help maintain consistency across groups. However, there is no documentation regarding who selected the study participants, and if the researcher conducted the process, there might be a risk of selection bias. It is possible that controls shared some risk factors with cases that were not accounted for. | Probably Low |
| Exposure assessment | The study also collected data on the source of cooking fuel (electricity, gas, wood charcoal, or firewood) and whether food preparation occurred indoors or outdoors to determine the role of indoor air pollution as a risk factor for severe pneumonia. Additionally, the authors inquired whether either parent was a regular smoker by interviewing the guardian or parent using structured questionnaires. However, the study lacks assurance against recall bias since direct observation of fuels was not conducted. Parents of children may have underreported their smoking habits due to awareness of the health consequences of smoking. Furthermore, there is insufficient information on how to categorize mixed users. | Probably High |
| Outcome assessment | The study employed World Health Organization case definitions of severe pneumonia, identifying it by fast breathing (>50 breaths per minute for those aged 2 to 11 months and >40 breaths for those aged 12 to 59 months). However, laboratory confirmation of the case definition was not conducted, and there remains a risk of bias if researchers were involved in the process without proper blinding procedures. | Probably Low |
| Confounding bias | The study adjusted for age in months, sex, measle vaccination, vitamin-A supplementation and, Enterobacter spp. Other important potential confounding variables including maternal age and education status, birth-interval, birthweight, breastfeeding status, malnutrition, overcrowding condition, safety of drinking water, place of residence, household income should also be considered. Additionally, diarrhoea may increase the risk of pneumonia. Therefore, there is a possibility that inclusion of these patients reduced the strength of associations of the reported risk factors. | Probably High |
| Incomplete outcome data | The study did not have incomplete outcome data. | Low |
| Selective reporting | All the study’s specified outcomes were adequately reported. | Low |
| Conflict of interest | The study was funded but the funder had no role in the study design and overall analytical steps. and the authors declared no conflict of interest. | Probably Low |
| Other bias | Follow-up rates and participant retention throughout the study period may vary, leading to attrition bias. If those lost to follow-up have different health outcomes or exposure histories than those who remain, this could skew results. | Probably Low |

| **#20: Roux et al., 2015** | | |
| --- | --- | --- |
| **Design** | **Cohort** | |
| **Participant** | Mother-infant pairs | |
| **Exposure** | Exposure to maternal smoking | |
| **Outcome** | Pneumonia | |
| **Risk assessment** | **Supportive response** | **Author’s judgment** |
| Recruitment strategy | The recruitment strategy, focusing on pregnant women between 20 and 28 weeks of gestation at antenatal clinics, is practical for ensuring consistent follow-up throughout pregnancy and childbirth. However, limiting participation to women over 18 excludes younger mothers who may also be at risk. The exclusion of those intending to relocate within a year ensures continuity in data collection, but it could reduce the diversity of the sample by excluding transient populations. | Probably Low |
| Exposure assessment | Maternal health questionnaires and antenatal data were collected, and obstetric care was centralized at Paarl Hospital, where all births occurred. Infant follow-up was conducted at 6, 10, and 14 weeks, and at 6, 9, and 12 months. Environmental exposures, pneumonia risk factors, anthropometry, and respiratory symptoms were assessed during scheduled visits. Missed visits were rescheduled using a mobile phone system or by community-based fieldworkers. Assessing air pollution via questionnaires relies heavily on self-reported data, which may be subject to recall bias and inaccuracies. Questionnaires are useful for gathering information on potential indoor pollution sources (e.g., cooking practices, smoking), but they cannot accurately measure exposure levels. | Probably High |
| Outcome assessment | The pneumonia outcome assessment employs active case finding with regular maternal interviews, allowing for comprehensive monitoring of respiratory symptoms. Diagnoses adhere to revised WHO guidelines, using clinical signs confirmed by trained staff, which enhances reliability. The exclusion of nosocomial and congenital cases focuses the analysis on community-acquired pneumonia. However, reliance on treating doctors for hospitalization decisions may introduce variability. | Probably Low |
| Confounding bias | The assessment of confounding bias in this study is moderately robust, but there are important considerations. Age categorization at pneumonia onset allows for stratification, but potential confounders, such as socio-economic status, environmental factors, and maternal health, are not explicitly mentioned. The reliance on anthropometric measures and clinical assessments, including CRP levels and chest radiographs, adds reliability to pneumonia diagnosis but may not fully account for all confounding factors influencing health outcomes. While statistical analyses are well-structured, including the use of prevalence ratios and robust variance estimation, the absence of specific covariate adjustment could lead to residual confounding. | Probably High |
| Incomplete outcome data | The study did not have incomplete outcome data. | Low |
| Selective reporting | All the study’s specified outcomes were adequately reported. | Low |
| Conflict of interest | The study was funded but the funder had no role in the study design and overall analytical steps. | Probably Low |
| Other bias | Follow-up rates and participant retention throughout the study period may vary, leading to attrition bias. If those lost to follow-up have different health outcomes or exposure histories than those who remain, this could skew results. | Probably Low |

| **#21: Roux et al., 2021** | | |
| --- | --- | --- |
| **Design** | **Cohort** | |
| **Participant** | Mother-infant pairs | |
| **Exposure** | Exposure to maternal smoking | |
| **Outcome** | Pneumonia | |
| **Risk assessment** | **Supportive response** | **Author’s judgment** |
| Recruitment strategy | The recruitment strategy effectively targets a specific population, focusing on pregnant women in a low socio-economic peri-urban community. The use of a well-established birth cohort (DCHS) enhances the study's credibility and provides a robust framework for long-term follow-up. By including only mothers at 20–28 weeks’ gestation, the study ensures that participants are in a relevant stage for assessing maternal and infant health outcomes. Ethical approval from a recognized committee and the requirement for informed consent further strengthen the recruitment process, ensuring ethical standards are met. | Probably Low |
| Exposure assessment | The exposure assessment employed in this study demonstrates several strengths that contribute to its reliability. The use of a personal air sampling pump for measuring PM_10_ offers direct and accurate data on indoor air quality, with a 24-hour sampling period capturing daily variations in exposure levels. Additionally, the methodology for measuring toluene through diffusion tubes is standardized, ensuring consistency in results. Furthermore, the assessment of maternal smoking through urine cotinine levels provides an objective measure of smoking status, allowing for accurate classification of exposure. | Low |
| Outcome assessment | Mothers were advised to report any respiratory symptoms between scheduled visits, such as cough or difficulty breathing. Trained study nurses diagnosed pneumonia and severe pneumonia using WHO clinical definitions. The diagnostic approach is thorough, following WHO guidelines and ensuring accurate pneumonia identification. However, relying on mothers to report symptoms outside of scheduled visits may lead to underreporting. The criteria for severe pneumonia and hospital admission are appropriate, but self-reports could introduce variability in timely diagnoses. Overall, the strategy is solid but could benefit from more proactive monitoring between visits. | Probably Low |
| Confounding bias | The approach to confounding control is well-structured, using mixed-effect Poisson models to adjust for individual-level variation and relevant risk factors. Including weight-for-age Z-scores in separate models helps clarify its role as a mediator rather than a confounder. However, seasonal categorization may oversimplify the effects of varying weather and environmental conditions on air pollution exposure. It’s unclear if all relevant covariates (e.g., socioeconomic status, household air quality, or vaccination status) are fully accounted for. These factors are critical in air pollution studies and could impact pneumonia risk. | Probably High |
| Incomplete outcome data | The study did not have incomplete outcome data. | Low |
| Selective reporting | All the study’s specified outcomes were adequately reported. | Low |
| Conflict of interest | The study was funded but the funder had no role in the study design and overall analytical steps. and the authors declared no conflict of interest. | Probably Low |
| Other bias | The case-control study might demonstrate a stronger causal relationship; however, it carries a risk of recall bias. The institutionalized nature of the study might compromise its true representativeness of the condition within the general population. | Probably Low |

| **#22: Samuel et al., 2018** | | |
| --- | --- | --- |
| **Design** | **Cross-sectional** | |
| **Participant** | Under-five children | |
| **Exposure** | Exposure to Solid fuel and kitchen inside the house | |
| **Outcome** | Under-five mortality | |
| **Risk assessment** | **Supportive response** | **Author’s judgment** |
| Recruitment strategy | The study used NDHS data. Households are usually selected through stratified two-stage cluster sampling with census enumeration areas as primary sampling units. Men aged 15–59 years and women aged 15–49 years in sampled households are then interviewed by trained field workers. The authors study selection process was independent to exposure or outcome status and no means to conduct bias in the selection of participants. | Low |
| Exposure assessment | Exposure to solid fuel defined those who used wood, dung, charcoal inside the house for cooking. The study is susceptible to recall bias due to the nature of the data acquisition measurement employed, and it would be better to complement this method with direct observation of the fuels. Additionally, there is a lack of information on how to categorize other source fuels and mixed users. | Probably High |
| Outcome assessment | Under-five mortality, death between birth and the fifth birthday of the child and was surveyed. The data was obtained through self-report from respondents. Despite being a major event there might be potential for recall bias or inaccuracy in reporting. | Probably Low |
| Confounding bias | The study adjusted for wealth, residence, education and region. However, the study did not made adjustment for other important confounding variables such as maternal age and education status, birth-interval, birthweight, vaccination status, breastfeeding status, malnutrition, overcrowding condition, safety of drinking water, other existing disease condition. | Probably high |
| Incomplete outcome data | The study utilized a data source with a high response rate (97%), and the analysis was restricted to complete data, including a large sample size. This approach minimizes the significant impact of missing data. | Probably Low |
| Selective reporting | All of the study’s specified outcomes were adequately reported. | Low |
| Conflict of interest | The study had no funding source. | Low |
| Other bias | Cause effect association may have been underestimated due to the cross-sectional nature of the data. The all-cause mortality may include mortality outcomes that were not associated with solid fuel use. | Probably Low |

| **#23: Shifa et al., 2018** | | |
| --- | --- | --- |
| **Design** | **Case-control** | |
| **Participant** | Under-five children | |
| **Exposure** | Exposure to lack separate kitchen in household and non-electric light sources in the household | |
| **Outcome** | Infant and under five mortality | |
| **Risk assessment** | **Supportive response** | **Author’s judgment** |
| Recruitment strategy | The study setting was purposively selected, which may introduce deviation from randomness in the selection process and consequently lead to generalization issues. Subsequently, 383 cases along with their corresponding matched and randomly selected 766 controls that met the inclusion criteria were identified. The selection process was independent of exposure or outcome status. Choosing cases and controls from the same community may aid in maintaining consistency across groups. However, there is no documentation regarding who selected the study participants, and if researchers conducted the process, there might be a risk of selection bias. It is possible that controls shared some risk factors with cases that were not accounted for. | Probably Low |
| Exposure assessment | The exposure variables were collected through interviews using locally translated pretested questionnaires. However, the study lacks assurance against recall bias since direct observation of fuels was not conducted. Furthermore, there is insufficient information on how to categorize mixed users. The kitchen may be in-house, a separate building, or cooking outdoors, and this study did not consider this category. | Probably High |
| Outcome assessment | Infant and under-five mortality refer to the probability of dying between birth and the first birthday and before the fifth birthday, respectively. Self-reporting may introduce recall bias or inaccuracies in reporting. | Probably low |
| Confounding bias | The study adjusted for other environmental variables such as household window, presence of pets in the house, type of latrine, and water source. Additionally, the study adjusted for the sex of the child, mother's education, wealth index, husband's occupation, and marital status of the mother. However, other potential confounding factors should also be considered, including birth interval, birth weight, vaccination status, breastfeeding status, overcrowding condition, place of residence, and other existing disease conditions. | Probably high |
| Incomplete outcome data | The study did not have incomplete outcome data | Low |
| Selective reporting | All the study’s specified outcomes were adequately reported. | Low |
| Conflict of interest | The study was funded and the authors declared no conflict of interest. | Probably Low |
| Other bias | The case-control study might demonstrate a stronger causal relationship; however, it carries a risk of recall bias. The all-cause mortality may include mortality outcomes that were not associated to researcher’s interest. | Probably Low |

| **#24: Shiferaw et al., 2023** | | |
| --- | --- | --- |
| **Design** | Cross-sectional | |
| **Participant** | All children under five who had data on child mortality and location coordinates | |
| **Exposure** | Exposure to ambient PM_2.5_ | |
| **Outcome** | Under-five mortality | |
| **Risk assessment** | **Supportive response** | **Author’s judgment** |
| Recruitment strategy | DHS data in the selected households whose mortality data and cluster’s Global Positioning System (GPS) coordinates were recorded in the EDHS dataset were included in this study. Children without mortality and geographical location coordinates information were excluded. The study selection process was unrelated to exposure or outcome status and no means to conduct bias in the selection of participants. | Low |
| Exposure assessment | A satellite-based estimate by the Atmospheric Composition Analysis Group at Washington and Dalhousie University determined the lifetime annual mean total PM_2.5_ concentration exposure for each child. For deceased children, this exposure spanned all months up to their death; for surviving children, it extended up to the month when EDHS 2016 data was collected. The geographical coordinates of Enumeration Areas (EAs) were matched with average annual total PM_2.5_ concentration data. However, the DHS randomly displaced EA coordinates up to 2 km in urban and 5–10 km in rural areas for confidentiality reasons. This displacement, coupled with the oversight of not considering individuals who may have moved out of EAs during the study period, may compromise the accurate representation of true average population exposure. | Probably High |
| Outcome assessment | The data for this study was derived from DHS, where mothers self-reported in interviews. Children reported as deceased before reaching their fifth birthday was categorized as under five mortalities. Despite being a significant event, there might be recall bias or inaccuracies in reporting. | Probably Low |
| Confounding bias | The author adjusted for sex, age, size at birth, birth order, the plurality of the child, and place of delivery. Additional the study adjusted for environmental factors (sources of drinking water, time to fetch, any kind of household water treatment, toilet facility), community level variables (residence and region). However, the study did not made adjustment for other important confounding variables such as maternal age and education status, vaccination status, breastfeeding status, malnutrition, overcrowding condition, household income, other existing disease condition. | Probably High |
| Incomplete outcome data | Due to the nature of the study's source, there was missing data. However, sensitivity analysis was employed to assess the influence of changes in the values of these missing variables on the bivariate relationship between the independent and dependent variables. The large sample size may also mitigate the potential impact of this issue. | Probably Low |
| Selective reporting | All the study’s specified outcomes were adequately reported. | Low |
| Conflict of interest | The author reported no financial relationships in the conduct of this study and further declared no conflict of interest. | Low |
| Other bias | Cause effect association may have been underestimated due to the cross-sectional nature of the data. The all-cause mortality may include mortality outcomes that were not associated to researcher’s interest. | Probably Low |

| **#25: Starnes et al., 2023** | | |
| --- | --- | --- |
| **Design** | **Cross-sectional** | |
| **Participant** | Under-five children | |
| **Exposure** | Exposure to indoor smoking | |
| **Outcome** | Under-five mortality | |
| **Risk assessment** | **Supportive response** | **Author’s judgment** |
| Recruitment strategy | The study employed a spin-the-bottle technique to randomly select households, ensuring consistency in participant recruitment across study groups. A hybrid sampling technique was utilized to obtain as random a sample as feasible, considering the logistical challenges and absence of a household-level sampling frame. GIS was employed to generate the center point of each grid cell, serving as the starting location for enumerators during the survey. The study selection process was independent of exposure or outcome status, minimizing bias in participant selection. | Probably Low |
| Exposure assessment | Self-reported data was collected, and exposure to indoor smoking was defined as the presence of in- house cooking stove without ventilation during data collection time. However, the study lacks assurance against recall bias, as direct observation of fuels was not conducted. Furthermore, there is insufficient information provided on how to categorize kitchen smoke, including the type of smoke and the type of kitchen used, including those both clean and polluted fuel users. | Probably High |
| Outcome assessment | Outcome data was obtained from mothers self-reported in interviews and a child reported as deceased before reaching their fifth birthday was categorized as under five mortalities and April to June births were categorized as rainy season whereas, Births during the long rainy season were defined as those occurring from April to June. Since the data relied on mother’s recollection of children’s birth and death, there is a risk of recall bias or inaccuracies in reporting, though it a major event. | Probably Low |
| Confounding bias | The study adjusted for age, sex, birth order, maternal age, maternal marital status, wealth index, birth season, gestation, region, and healthcare visits. However, it did not account for other important variables such as maternal education status, vaccination status, breastfeeding status, malnutrition, overcrowding condition, safety of drinking water, and urban versus rural residence. | Probably high |
| Incomplete outcome data | The study reported date of death data for 119 deaths children were missing and exempted from analysis. A sensitivity analysis performed with interview date as death date. | Probably Low |
| Selective reporting | All the study’s specified outcomes were adequately reported. | Low |
| Conflict of interest | The study was funded, and the authors did not declared conflict of interest. | Probably Low |
| Other bias | The study shares limitation of cross-sectional study design which show association at a given point in time. Furthermore, all-cause mortality may encompass outcomes that were not directly relevant to the researchers' interests. | Probably Low |

| **#26: Tazinya et al., 2018** | | |
| --- | --- | --- |
| **Design** | **Cross-sectional** | |
| **Participant** | Children between 2 and 59 months | |
| **Exposure** | Exposure to wood smoke and passive smoking | |
| **Outcome** | ARI | |
| **Risk assessment** | **Supportive response** | **Author’s judgment** |
| Recruitment strategy | All children under 5 years who visited Bamenda Regional Hospital during the study period were enrolled. Children under 2 months were excluded due to the low sensitivity and specificity of clinical definitions of ARIs in this age group, and their nonspecific clinical presentation. Consent was obtained for recruitment into the study. Case definition relied on the Integrated Management of Childhood Illnesses (IMCI) classification. However, there is no documentation regarding the selection of study participants; if researchers conducted the process, there might be a risk of selection bias. | Probably Low |
| Exposure assessment | Data was collected using a structured questionnaire on the demographic, clinical and socioeconomic variables of the child and the parents or guardians. exposure to wood smoke defined as any child who spends more than 30 min in wood smoke daily. passive smoking (any child living with someone who smokes at home). The study is susceptible to recall bias due to the nature of the data acquisition measurement employed, and it would be better to complement this method with direct observation of the fuels. Additionally, there is a lack of sufficient information exposure classification. | Probably High |
| Outcome assessment | The case definition for children presenting with cough or difficulty breathing was established according to the Integrated Management of Childhood Illnesses (IMCI) classification. It categorized cases into Mild ARI (without pneumonia), Moderate ARI (with pneumonia), and Severe ARI (with severe pneumonia). Diagnoses of the different ARIs were determined primarily through clinical findings. However, it's worth noting that comparing these clinical diagnoses to gold standards yields lower sensitivity and specificity. | Probably Low |
| Confounding bias | The study separately analysis the environmental factors and did adjust other potential confounding. | Probably high |
| Incomplete outcome data | The study did not have incomplete outcome data. | Low |
| Selective reporting | All the study’s specified outcomes were adequately reported. | Low |
| Conflict of interest | The study was not funded, and authors declared no known conflict of interest. | Low |
| Other bias | A longitudinal study would better illustrate the effects of the potential risk factors than this cross-sectional study. | Probably Low |

| **#27: Ujunwa et al., 2014** | | |
| --- | --- | --- |
| **Design** | **Cross-sectional** | |
| **Participant** | Under‑five children, | |
| **Exposure** | Exposure to wood biofuel and passive smoking | |
| **Outcome** | ARIs | |
| **Risk assessment** | **Supportive response** | **Author’s judgment** |
| Recruitment strategy | Children clinically diagnosed with any form of acute respiratory infection were consecutively enrolled in the study. Those above 5 years old with ARIs were excluded, as were children with chronic respiratory infections or diseases, foreign body aspiration, and non-respiratory pathologies. Recruitment occurred either at presentation or within 48 hours of admission for hospitalized cases, as determined by the attending physicians. However, there is no documentation regarding the procedure to blind doctors to the study. | Probably Low |
| Exposure assessment | Self-report from respondents using structured proforma. The study is susceptible to recall bias due to the nature of the data acquisition measurement employed, and it would be better to complement this method with direct observation of the fuels. Additionally, there is a lack of sufficient information exposure classification. | Probably High |
| Outcome assessment | The case definition adhered to the WHO working group's guidelines on the management of ARIs, which characterized ARI as a clinical condition featuring rapid breathing exceeding the anticipated upper limit for age, with or without signs of chest indrawing, inability to feed due to illness, nasal discharge, cough, fever, and auscultatory findings over a period of less than 2 weeks. The absence of a culture-proven diagnosis of pneumonia allows for the possibility of false positive cases. | Probably Low |
| Confounding bias | The study did adjust for potential confounding | high |
| Incomplete outcome data | The study did not have incomplete outcome data | Low |
| Selective reporting | All the study’s specified outcomes were adequately reported. | Low |
| Conflict of interest | The study was not funded, and authors declared no known conflict of interest. | Low |
| Other bias | A longitudinal study would better illustrate the effects of the potential risk factors than this cross-sectional study | Probably Low |

| **#28: Vanker et al., 2017** | | |
| --- | --- | --- |
| **Design** | **Cohort** | |
| **Participant** | Mother-infant pairs | |
| **Exposure** | 1. PM_10_ measured using a personal air sampling pump (AirChek)  2. CO measured using Altair (Troy)  3. Nitrogen dioxide, sulfur dioxide, and volatile organic compounds (benzene and toluene) measured in diffusion tubes in homes  4. Maternal smoking | |
| **Outcome** | Pneumonia | |
| **Risk assessment** | **Supportive response** | **Author’s judgment** |
| Recruitment strategy | The recruitment strategy for the Drakenstein Child Health Study (DCHS) is well-designed, emphasizing consecutive enrolment of pregnant women from diverse racial backgrounds, which enhances the representativeness of the sample. The extended 3-year enrolment period ensures constant participation across various seasons, minimizing temporal bias. However, the exclusion of women under 18, those not attending postnatal care, and those planning to relocate may limit generalizability. | Probably Low |
| Exposure assessment | The exposure assessment in the Drakenstein Child Health Study is thorough, utilizing both antenatal and postnatal home visits to measure indoor air pollution (IAP). By employing personal air sampling for PM_10_, carbon monoxide, and diffusion tubes for pollutants like nitrogen dioxide and volatile organic compounds, the study ensures accurate quantification of air quality. The alignment with South African National Ambient Air Quality Standards provides a solid framework for evaluating exposure levels. Additionally, the integration of questionnaires to assess environmental tobacco smoke (ETS) adds depth to the assessment with fear of some recall bias | Probably Low |
| Outcome assessment | The outcome assessment for lower respiratory tract infections (LRTIs) shows a thorough and methodical approach. Utilizing WHO case definitions ensures standardized diagnosis, while active surveillance, caregiver reports, and trained staff auscultation bolster data accuracy. The distinction between LRTI and wheeze episodes further minimizes misclassification risks. This rigorous assessment framework enhances the validity of findings, supporting reliable conclusions about respiratory disease incidence in the cohort. | Low |
| Confounding bias | The use of mixed-effects Poisson regression and univariate logistic regression effectively addresses the complexities of repeated measures and count data, enhancing the reliability of findings related to lower respiratory tract infections (LRTI) and wheezing. Additionally, the inclusion of a comprehensive range of confounding variables—such as birthweight, socioeconomic status, maternal HIV status, and household characteristics—indicates a thorough approach to identifying and controlling factors that could influence respiratory outcomes, thereby enhancing the validity of the associations drawn in the study. The application of appropriate statistical tests, including descriptive statistics, Wilcoxon rank-sum tests, and chi-square tests for group comparisons, reflects careful consideration of the data's distribution and nature. Adhering to a significance level of α=0.05 further reinforces the rigor of the statistical analysis, reducing the likelihood of false positives. | Probably low |
| Incomplete outcome data | The study did not have incomplete outcome data. | Low |
| Selective reporting | All the study’s specified outcomes were adequately reported. | Low |
| Conflict of interest | The study was funded but the funder had no role in the study design and overall analytical steps. and the authors declared no conflict of interest. | Probably Low |
| Other bias | Follow-up rates and participant retention throughout the study period may vary, leading to attrition bias. If those lost to follow-up have different health outcomes or exposure histories than those who remain, this could skew results. | Probably Low |

| **#29: Verani et al., 2016** | | |
| --- | --- | --- |
| **Design** | **Case-control** | |
| **Participant** | Children <5 years of age | |
| **Exposure** | secondhand smoke exposure | |
| **Outcome** | Presumed bacterial pneumonia | |
| **Risk assessment** | **Supportive response** | **Author’s judgment** |
| Recruitment strategy | Cases were identified from among children hospitalized with acute lower respiratory infection at Chris Hani Baragwanath Academic Hospital (CHBAH). Age-matched community controls were selected using CHBAH birth records within ±1 week of the case's birth date. The selection process was random, ensuring no bias in exposure or outcome status. However, selecting cases from hospitals and controls from the community may introduce inconsistencies across groups. It is possible that controls shared some unaccounted risk factors with cases. | Probably Low |
| Exposure assessment | study nurses interviewed the parent/guardian of enrolled cases and controls in person. Smoking is sensitive in some communities and there might be a reporting bias. | Probably High |
| Outcome assessment | Chest radiographs were reviewed and case outcomes were determined by a team of physicians trained in the World Health Organization approach for standardized interpretation. | Low |
| Confounding bias | The author adjusted for race, sex, malnutrition, heart disease, previous pneumonia hospitalization, mother HIV infected, exclusive breastfeeding up to age 4 months, water source indoor tap and crowding. However, the study did not made adjustment for other important confounding variables such as maternal age and education status, birth-interval, birthweight, vaccination status, place of residence, household income, indoor and indoor cooking source | Probably high |
| Incomplete outcome data | The outcomes focused on pediatric bacterial pneumonia (PBP) cases among human immunodeficiency virus (HIV) uninfected children with lower respiratory tract infection. However, maternal HIV status was obtained through interviews, which could potentially result in underreporting and influence the outcome data. | Probably high |
| Selective reporting | All the study’s specified outcomes were adequately reported. | Low |
| Conflict of interest | The study was funded, and the funder did not exert any influence on the findings and conclusion of the study. | Probably Low |
| Other bias | The case-control study might demonstrate a stronger causal relationship; however, it carries a risk of recall bias. The institutionalized nature of the study might compromise its true representativeness of the condition within the general population. | Probably Low |

| **#30: Wichmann et al., 2006** | | |
| --- | --- | --- |
| **Design** | **Cross-sectional** | |
| **Participant** | Under five years children | |
| **Exposure** | Exposure to cooking and space heating smoke. | |
| **Outcome** | 1–59-Month-Old Mortality | |
| **Risk assessment** | **Supportive response** | **Author’s judgment** |
| Recruitment strategy | The study used data from 1998 SADHS which was a national household survey of the population living in private households in all nine provinces of South Africa. The study selection process was independent to exposure or outcome status and no means to conduct bias in the selection of participants | Low |
| Exposure assessment | The study utilized data gathered through a questionnaire asking about household cooking and heating methods. Households were categorized into two groups: those using polluting fuels (wood, dung, coal, or paraffin without LPG/natural gas or electricity) and those using clean fuels (exclusive use of LPG/natural gas or electricity). However, there is a lack of assurance against recall bias as direct observation of fuels was not conducted, and categorization for mixed users was not specified. Additionally, the study did not consider other potential cooking fuel sources such as charcoal, crop residues, or straw/shrubs. | Probably High |
| Outcome assessment | The outcome variable was determined by querying women about their childbirth experiences and whether their children were alive, and if not, at what age they had passed away. Despite being a major event, there remains a risk of recall bias or inaccurate reporting. | Probably Low |
| Confounding bias | The study adjusted for maternal age, water source, crowding, and asset index. However, it did not make adjustments for other important confounding variables such as maternal education status, birth interval, birthweight, vaccination status, breastfeeding status, malnutrition, place of residence, and other existing disease conditions. | Probably High |
| Incomplete outcome data | The nature of the data source limited the analysis to complete data. However, the large sample size helps overcome the problem of missing data. | Probably Low |
| Selective reporting | All the study’s specified outcomes were adequately reported. | Low |
| Conflict of interest | The study was tot funded and conflict of interest declared. | Low |
| Othe bias | The cross-sectional nature of the data may have led to an underestimation of cause-effect associations. Furthermore, all-cause mortality may include outcomes that were not directly relevant to the researchers' interests. | Probably Low |

| **#31: Winterbach et al., 2021** | | |
| --- | --- | --- |
| **Design** | **Retrospective Cross-sectional** | |
| **Participant** | Infants | |
| **Exposure** | Exposure to cigarette smoke | |
| **Outcome** | Sudden unexpected death of infants, | |
| **Risk assessment** | **Supportive response** | **Author’s judgment** |
| Recruitment strategy | All sudden unexpected death in infancy (SUDI) admissions to the FPS mortuaries within these areas from January 1, 2012, to December 31, 2016, were reviewed. The study selection process was independent of exposure or outcome status, ensuring no bias in participant selection. While vital registration data is the preferred source for child mortality data, no further exploration about how well it functions and who recruits. | Probably Low |
| Exposure assessment | Data were collected from the original case files in the archives of each mortuary and/or from their electronic equivalents stored on Livelink (Open Text, Canada). However, measuring smoking through simple interviewing may lead to underreporting, as it is sensitive in some communities. | Probably High |
| Outcome assessment | Data were collected from the original case files in the archives of each mortuary and/or from their electronic equivalents stored on Livelink (Open Text, Canada). While vital registration data is the preferred source for child mortality data, no further exploration about how well it functions and who recruits. | Probably Low |
| Confounding bias | No adjustments were made for potential confounding variables. | High |
| Incomplete outcome data | The study was constrained by the information available in the case files. However, it is widely recognized that vital registration data is the preferred source for child mortality data, surpassing population census, household surveys, sample registration systems, and surveillance sites. | Probably Low |
| Selective reporting | All the study’s specified outcomes were adequately reported. | Low |
| Conflict of interest | The study had no funding source, and the authors declared no conflicts of interest. | Low |
| Other sources of bias | The descriptive, retrospective nature of this study precludes the calculation of the prevalence of the SIDS/SUDI risk factors in the entire infant population. | Probably high |
