# Supplementary material for: The effect of air pollution on morbidity and mortality among children aged under five in sub-Saharan Africa: Systematic review and meta-analysis
Source: PLoS One. 2025 Apr 10;20(4):e0320048. doi: 10.1371/journal.pone.0320048 (PMC11984980; doi:10.1371/journal.pone.0320048)
Supplement: S6 File — (DOCX) [file pone.0320048.s006.docx]

## **Supplementary file S**6: Meta-analysis

**Supplementary** **Table1 S6: Leave-one-out sensitivity analysis of meta-analyzed associations between exposure to solid fuel smoke and all-cause under-five year mortality.**

| **Omitted study** | **Age of study participants (in months)** | **OR** | **(95% CI)** | | **P-value** |
| --- | --- | --- | --- | --- | --- |
| Akinyemi et al., (2016) | <12 | 1.30 | 1.15 | 1.47 | <0.0001 |
| Akinyemi et al., (2016) | 12-59 | 1.32 | 1.16 | 1.49 | <0.0001 |
| Bickton et al., (2020) | 0-59 | 1.31 | 1.15 | 1.48 | <0.0001 |
| Ezeh et al., (2014) | <1 | 1.33 | 1.18 | 1.51 | <0.0001 |
| Ezeh et al., (2014) | 1-11 | 1.27 | 1.14 | 1.42 | <0.0001 |
| Ezeh et al., (2014) | 12-59 | 1.29 | 1.15 | 1.46 | <0.0001 |
| Flanagan., (2022) | 0-59 | 1.32 | 1.17 | 1.48 | <0.0001 |
| Imo et al., (2023) | 0-59 | 1.32 | 1.16 | 1.51 | <0.0001 |
| Kleimola et al., (2015) | <1 | 1.34 | 1.18 | 1.52 | <0.0001 |
| Kleimola et al., (2015) | 1-59 | 1.34 | 1.19 | 1.52 | <0.0001 |
| Latona et al., (2017) | 0-59 | 1.24 | 1.13 | 1.36 | 0.001 |
| Owili et al., (2017) | 0-59 | 1.33 | 1.16 | 1.52 | <0.0001 |
| Samuel et al., (2018) | 0-59 | 1.32 | 1.16 | 1.49 | <0.0001 |
| Wichmann et al., (2006) | 1-59 | 1.29 | 1.15 | 1.45 | <0.0001 |
| D+L pooled ES |  | 1.31 | 1.16 | 1.47 | <0.0001 |

**Supplementary** **Fig1 S6: Contour-enhanced funnel plot of the meta-analyzed association between exposure to solid fuel smoke and all-cause under-five mortality (X-axis -logarithmic scale of OR (logOR) Y-axis-standard error of logOR).**

**Supplementary** **fig2 S6: Subgroup analysis pre and post 2015 of meta-analyzed associations between exposure to solid fuel smoke and all-cause under-five years mortality.**

**Supplementary** **fig3 S6: Subgroup analysis by study setting of meta-analysed association between exposure to solid fuel combustion and all-cause under-five years mortality.**

**Supplementary fig4 S6: Forest plot of meta-analysed association between exposure to solid fuel combustion and all-cause under-five years mortality, excluding "high quality" study based on ROB key quality appraisal.**

.

**Supplementary fig5 S6: Trim and-fill analysis of meta-analysed association between exposure to solid fuel combustion and all-cause under-five years mortality.**

**Supplementary fig6 S6: Forest plot of meta-analysed associations between exposure to passive smoking and pneumonia in children under-five years.**

**Supplementary fig7 S6: Forest plot of meta-analysed associations between exposure to secondhand smoking and pneumonia in children under five years, excluding "low quality" study based on ROB key quality appraisal.**

**Supplementary** **Table2 S6: Leave-one-out sensitivity analysis of meta-analyzed associations between exposure to passive smoking and pneumonia in children under five years**

| **Omitted study** | **Country** | **OR** | **(95% CI)** | | **P-value** |
| --- | --- | --- | --- | --- | --- |
| Dano (2019) | Niger | 1.51 | 0.71 | 3.18 | <0.0001 |
| Kiconco (2021) | Uganda | 1.14 | 0.53 | 2.45 | <0.0001 |
| Ngocho (2019) | Tanzania | 1.66 | 0.84 | 3.29 | <0.0001 |
| PrayGod (2016) | Tanzania | 1.42 | 0.70 | 2.92 | <0.0001 |
| Roux (2015) | South Africa | 1.43 | 0.70 | 2.93 | <0.0001 |
| Roux (2021) | South Africa | 1.17 | 0.45 | 3.06 | <0.0001 |
| Verani (2016) | South Africa | 1.02 | 0.53 | 1.99 | <0.0001 |
| D+L pooled ES |  | 1.32 | 0.67 | 2.62 | <0.0001 |
