# Supplementary material for: The effect of air pollution on morbidity and mortality among children aged under five in sub-Saharan Africa: Systematic review and meta-analysis
Source: PLoS One. 2025 Apr 10;20(4):e0320048. doi: 10.1371/journal.pone.0320048 (PMC11984980; doi:10.1371/journal.pone.0320048)
Supplement: S7 File — (DOCX) [file pone.0320048.s007.docx]

# **Supplementary** file S7: Certainty of evidence assessment

**Supplementary** **table1 S7: Factors for evaluation of quality, and strength of the body of evidence**

| **Evaluation factors** | **Summary of criteria** |
| --- | --- |
| ***Downgrading factors*** | |
| Risk of bias | Study limitations include a substantial risk of bias across the body of evidence. Risk of bias was assessed by sensitivity analyses, excluding studies rated “high” and/or “low” risk of bias for key domains. The quality of body of evidence was downgraded if there was substantial difference between values of sensitivity analysis. |
| Indirectness | Evidence was not directly comparable to the primary objective of interest i.e., participants, exposure, comparisons, outcome (PECO). |
| Inconsistency | Estimates of effect in similar populations were widely different (significantly high heterogeneity *I^2^* or variability in results). In addition, the evidence was downgraded due to variation in effect estimates based on sensitivity analysis. |
| Imprecision | Studies included few participants and small sample sizes (wide confidence interval as judged by reviewers). |
| Publication bias | Studies were missing from body of evidence, resulting in an over- or underestimate of true effects from exposure. The evidence of publication bias was inspected visually in the funnel plots and egger’s test. The *Trim and Fill* procedure was used to estimate potentially missing studies. |
| ***Upgrading factors*** | |
| Large magnitude of effect | The rating was upgraded if modeling suggested that confounding alone was unlikely to explain associations that were judged to be of large magnitude. |
| Dose response | Upgraded if consistent relationship between dose and response in one or multiple studies, and/or the dose response across studies. |
| Confounding minimizes effect | Upgraded if the consideration of all plausible residual confounders or biases would underestimate the effect or suggest a spurious effect when results show no effect. |

**Supplementary** **table2 S7: Rating of evidence quality for meta-analyses on solid fuel use and under-five mortality,**

**and secondhand smoking and pneumonia**

|  | **Solid fuel and under-five mortality studies _(_n=14)** | **Maternal smoking and under-five pneumonia studies (n=7)** |
| --- | --- | --- |
| **Initial rating** | Moderate | Moderate |
| Risk of bias across studies | 0 | 0 |
| Indirectness | -1 | -1 |
| Inconsistency | 0 | -1 |
| Imprecision | 0 | -1 |
| Publication bias | 0 | 0 |
| Large magnitude of effect | 0 | 0 |
| Dose response | +1 | +1 |
| Confounding minimizes effect | 0 | 0 |
| Resulting rating | **Moderate** | **Very low** |

**Notes**: 0=no downgrade or upgrade. –1=downgrade. +1=upgrade. Detailed information is presented below (P30).

**Supplementary** **table3 S7:** **Quality and strength of evidence on meta-analysis associations between solid fuel use and under-five mortality, and secondhand smoking and pneumonia**

|  | **Particulate pollutant studies** | | | |
| --- | --- | --- | --- | --- |
|  | **Solid fuel and under-five mortality studies _(_n=14)** | | **Maternal smoking and under-five pneumonia studies (n=7)** | |
|  | **Ratings** | **Rationale** | **Ratings** | **Rationale** |
| -Risk of bias across studies | (0) | One study had a “high” risk of bias for confounding control, sensitivity analysis by excluding this study doesn’t alter significantly the overall estimate. We judged there is no substantial difference because of risk of bias across all studies. | (0) | One study had a “low” risk of bias for confounding control, though the rest rated “probably high risk.” sensitivity analysis by excluding this study doesn’t significantly alter the overall estimate. We judged there is no substantial difference because of the risk of bias across all studies. |
| -Indirectness | (-1) | All 14 studies used self-reported DHS data on under-five mortality and cooking fuel type. While self-reported data may limit precision, making it harder to isolate the effect of solid fuel use, it raises questions about the direct comparability of the evidence to the study’s focus (population, exposure, comparator, outcome). | (-1) | Most studies (5/7) identified pneumonia using WHO case definitions, and two further used radiographic checks with physician decision (2/7) and self-report measures of exposure variables. Smoking is sensitive in some communities, leading to underreporting and bias in self-reported data, reducing exposure accuracy, which may limit the direct causal link to the study's objectives (population, exposure, outcome). |
| -Inconsistency | (0) | The evidence of quality was not downgraded for inconsistencies. The *I^2^* value of heterogeneity was found to be *I^2^* = 78.6. %. We found no difference in the pooled odds ratios (ORs) in the leave-one-out sensitivity analysis. | (-1) | The evidence of quality was downgraded for inconsistencies. The *I^2^* value of heterogeneity was found to be *I^2^* = 88.6%. The inclusion of diverse study designs (two cross-sectional, two cohort, and four case-control) may contribute to variability in findings |
| -Imprecision | (0) | We judged that the number of included events is sufficient for meta-analysis, and the confidence level in the pooled estimate is sufficiently narrow. | (-1) | We judged that the number of included events is insufficient for meta-analysis. The confidence in the pooled estimates is sufficiently narrow. |
| -Publication Bias | (0) | The funnel plot (figure 4) and objective measurement of Egger’s test suggest no significant publication bias (P= 0.110).  Therefore, we did not downgrade the quality of evidence for the potential risk of publication bias. | (0) | *Egger's* test suggests no significant publication bias (*p* = 0·3.68). Therefore, we did not downgrade the quality of evidence for the potential risk of publication bias. |

| -Large magnitude of effect | (0) | We did not consider the estimated effects large, as the overall effect estimate (pooled OR) was below 2. | (0) | We did not consider the estimated effects large, as the overall effect estimate (pooled RR) was below 2. |
| --- | --- | --- | --- | --- |
| -Dose-response | (+1) | Studies suggested an exposure-response gradient, i.e., exposure to solid fuel increases the risk of under five mortality in most instances. | (+1) | Studies suggested an exposure-response gradient, i.e., exposure to secondhand smoking increases the risk of under-five pneumonia in most instances. |
| -Confounding minimizes the effect | (0) | We identified and acknowledged that included studies might have residual confounding because they did not adjust for all important well-studied confounders. However, we found no evidence to suggest that possible residual confounders would shift the effect to null. | (0) | We identified and acknowledged that some studies might have residual confounding because they did not adjust for all important well studied confounders. These possible residual-confounders would affect the effect estimate and tended towards positive association. |
| -Overall quality of evidence (initial rating is **“moderate**”) | Moderate | Moderate + (1) +(-1)= moderate  One downgrading (-1) and one upgrading (+1), kept the quality of studies to the initial rating “moderate”. | Very low | Moderate + (+1) + (-1) + (-1) +(-1)= very low  Three downgrades (-1) and one upgrade (+1) brought the quality of studies to “very low.” |
| -Summary of findings for meta-analysis | n/a | Studies included in the meta-analysis provided consistent results, i.e., the risk of under-five mortality increases with solid fuel exposure among under-five children. | n/a | Studies included in the meta-analysis provided consistent results, i.e., the risk of pneumonia increases with secondhand smoking exposure, although the 95%CI includes the null in four of the seven studies. |
| Quality of evidence | n/a | Moderate | n/a | Very low |
| -Direction of effect estimates | n/a | The risk of under-five mortality increases among under-five children with increasing exposure to solid fuel. | n/a | The risk of pneumonia increases among the under-five children with increasing exposure to secondhand smoking, although the 95% CI includes the null/not statistically significant. |
| -Confidence in effect estimates | n/a | We believe that our conclusion is unlikely to be strongly affected by the results of future studies i.e., shift the results of the meta-analysis null or insignificant. | n/a | We believe that our conclusion is likely to be affected by the results of future studies i.e., shift the results of the meta-analysis significant/towards positive association. |
| -Other aspects | n/a | None | n/a | None |
| -Overall strength of evidence | Sufficient | Overall, we rated the strength of the evidence as “sufficient.” We found that there is a positive association between solid fuel use and an increased risk of under-five mortality. The evidence is derived from a substantial number of well-designed and well-conducted national representative studies, leading us to conclude that our findings are unlikely to be significantly impacted by future research. | Inadequate | Overall, we rated the strength of the evidence as not sufficient. We found a positive association between secondhand smoking and pneumonia, but the 95% confidence interval includes the null. The available evidence was based on results from a few studies; thus, we believe that our conclusion is likely to be affected by the results of future studies. |

**Footnote**: Initial certainty ratings for associations were set at moderate quality based on prior recommendations(1, 2). These ratings could be downgraded due to concerns about risk of bias, inconsistency (contradictory findings), imprecision (wide confidence intervals or few studies), indirectness (non-direct measurement of exposures or outcomes), and publication bias. Conversely, ratings could be upgraded for evidence of dose-response relationships, large effect sizes, or biases that likely underestimated the observed effect. Following adjustments, associations received final ratings (high, moderate, low, or very low) reflecting our confidence in the evidence and conclusions. In this study, the overall quality of evidence was rated as "moderate" for solid fuel use and under-five mortality, but downgraded to "very low" for secondhand smoking and pneumonia in under-five children due to indirectness (self-reported data), imprecision (insufficient studies), and inconsistency (variability in effect sizes and study designs).

# References

1. Gabet S, Lemarchand C, Guenel P, Slama R. Breast Cancer Risk in Association with Atmospheric Pollution Exposure: A Meta-Analysis of Effect Estimates Followed by a Health Impact Assessment. Environ Health Perspect. 2021;129(5):57012.

2. OHAT N. Handbook for conducting a literature-based health assessment using OHAT approach for systematic review and evidence integration. US National Toxicology Program Office of Health Assessment and Translation Available online at <https://ntp> niehs nih gov/pubhealth/hat/review/index-2 html, checked on. 2019;5(8):2019.
